# Re-Evaluation of the Relationship Between Average Nucleotide Identity and dDDH Values in the Genus *Micromonospora*, and Description of *Micromonospora cynarisoli* sp. nov., a Novel Actinobacterium from the Rhizosphere Soil of *Cynara scolymus*

**DOI:** 10.3390/microorganisms14050981

**Published:** 2026-04-27

**Authors:** Kaiqin Li, Li Fu, Peilan Long, Ying Qian, Wei Liang, Jian Gao

**Affiliations:** 1 School of Computer Science and Engineering, Hunan University of Science and Technology, Xiangtan 411201, China; wliang@hnust.edu.cn; 2 School of Life and Health Sciences, Hunan University of Science and Technology, Xiangtan 411201, China; fuli24@whu.edu.cn (L.F.); lpl@mail.hnust.edu.cn (P.L.); xtgojian@hnust.edu.cn (J.G.); 3 Key Laboratory of Ecological Remediation and Safe Utilization of Heavy Metal-Polluted Soils, College of Hunan Province, Xiangtan 411201, China

**Keywords:** *Micromonospora*, ANIm and dDDH, *Micromonospora cynarisoli* sp. nov, heterotypic synonym, polyphasic method

## Abstract

It is widely accepted in prokaryotic systematics that a 95–96% ANI (average nucleotide identity) value is equivalent to 70% dDDH (digital DNA–DNA hybridization) value in prokaryotic systematics. However, we recently found that a 70% dDDH value was equivalent to an approximately 96.7% ANIm value in the genus *Micromonospora* based on a correlation analysis between dDDH and ANIm from a total of 1770 pairs of type *Micromonospora* strains (60 type strains). Therefore, we proposed that 96.7% ANIm (ANI based on the MUMmer algorithm) value could act as the threshold value in delineating *Micromonospora* species. Meanwhile, the taxonomic status of an actinobacterial strain HUAS LYJ1^T^, isolated from the rhizosphere soil of *Camellia oleifera*, was determined by using a polyphasic method. A 16S rRNA gene sequence analysis indicated that strain HUAS LYJ1^T^ shared the highest similarity to *Micromonospora wenchangensis* CCTCC AA 2012002^T^ (99.3%). Phylogenetic trees based on 16S rRNA gene and whole-genome sequences demonstrated that strain HUAS LYJ1^T^ was most closely related to *M. wenchangensis* CCTCC AA 2012002^T^. However, the ANIm value between them was 95.77%, below the 96.7% cut-off point recommended above; the dDDH value between them was 63.2%, also far below the 70% threshold value in delineating bacterial species. Based on these molecular data, as well as phenotypic and chemotaxonomical features, it is concluded that strain HUAS LYJ1^T^ represents a novel *Micromonospora* species, for which the name *Micromonospora cynarisoli* sp. nov. is proposed. In addition, it was found that the ANIm and dDDH values of *Micromonospora haikouensis* DSM 45626^T^, *Micromonospora harpali* NEAU-JC6^T^ and *Micromonospora oryzae* DSM 102119^T^ were 97.21–97.86% and dDDH 73.9–79.6%, respectively, above the 96.6% ANIm and 70% dDDH threshold value in delineating *Micromonospora* species. Consequently, according to rule 42 of the International Committee on Systematics of Prokaryote Code, we propose that *M. harpali* Fang et al. 2015 and *M. oryzae* Kittiwongwattana et al. 2015 are later heterotypic synonyms of *M. haikouensis* Xie et al. 2012.

## 1. Introduction

In microbial taxonomy, genomic methods have become crucial in the identification of novel species, with algorithms such as Average Nucleotide Identity (ANI) and digital DNA–DNA hybridization (dDDH) being widely employed to delineate species boundaries. The currently accepted threshold values in delineating species are ANI of 95–96% [1] and dDDH of 70% [2]. In other words, those strains, sharing an ANI of ≥95–96% or ≥dDDH of 70%, belong to the same species; those strains, sharing an ANI of <95–96% or <dDDH of 70%, belong to the different species. However, a growing body of research suggests that these two metrics may yield inconsistent results across different taxonomic groups, leading to conflicting taxonomic conclusions [3,4].

*Micromonospora* is a significant source of biologically active natural products [5,6,7,8] and an ideal model for studying microbial species boundaries. Our preliminary analysis revealed that the ANIm (ANI based on the MUMmer algorithm) value between *M. arida* LB32^T^ and *M. saelicesensis* DSM 44871^T^ is 96.33%, which is above the conventional threshold for species delineation. However, their dDDH value was only 68.4%, below the 70% threshold. This discrepancy highlights the uncertainties in applying current classification frameworks to this genus. Consequently, it remains to be investigated whether systematic deviations exist between ANIm and dDDH values across taxonomic units, and how the current thresholds might be revised to achieve more consistent species delineation.

To address these questions, this study aims to systematically evaluate the taxonomic relationships among several species within the genus *Micromonospora* using comparative genomic methods, with a focus on analyzing the compatibility and discrepancies between ANIm and dDDH algorithms in species demarcation. Additionally, a polyphasic taxonomic approach was applied to characterize a novel strain HUAS LYJ1^T^ and to reassess the phylogenetic relationships among *Micromonospora haikouensis* DSM 45626^T^, *Micromonospora oryzae* DSM 102119^T^, and *Micromonospora harpali* NEAU-JC6^T^, thereby contributing to a refined taxonomic framework for the genus.

## 2. Materials and Methods

### 2.1. Relationship Between ANIm and dDDH in the Genus Micromonospora

In order to obtain an accurate relationship between dDDH and ANI, we downloaded the genomes of 135 type *Micromonospora* strains (all validly published under the ICNP) from the GenBank database (as of Jan 2026), and then performed a quality analysis of these genomes. Only those genomes whose quality meets the criteria of >90% completeness and <5% contamination [9] can be used for subsequent analyses. Genome completeness and contamination were evaluated using CheckM (version 1.2.2) with the lineage_wf workflow. The analysis was run with the following parameters: lineage wf-t8-xfasta-tab table. The lineage wf automatically infers the appropriate marker set for each genome based on its phylogenetic lineage, ensuring accurate completeness and contamination estimates. The t8 option enables parallel processing to accelerate computation, x fasta specifies the input file format, and tab table generates a tab-delimited output for easy downstream processing. Genomes with completeness >90% and contamination <5% were retained for subsequent analyses, following the minimum quality standards recommended for high-quality bacterial genomes [10]. These thresholds ensure that only sufficiently complete and minimally contaminated genomes are included, reducing the risk of phylogenetic artifacts introduced by low-quality assemblies. Finally, a total number of 60 *Micromonospora* genomes with validly published names were obtained and their quality analysis and GenBank assembly of genomes is shown in Supplementary Table S1.

The calculation of the ANIm and dDDH values were done using the JSpeciesWS online service described by Richter et al. [11] and the Genome-to-Genome Distance Calculating described by Meier-Kolthoff et al. [12], respectively. The correlation analysis between ANIm and dDDH values was carried out according to the methods of Hu et al [3].

### 2.2. Evaluation of the Taxonomic Status of Strain HUAS LYJ1^T^

#### 2.2.1. Isolation of Strain HUAS LYJ1^T^ and Source of the Type Strain

The rhizosphere soil of *Camellia oleifera* was collected from Taoyuan County, Hunan Province, China (28°24′24″ N, 10°51′47″ E). The soil samples were placed indoors and air-dried at room temperature. Then, the dilution plate method was used to obtain the microbial isolates on modified Gauze’s synthetic medium [13] which contained (g/L): carboxy methycellulose-Na 5.0 g, KNO_3_ 1.0 g, MgSO_4_7H_2_O 0.5 g, NaCl 0.5 g, FeSO_4_7H_2_O 0.01 g, K_2_HPO_4_3H_2_O 0.5 g, agar 15.0 g, H_2_O 1.0 L, pH7.2, supplemented with 2.0–3.0 mL of K_2_Cr_2_O_7_ solution (1.775 g/L) in a 100 mL medium (All chemicals and reagents are from Hunan province, China). After 7 days of incubation at 28 °C, all *Micromonospora*-like colonies were selected and purified on Gauze’s synthetic medium [14]. One of the *Micromonospora*-like strains, strain HUAS LYJ1^T^, was maintained using a vacuum freeze-drying technology for long-term preservation. The type strains *Micromonospora wenchangensis* CCTCC AA 2012002^T^ and *Micromonospora mangrovi* CCTCC AA 2012012^T^ were purchased from China Center for Type Culture Collection (CCTCC). The type strain *M. oryzae* DSM 102119^T^ was purchased from the DSMZ-German Collection of Microorganisms and Cell Cultures GmbH (DSMZ). The type strain *M. harpali* NEAU-JC6^T^ was provided by Professor W.S. Xiang as a gift from Northeast Agricultural University. *M. wenchangensis* CCTCC AA 2012002^T^ was cultured under the same conditions for comparative analyses.

#### 2.2.2. Genome Sequencing and Phylogeny

For primary identification [15,16], four genomic DNAs were sequenced and assembled by the Nanopore PromethION sequencing system and SOAPnuke (version 2.1.2), which was carried out by Wuhan Benagen Technology Co., Ltd. (Wuhan city, Huibei Province, China). The full-length 16S rRNA gene sequences of strain HUAS LYJ1^T^ were extracted from whole-genome sequences. A similarity search of 16S rRNA gene sequences was carried out using the EzTaxon server (www.ezbiocloud.net/eztaxon) [17] (July 2025). The nearly full-length 16S rRNA gene sequence of strain HUAS LYJ1^T^ was directly sequenced using an automated DNA sequencing system (ABI 3730XL; Applied Biosystems), carried by the Sangon Biotech (Shanghai, China). The whole-genome sequencing of strain HUAS LYJ1^T^, *M. mangrovi* CCTCC AA 2012012^T^, *M. oryzae* DSM 102119^T^ and *M. harpali* NEAU-JC6^T^ was carried out using a Nanopore PromethION sequencing system, and then high-quality reads were assembled with SOAPnuke (Version: 2.1.2) by Wuhan Benagen Technology Co., Ltd. (Wuhan city, Hubei province, China). Phylogenetic trees based on 16S rRNA gene sequences were inferred using MEGA software (version, 11.0) [18] with the neighbor-joining (NJ) [19], maximum-likelihood (ML) [20], and maximum-parsimony (MP) [21] methods. The topology of phylogenetic trees was evaluated using 1000-replicate bootstrap analysis [22]. Phylogenomic tree was constructed using the Type (Strain) Genome Server [23]. Tree was inferred with FastME 2.1.6.1 [24] from GBDP (Genome Blast Distance Phylogeny) distances calculated from genome sequences. The branch lengths are scaled in terms of GBDP distance formula d5, which is a key computational framework for microbial classification. It calculates pairwise genome distances to generate both phylogenetic trees and standard digital DNA-DNA hybridization (dDDH) values used for species delineation. Branch support in GBDP trees is statistically evaluated through pseudo-bootstrap analysis, as cited. The numbers above branches are GBDP pseudo-bootstrap support values >60% from 100 replications, with an average branch support of 96.0%. The tree was rooted at the midpoint [25].

#### 2.2.3. Phenotypic Characteristics

After being grown on Reasoner’ 2A (R2A) [26] agar using the insert method at 28 °C for 14 days, morphological features were observed by light microscopy (NE620, Ningbo Yongxin Optics CO., LTD, Changsha City, Hunan Province, China) and scanning electron microscope (JSM-6610LV, Japan), respectively. After 21 days at 28 °C, cultural characteristics were observed on Gauze’s synthetic medium, R2A and ISP 2–7(International *Streptomyces* Project.) series media [27]; and then the color of aerial mycelium, substrate mycelium and soluble pigments was determined with the Color Standard and Color Nomenclature (basically includes all colors as positive controls) [28]. Sole carbon and nitrogen sources, a minimal medium (per liter: 1.0 g (NH_4_)_2_SO_4_, 1.0 g KH_2_PO_4_, 0.5 g MgSO_4_·7H_2_O, 0.5 g NaCl, pH 7.0), was used as the basal medium. Carbon sources were added at 0.5% (*w*/*v*); nitrogen sources were added at 0.1% (*w*/*v*). The medium without any carbon or nitrogen source served as a negative control. Cultures were incubated at 30 °C for 48 h with shaking (180 rpm). The effects of different temperatures (4–45 °C), pH values (pH 2.0–12.0 at intervals of 1.0 pH units) and NaCl concentrations (0–15%, *w*/*v*) on growth, and the other phenotypic characteristics experiments, were tested as described by Xu et al. [29].

Enzyme-activity tests were carried out using API ZYM strips purchased in Changsha City, Hunan Province (bioMérieux, France), according to the manufacturer’s instructions.

#### 2.2.4. Chemotaxonomical Characteristics

Cells of strain HUAS LYJ1^T^ and the reference strain *M. wenchangensis* CCTCC AA 2012002^T^ were grown in trypticase soy broth (TSB) on a rotary shaker for 5 days at 28 °C, and then were collected by centrifugation and washed three times with sterile water, freeze-dried by using a vacuum freeze-drying apparatus. Cellular fatty acids component analysis was carried out by the Marine Culture Collection of China (MCCC) (Xiamen City) (http://www.midi-inc.com/). The cell phospholipids were extracted, and then examined by two-dimensional thin-layer chromatography (TLC) and identified by four different chromogens, including 10% ethanolic molybdophosphoric acid, ninhydrin, anise aldehyde and molybdenum blue reagent [30]. The plate dotted with sample was subjected to two-dimensional development, with the first solvent of chloroform–methanol–water (65:25:4, *v*/*v*/*v*) followed by the second solvent of chloroform–methanol–acetic acid–water (80:18:12:5, *v*/*v*/*v*/*v*). The isomers of diaminopimelic acid (DAP) in the cell wall, whole-cell sugars in the whole-cell hydrolysates and dominant menaquinones were analyzed as described previously [30]. Strain HUAS LYJ1^T^ and strain *M. wenchangensis* CCTCC AA 2012002^T^ were inoculated into R2A liquid medium, cultured at an appropriate temperature and 120 rpm/min for about a week, centrifuged to collect the cells, washed twice with distilled water and centrifuged to retain the cells; then, the cells were lyophilized until they were completely dry. Then, the cells were used for MALDI-TOF MS (Matrix-Assisted Laser Desorption Ionization–Time-of-Flight Mass Spectrometry) analysis, carried out by China Center of Industrial Culture Collection (CICC, China) according to the protocol of the Methods for the Identification of Cultured Microorganisms Using MALDI-TOF MS (GB/T 33682-2017).

## 3. Results and Discussion

### 3.1. Relationship Between ANIm and dDDH in the Genus Micromonospora

**Figure 1:**
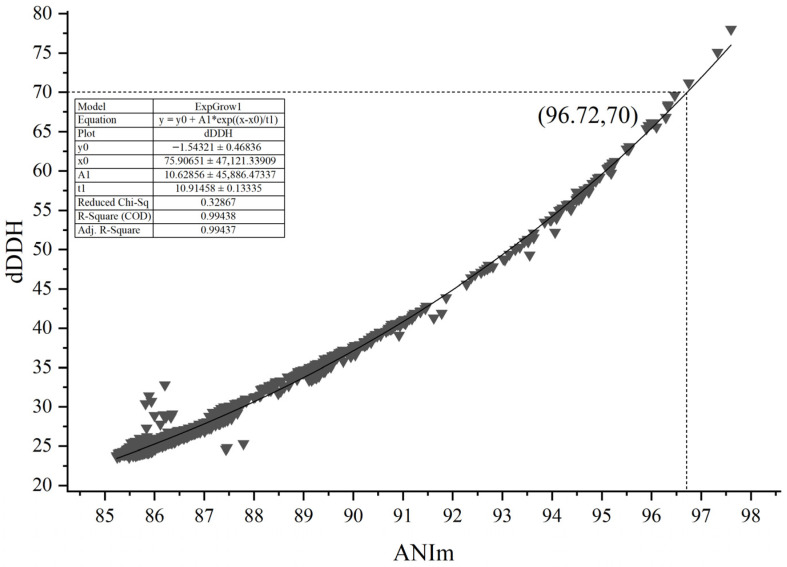
The correlation between ANIm and dDDH from the 1770 pairs of *Micromonospora* species. ANIm (Average Nucleotide Identity based on MUMmer alignment) and dDDH (digital DNA–DNA hybridization) are genome-based metrics for species delineation. dDDH values ≥70% are generally considered the threshold for assigning two genomes to the same prokaryotic species.

To resolve the contradictory phenomenon mentioned in the introduction, the ANIm and dDDH values of 60 type *Micromonospora* strains were selected for correlation analysis with a total of 1770 datasets by calculating pairwise between the strains (Table S2). As shown in Figure 1, the dDDH value showed an extremely high correlation (R^2^ = 0.99438) with the ANIm value based on an exponential regression model. However, a 70% dDDH value was not equivalent to a 95–96% ANIm value, but to an approximately 96.72% ANIm value. At present, in addition to ANIm, ANIb (based on the OrthoANIu algorithm) is also often used for evaluating genomic correlations between two strains. Similarly, in this genus, a 70% dDDH value did not correspond to a 95–96% ANIb value, but to an approximately 96.15% ANIb (Figure S1), and the dDDH value showed an extremely high correlation (R^2^ = 0.99245) with the ANIm value based on an exponential regression model (The R^2^ values are presented only as descriptive measures of goodness-of-fit for the exponential regression models; no statistical significance (*p*-values) is inferred due to the non-independence of pairwise comparisons). It can be seen that an ANI value of 95–96% does not correspond to a dDDH value of 70% in the genus *Micromonospora*. As the R^2^ in the ANIm fitting results is higher (0.99438), we recommend that the 96.7% ANIm value could act as a threshold in delineating *Micromonospora* species.

To evaluate the impact of threshold selection, we varied the ANI cutoff from 95.0% to 97.5%. At the universal lower bound (95.0%), the maximum ANI between strain HUAS LYJ1^T^ and its closest relative (95.05%) exceeded the threshold, which would imply conspecificity. However, at any threshold ≥95.5% (including our proposed genus-specific threshold of 96.7%), the strain falls below the cutoff and is recognized as a novel species. As the 95% threshold is known to be suboptimal for *Micromonospora* (as supported by our pairwise analysis of all type strains), we advocate a data-driven threshold of 96.7%. Under this recommended threshold, the novel species assignment is robust.

The present study focused solely on *Micromonospora*; therefore, the proposed genus-specific threshold should not be extrapolated to other genera without independent validation. Ongoing work in our laboratory is examining additional actinobacterial genera to assess whether similar adjustments are needed, but those results will be reported elsewhere.

### 3.2. Evaluation of the Taxonomic Status of Strain HUAS LYJ1^T^

Morphologically, strain HUAS LYJ1^T^ produced non-motile single spores with a smooth surface on well-developed, extensively branched substrate hyphae (Figure S2) on R2A agar medium at 28 °C after incubation for 14 days, but does not produce aerial hyphae. Chemotaxonomically, the cell wall contains *meso*-diaminopimelic acid as a diagnostic amino acid. Detailed phenotypic and chemotaxonomical characteristics were provided in the species description. These data indicated that strain HUAS LYJ1^T^ represented a member of the genus *Micromonospora* [30,31].

**Figure 2:**
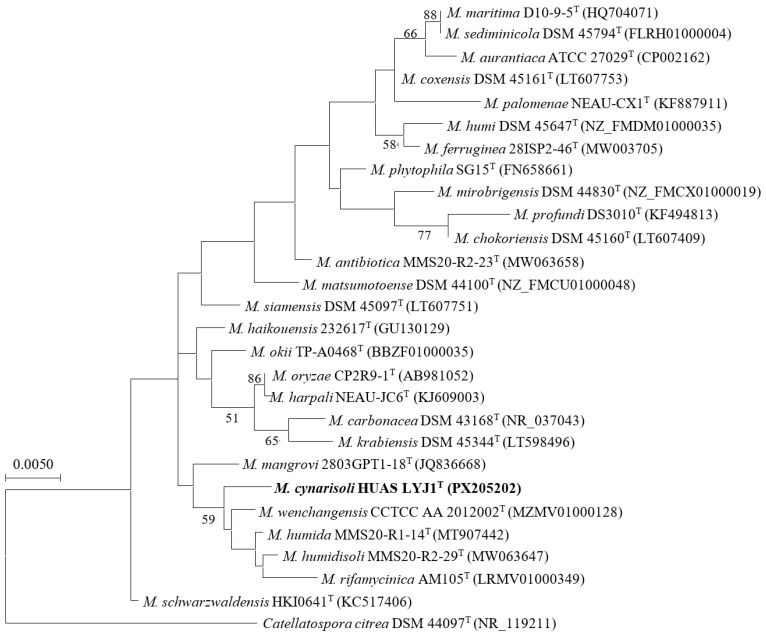
Maximum-likelihood phylogenetic tree based on 16S rRNA gene sequences (1514 bp) showing the relationship between strain HUAS LYJ1^T^ and the type strains of selected species of the genus *Micromonospora*. *Catellatospora citrea* DSM 44097^T^ was used as an outgroup. Bootstrap percentages over 50% derived from 1000 replications are shown at the nodes. Bar, 0.005 substitutions per site.

**Figure 3:**
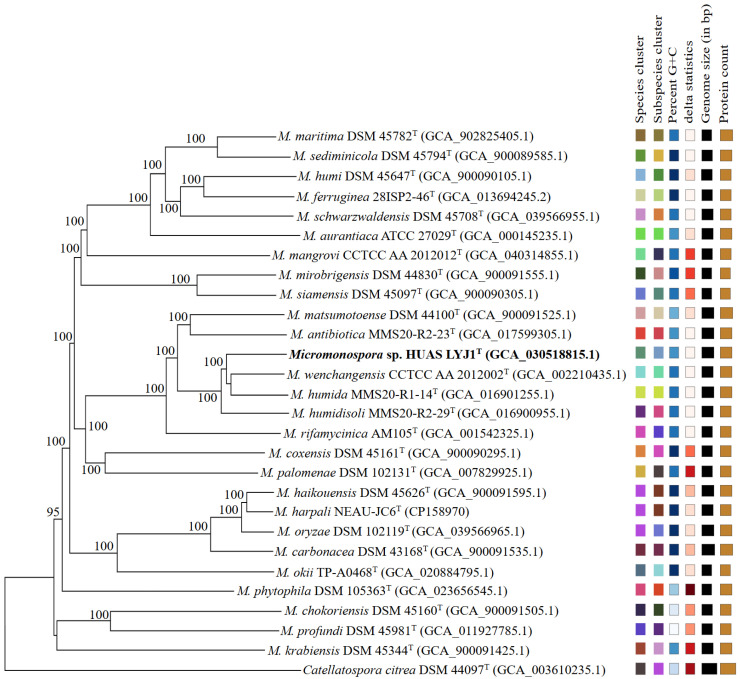
Phylogenomic tree of strain HUAS LYJ1^T^ and related type strains. The branch lengths are scaled in terms of GBDP distance formula d5. The numbers above branches are GBDP pseudo-bootstrap support values >60% from 100 replications, with an average branch support of 96.0%. The color scale on the right represents the range of pairwise GBDP d5 distances among all strains. The gradient from light to dark corresponds to increasing genetic distance (lighter = closer; darker = more distant).

At the molecular level, full-length 16S rRNA gene sequence (1514 nt) analysis showed that strain HUAS LYJ1^T^ belonged to the genus *Micromonospora* and shared highest similarity to *M. wenchangensis* CCTCC AA 2012002^T^ (99.3%). Phylogenetic trees based on 16S rRNA gene sequences demonstrated that the evolutionary neighbor of strain HUAS LYJ1^T^ was *M. wenchangensis* CCTCC AA 2012002^T^ (Figure 2, Figures S3 and S4). This result was also confirmed by a phylogenomic tree (Figure 3), suggesting that these two strains were most related to each other. However, the ANIm value between strain HUAS LYJ1^T^ and *M. wenchangensis* CCTCC AA 2012002^T^ was 95.77%, below the 96.7% cut-off point recommended above; the dDDH value between these two strains was 63.2%, also far below the 70% threshold value in delineating bacterial species [6]. These data indicated that strain HUAS LYJ1^T^ was distinct from *M. wenchangensis* CCTCC AA 2012002^T^ and should represent a new *Micromonospora* species, as was also further confirmed by the differences in phenotypic features, chemotaxonomical features and protein fingerprinting between strain HUAS LYJ1^T^ and *M. wenchangensis* CCTCC AA 2012002^T^ (Table 1, Table S3 and Figure S5). The protein fingerprints of strain HUAS LYJ1^T^ and its closest relative M. wenchangensis CCTCC AA 2012002^T^ were analyzed by MALDI-TOF MS (Figure S6). The two strains showed distinct peak patterns across the mass range of 2000–12,000 *m*/*z*. Strain HUAS LYJ1^T^ exhibited major peaks at [e.g., 3450, 5230, 7810] *m*/*z* (Figure S6a), whereas M. wenchangensis displayed prominent peaks at [e.g., 3210, 6050] *m*/*z* (Figure S6b), with clear differences in both peak positions and relative intensities. These distinct protein profiles provide additional phenotypic evidence supporting the classification of HUAS LYJ1^T^ as a novel species. In addition, the ANIm and dDDH values between strain HUAS LYJ1^T^ and all other type strains, which exhibited 16S rRNA gene sequence similarities of ≥98.7% to strain HUAS LYJ1^T^, were 86.68–95.05% and 26.5–58.9% (Table 2), respectively, much less than the 96.6% and 70% threshold values recommended in delineating *Micromonospora* species and bacterial species.

In conclusion, based on phenotypic, chemotaxonomic and genotypic data, strain HUAS LYJ1^T^ represents a novel species of the genus *Micromonospora*, for which the name *Micromonospora cynarisoli* sp. nov. is proposed.

### 3.3. Taxonomic Relationship Among M. oryzae, M. harpali and M. haikouensis

According to LPSN (List of Prokaryotic names with Standing in Nomenclature), the genus *Micromonospora* includes over 120 species with a validly published and correct name. Of all these species, *M. haikouensis* DSM 45626^T^, isolated from mangrove sediment sample, was first described as a separate genomic species by Xie et al. in 2012 [32] and approved in the same year [33]. In 2015, *M. harpali* NEAU-JC6^T^ from a beetle and *M. oryzae* DSM 102119^T^ from the root internal tissues of upland rice were reported as two different genomic species [34,35] and approved in 2015 and 2016 [36,37], respectively. However, in the present work, the clustering pattern in phylogenomic tree revealed that *M. haikouensis* DSM 45626^T^, *M. harpali* NEAU-JC6^T^ and *M.oryzae* DSM 102119^T^ should belong to the same genomic species (Figure 3), as was also confirmed by the higher ANIm (97.21–97.86%) and dDDH (73.9–79.6%) values among these three strains (Table 3). Although there were some obvious differences in the phenotypic and chemotaxonomic features among these three strains (Table 4), these differences are most likely due to adaptive selection pressures in their respective ecological niches [38], such as plant endophytes [39], rhizosphere soil [40], marine sediments [41], and so on. In the genus *Micromonospora*, numerous studies have shown that many phenotypic traits are highly plastic. For example, traits directly related to ecological adaptation, such as plant colonization ability and interaction effects, have been shown to vary significantly depending on the strain source and the host environment.

Given the environmental dependence of phenotypic traits, genome consistency is considered a more fundamental and reliable criterion for defining species in modern microbial taxonomy. Metrics based on whole-genome sequences, such as average nucleotide identity (ANI) and digital DNA-DNA hybridization (dDDH), provide stable, quantifiable measures of evolutionary relationships that are not affected by environmental factors. In this study, the relevant strains showed ANIm values of 97.21–97.86% and dDDH values of 73.9–79.6%, far above the recognized species thresholds (typically 96% ANI and 70% dDDH), providing strong evidence for classifying them as the same species. Therefore, we consider that the robust genomic homology is sufficient to support merging the strains, which exhibit limited phenotypic differences that may be driven by ecological niches, into a single taxonomic unit. Moreover, we re-examined the phenotypic data and found no single or combined trait that consistently separates the merged strains into distinct groups. The observed variations are quantitative and strain-specific, as is commonly seen within many bacterial species (e.g., in the genus *Micromonospora*).

Consequently, based on all these data and rule 42 of the International Committee on Systematics of Prokaryote Code [42], we propose that *M. harpali* [34] and *M. oryzae* [35] are later heterotypic synonyms of *M. haikouensis* [32].

## 4. Conclusions

### 4.1. The Relationship Between Average Nucleotide Identity and dDDH Values in the Genus Micromonospora

A 70% dDDH value was not equivalent to a 95–96% ANIm value, but to an approximately 96.7% ANIm value in the genus *Micromonospora*.

### 4.2. Description of Micromonospora cynarisoli sp. nov.

*Micromonospora cynarisoli* (cy.na.ri.so’li N.L. fem. n. Cynara, the scientific name of the botanical genus to which *Cynara scolymus* belongs; L. neut. n. solum, soil; N.L. gen. neut. n. cynarisoli, to indicate the isolation of the type strain from rhizosphere soil around the roots of the globe artichoke, *Cynara scolymus*)

Gram-stain-positive and strictly aerobic. Grows well on Reasoner’2A, Gauze’s synthetic No. 1 medium and ISP 3 to ISP 7. Aerial mycelium was not produced; substrate hyphae were well-developed and produced single spores with a smooth surface. A seashell pink diffusible pigment was produced on ISP 7, but no diffusible pigments were produced on Reasoner’s 2A medium, Gauze’s synthetic No. 1 medium and ISP media 2 to 6. Melanin was not produced on any of the media tested. Growth occurred at a temperature range of 10–40 °C (optimum 28 °C), a pH range of 6.0–12.0 (optimum 7.0), and a NaCl concentration range of 0–3.0% (*w*/*v*) (optimum 1.0%). Positive for milk solidification, milk peptonement, starch, nitrate reduction, gelatin liquefaction, and hydrolysis of aesculin and Tween (20, 40, 60). Negative for H_2_S production, starch hydrolysis and Tween 80 hydrolysis. In the API ZYM test, positive for cystine arylamidase, esterase (C4), esterase lipase (C8), *α*-galactosidase and *α*-glucosidase, leucine arylamidase, lipase (C14), naphtol-AS-BI-phosphohydrolase, trypsin, valine arylamidase; but negative for acid phosphatase, alkaline phosphatase, chymotrypsin, *β*-glucuronidase, *α*-mannosidase, *β*-fucosidase, *β*-galactosidase, *β*-glucosidase and *N*-acetyl-*β*-glucosaminidase. Utilizes d-galactose, d-raffinose, d-glucose as sole carbon sources, but not l-arabinose, l-rhamnose, inositol, mannitol, d-sorbitol, d-trehalose, xylitol, cellose, d-ribose, d-fructose and d-xylose. Positive for utilization of l-arginine, l-aspartic acid, creatine, l-cysteine, l-lysine, l-methionine, l-serine, and l-threonine as sole nitrogen sources, but negative for l-histidine and l-tyrosine. The major menaquinones are MK-9 (H_2_), MK-9 (H_6_), MK-10 and MK-10 (H_2_). The cell wall contains *Meso*-diaminopimelic acid, aspartic acid and glycine; whole-cell hydrolysates are glucose and xylose. The major fatty acids are *iso*-C_15:0_, *iso*-C_16:0_, C_18:0_ 10-methyl (TBSA), C_18:1_ω9c, *iso*-C_16:1_ G, C_17:0_10-methyl and Sum In Feature 9. The polar lipids are diphosphatidylglycerol (DPG), phospholipids (PLS), phosphatidyl ethanolamine (PE), phospholipids of unknown structure containing glucosamine (NPG), phosphatidylinositol (PI), phosphatidylinositol mannosides (PIM).

The type strain, strain HUAS LYJ1^T^ (=MCCC 1K08697^T^ = JCM 36306^T^), the DNA G + C content of the genome sequence, consisting of 7.52 Mbp, is 72.5%. The NCBI accession number for the full-length 16S rRNA gene sequence extracted from the whole genome and the genome sequence of strain HUAS LYJ1^T^ are PX205202 and CP130426.1, respectively.

### 4.3. Emended Description of Micromonospora haikouensis

Later heterotypic synonyms: *Micromonospora harpali* Fang et al. 2015 (Approved Lists 2015) and *Micromonospora oryzae* Kittiwongwattana et al. 2015 (Approved Lists 2016).

The description is as before [32] with the following modification. The DNA G + C content of the type-strain genome is 73.8%, its approximate size 7.58 Mbp, its GenBank accession NZ_FMCW00000000.

The type strain is DSM 45626^T^ (=232617^T^ = CCTCC AA 2011012^T^). Sequence data that support the findings of this study have been deposited in the National Center for Biotechnology Information with the primary accession code SRR32251829.

## Figures and Tables

**Table 1:** Differential features between strain HUAS LYJ1^T^ and *M. wenchangensis* CCTCC AA 2012002^T^.

Characteristics	HUAS LYJ1^T^	CCTCC AA 2012002^T^
API ZYM test:		
*α*-galactosidase, *α*-glucosidase,	+	−
*N*-acetyl-*β*-glucosaminidase	−	+
Assimilation of sole carbon sources (1.0%, *w*/*v*):		
Cellulose, d-Ribose, d-fructose, d-xylose	−	+
Starch hydrolysis	−	+
Gelatin liquefaction	+	−
Growth at/in:		
Temperature range (°C)	10–40	10–45
pH range	6.0–12.0	5.0–9.0
Major menaquinones (%):		
	MK-9 (H_2_) (6.8%)	MK-9 (H_4_) (8.9%)
	MK-9 (H_6_) (40.5%)	MK-9 (H_6_) (17.1%)
	MK-10 (13.8%)	MK-10 (H_6_) (61.1%)
	MK-10 (H_2_) (30%)	
Cell-wall amino acids	*Meso*-DAP, Asp, Gly	*Meso*-DAP, Gly
Whole-cell sugars	Glu, Xyl	Ara, Gal, Glu, Xyl
Major polar lipids:		
	DPG, NPG, PE	DPG, PE,
	PI, PIM, PLS	PI, PIM, PLS
Major cellular fatty acid composition (>5%):		
*iso*-C_15:0_	18.6	15.2
*iso*-C_16:1_ G	8.0	6.8
*iso*-C_16:0_	14.4	15.4
C_17:0_ 10-methyl	6.5	6.0
C_18:0_ 10-methyl (TBSA)	10.7	10.9
C_18:1_ ω9c	8.2	12.0
Sum In Feature 9	5.2	(3.4)

Note: +, positive; −, negative; Asp, Aspartic acid; *Meso*-DAP, *Meso*-diaminopimelic acid; Gly, Glycine; Ara, Arabinose; Gal, Galactose; Glu, Glucose; Xyl, Xylose; Sum In Feature 9, *iso*-C_17:1_ ω9c, C_16:0_ 10-methyl. All data are from this study.

**Table 2:** Comparative genotypic analysis between strain HUAS LYJ1^T^ and the closest related type strains.

Species	Strain	HUAS LYJ1^T^		
		16S rRNA Gene Sequence Similarity (%)	dDDH (%)	ANIm (%)
*M. wenchangensis*	CCTCC AA 2012002^T^	99.30	63.2	95.77
*M. humida*	MMS20-R1-14^T^	99.23	58.9	95.05
*M. humidisoli*	MMS20-R2-29^T^	99.09	57.2	94.74
*M. rifamycinica*	AM105^T^	99.03	41.2	91.42
*M. haikouensis*	DSM 45626^T^	99.03	27.0	87.11
*M. mangrovi*	CCTCC AA 2012012^T^	98.89	27.3	86.97
*M. okii*	TP-A0468^T^	98.89	26.6	86.87
*M. schwarzwaldensis*	DSM 45708^T^	98.82	26.5	86.68
*M. oryzae*	DSM 102119^T^	98.82	27.1	87.17
*M. antibiotica*	MMS20-R2-23^T^	98.82	44.8	92.34
*M. carbonacea*	DSM 43168^T^	98.75	27.4	87.25
*M. matsumotoense*	DSM 44100^T^	98.75	43.1	91.81
*M. siamensis*	DSM 45097^T^	98.75	27.2	86.99
*M. harpali*	NEAU-JC6^T^	98.75	27.0	87.12

**Table 3:** ANIm and dDDH values among *M. oryzae* DSM 102119^T^, *M. haikouensis* DSM 45626^T^ and *M. harpali* NEAU-JC6^T^.

Species	Strain	DSM 45626^T^	DSM 102119^T^	NEAU-JC6^T^
*M. haikouensis*	DSM 45626^T^		73.9%	79.6%
*M. oryzae*	DSM 102119^T^	97.21%		78.0%
*M. harpali*	NEAU-JC6^T^	97.86%	97.60%	

Note: Upper right value, dDDH; left lower value, ANIm.

**Table 4:** Differential features among *Micromonospora haikouensis* DSM 45626^T^, *Micromonospora harpali* NEU-JC6^T^ and *Micromonospora oryzae* CP2R9-1^T^.

Characteristics	1 ^b^	2 ^a^	3 ^b^
Use as sole carbon source:			
l-arabinose	–	–	+
d-fructose	–	–	+
d-mannose	–	+	+
d-xylose	–	–	+
d-mannitol	–	+	–
Fatty acids (>10%):			
	C_17:0_ (13.2)	C_16:0_ (15.4)	*iso*-C_15:0_ (17.6)
	*iso*-C_15:0_ (14.3)	*iso*-C_16:0_ (24.7)	*iso*-C_16:0_ (24.3)
	*anteiso*-C_15:0_ (12.9)	*iso*-C_17:0_ (17.8)	
	*iso*-C_16:0_ (15.8)	*anteiso*-C_17:0_ (14.8)	
	*anteiso*-C_17:0_ (10.1)		
	C_17:1_ω9c (10.9)		
Major menaquinones (>10%):			
	MK-10 (H_6_)	MK-9 (H_6_)	MK-9 (H_6_)
	MK-10 (H_4_)	MK-9 (H_4_)	MK-9 (H_4_)
Polar lipids:	DPG, PE, PIM	DPG, PE, PIM	DPG, PE, PG, PI, PIM
Whole cell sugars:	Ara, Glu, Xyl	Glu, Man, Xyl	Ara, Glu, Rib, Xyl
Cell-wall amino acid(s):	*meso*-DAP	*meso*-DAP	*meso*-DAP
			3-OH-*meso*-DAP

Note: 1, *M. haikouensis* DSM 45626^T^; 2, *M. harpali* NEU-JC6^T^; 3, *M. oryzae* CP2R9-1^T^; Glu, glucose; Xyl, xylose; Man, mannose; Ara, arabinose; Rib, ribose. a, data from [34]; b, data from [35]. +, positive; −, negative.

## Data Availability

Data will be made available on request.

## Supplementary Materials

The following supporting information can be downloaded at: https://www.mdpi.com/article/10.3390/microorganisms14050981/s1. Figure S1 The correlation between ANIb and dDDH from the 1770 pairs of *Micromonospora* species. Figure S2 Optical micrograph (a) and scanning electron micrograph (b) of strain HUAS LYJ1^T^ grown on Reasoner’ 2A medium at 28 °C after incubation for 14 days. Figure S3 Neighbor-joining phylogenetic tree based on 16S rRNA gene sequences (1514 bp) showing the relationship between selected species of the genus *Micromonospora*. Figure S4 Maximum-parsimony phylogenetic tree based on 16S rRNA gene sequences (1514 bp) showing the relationship between selected species of the genus *Micromonospora*. Figure S5 Polar lipids composition of strain HUAS LYJ1^T^ (1) and *M. wenchangensis* strain CCTCC AA 2012002^T^ (2). Figure S6 MALDI-TOF MS protein fingerprint of strain HUAS LYJ1^T^ (a) and *M. wenchangensis* strain CCTCC AA 2012002^T^ (b). Table S1 Quality analysis and GenBank assembly of genomes of *Micromonospora* species in this work (60 species). Table S2 ANI and dDDH values of 1770 pairs of type *Micromonospora* strains. Table S3 Cultural characteristics between strain HUAS LYJ1^T^ and *M. wenchangensis* strain CCTCC AA 2012002^T^.

## Abbreviations

MCCC	Marine Culture Collection of China
JCM	the Japan Collection of Microorganisms
CCTCC	China Center for Type Culture Collection
ISP	International *Streptomyces* Project
