# Supplementary material for: Re-Evaluation of the Relationship Between Average Nucleotide Identity and dDDH Values in the Genus Micromonospora, and Description of Micromonospora cynarisoli sp. nov., a Novel Actinobacterium from the Rhizosphere Soil of Cynara scolymus
Source: Microorganisms. 2026 Apr 27;14(5):981. doi: 10.3390/microorganisms14050981 (PMC13209769; doi:10.3390/microorganisms14050981)
Supplement: Supplementary file 1 [file microorganisms-14-00981-s001.zip › Supplementary materials.pdf]

**Supplementary materials**

# Re-evaluation of the relationship between Average Nucleotide Identity and dDDH values in the genus *Micromonospora*, and description of *Micromonospora cynarisoli* sp. nov., a novel actinobacterium from the rhizosphere soil of *Cynara scolymus*

Kaiqin Li[1,2†], Li Fu[2†], Peilan Long[2], Ying Qian[2], Wei Liang[1], Jian Gao[2,3]

Author affiliations:

[1] School of Computer Science and Engineering, Hunan University of Science and Technology, Xiangtan 411201, China;

[2] School of Life and Health Sciences, Hunan University of Science and technology, Xiangtan 411201, China;

[3]Key Laboratory of Ecological Remediation and Safe Utilization of Heavy Metal-Polluted Soils, College of Hunan Province, Xiangtan 411201, China

[†]These authors contributed equally to this work.

Correspondence: Kaiqin Li, likaiqin2425@hnust.edu.cn

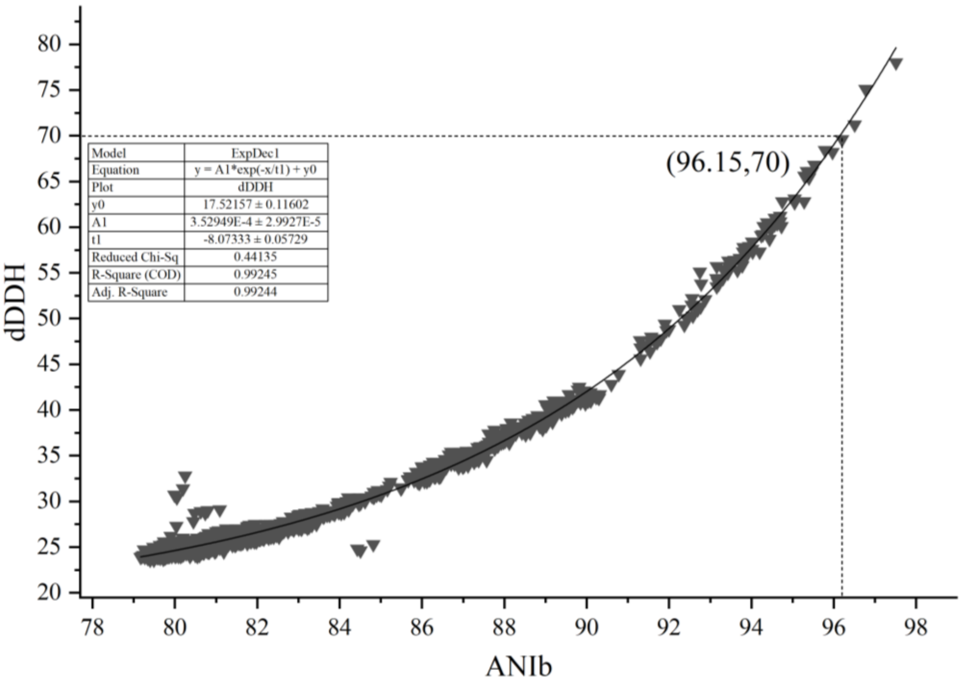


**Fig. S1** The correlation between ANIb and dDDH from the 1770 pairs of *Micromonospora* species.

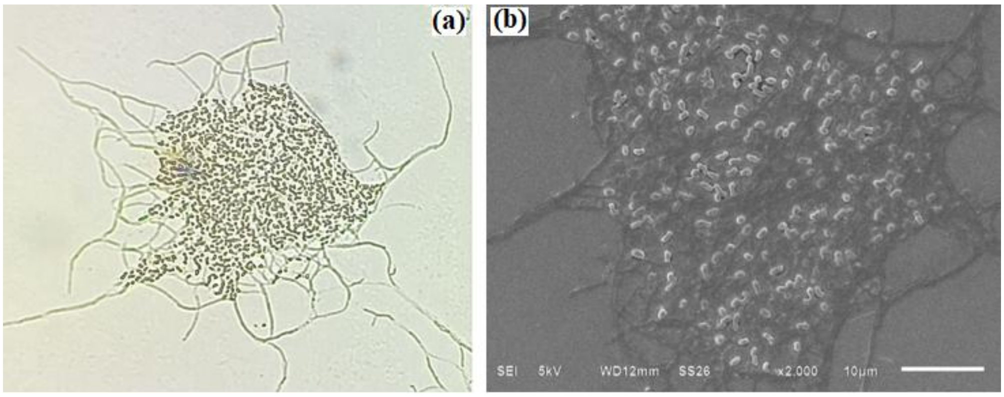


**Fig. S2** Optical micrograph (a) and scanning electron micrograph (b) of strain HUAS LYJ1$^T$ grown on Reasoner' 2A medium at 28 °C after incubation for 14 days.

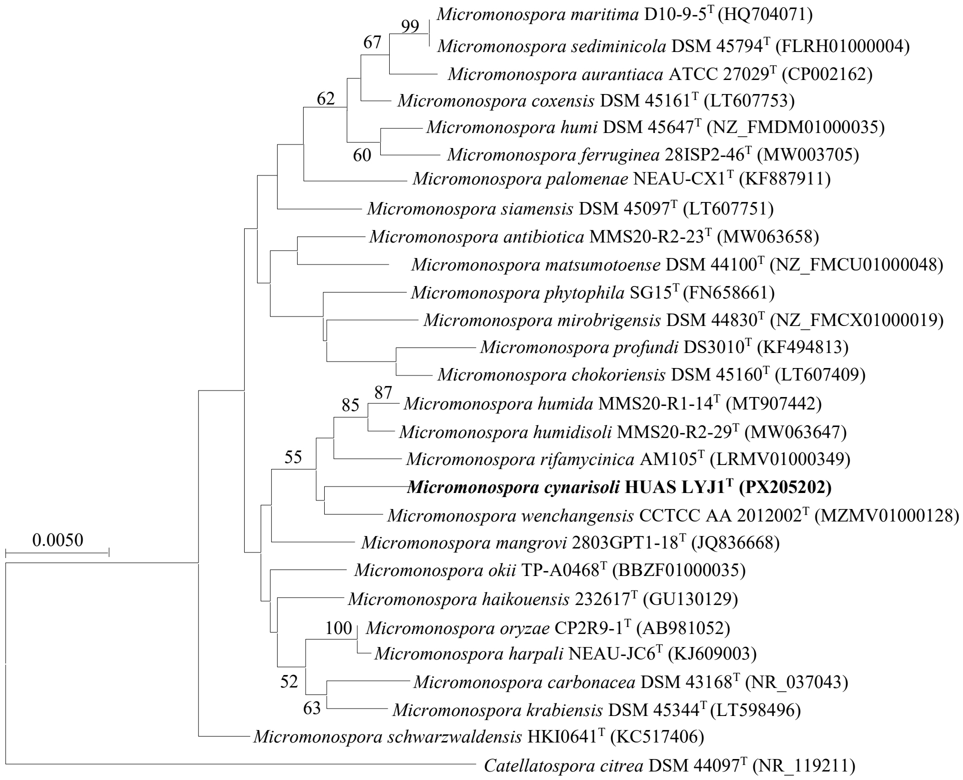


**Fig. S3** Neighbor-joining phylogenetic tree based on 16S rRNA gene sequences (1514 bp) showing the relationship between selected species of the genus *Micromonospora*. *Catellatospora citrea* DSM 44097$^{T}$ was used as an outgroup. Bootstrap percentages over 50% derived from 1000 replications are showed at the nodes. Bar, 0.005 substitutions per site.

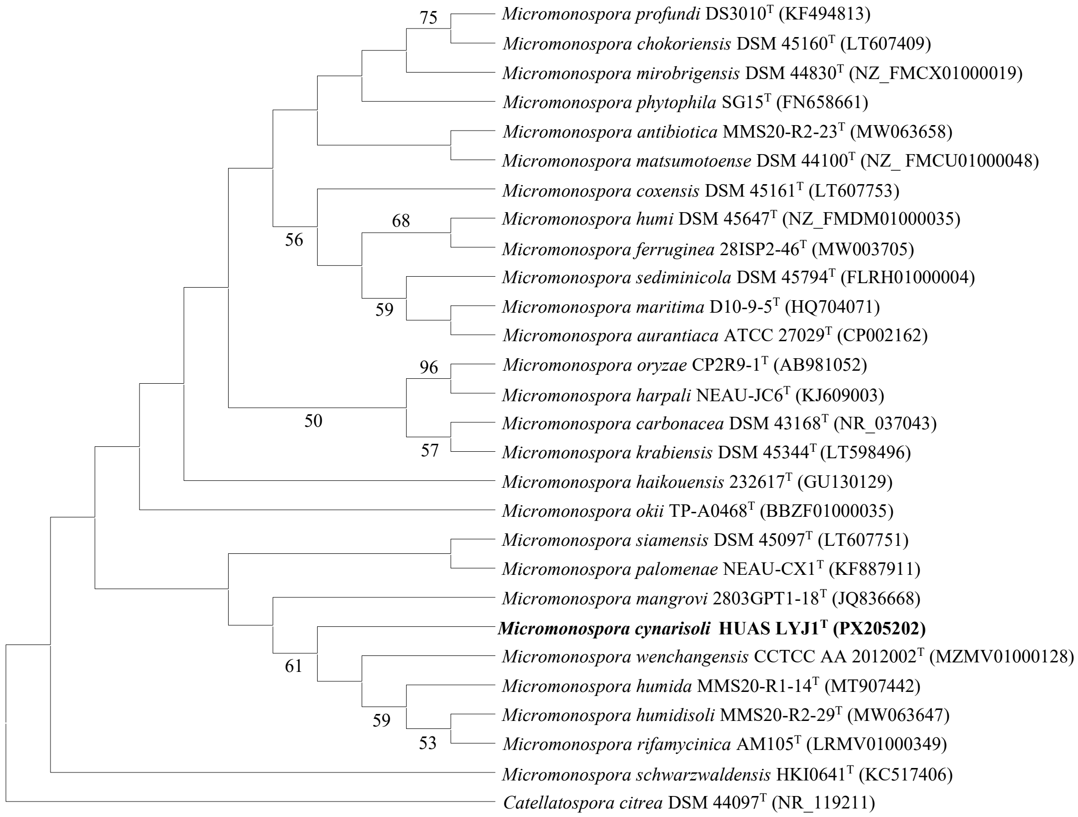


**Fig. S4** Maximum-parsimony phylogenetic tree based on 16S rRNA gene sequences (1514 bp) showing the relationship between selected species of the genus *Micromonospora*. *Catellatospora citrea* DSM 44097$^T$ was used as an outgroup. Bootstrap percentages over 50% derived from 1000 replications are showed at the nodes.

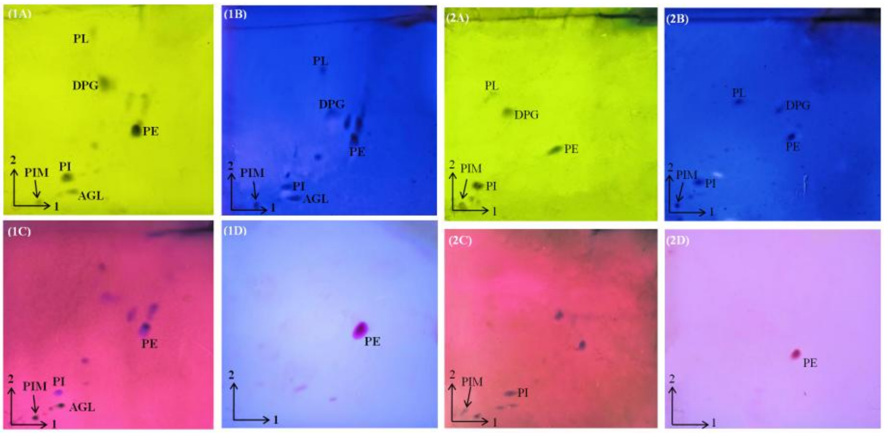


**Fig. S5** Polar lipids composition of strain HUAS LYJ1$^T$ (1) and *M. wenchangensis* strain CCTCC AA 2012002$^T$ (2).

A, Molybdenum blue reagent (for phospholipids); B, Molybdophosphoric acid (for total lipids); C, Anise aldehyde (for glycolipids); D, Ninhydrin (for aminolipids). Diphosphatidylglycerol (DPG), phospholipid (PL), phosphatidyl ethanolamine (PE), unidentified aminoglycolipids (AGL), phosphatidylinositol (PI), phosphatidylinositol mannosides (PIM).

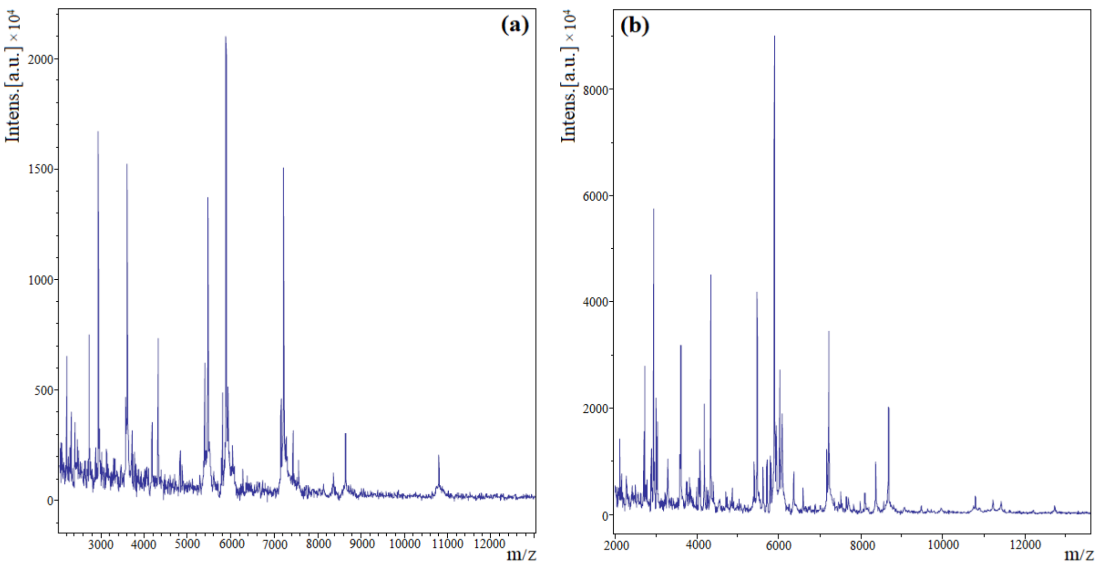


**Fig. S6** MALDI-TOF MS protein fingerprint of strain HUAS LYJ1$^{T}$ (a) and *M. wenchangensis* strain CCTCC AA 2012002$^{T}$ (b).

**Table S1** Quality analysis and GenBank assembly of genomes of *Micromonospora* species in this work (60 species).

| No. | Type strain | GenBank assembly | COM (%) | CON (%) |
|---|---|---|---|---|
| 1. | *Micromonospora acroterricola* 5R2A7 | GCA_003172955.1 | 96.09 | 3.72 |
| 2. | *Micromonospora alfalfae* MED01 | GCA_022230925.1 | 98.42 | 4.97 |
| 3. | *Micromonospora andamanensis* NBRC 109075 | GCA_016863495.1 | 94.42 | 3.25 |
| 4. | *Micromonospora antibiotica* MMS20-R2-23 | GCA_017599305.1 | 95.92 | 4.65 |
| 5. | *Micromonospora arida* LB32 | GCA_003857035.1 | 98.47 | 3.97 |
| 6. | *Micromonospora aurantiaca* ATCC 27029 | GCA_000145235.1 | 99.13 | 0.96 |
| 7. | *Micromonospora brunnea* DSM 43814 | GCA_013410185.1 | 96.04 | 3.51 |
| 8. | *Micromonospora cabrerizensis* LAH09 | GCA_022230955.1 | 98.27 | 2.03 |
| 9. | *Micromonospora chalcea* DSM 43026 | GCA_002926165.1 | 96.8 | 4.02 |
| 10. | *Micromonospora chokoriensis* DSM 45160 | GCA_900091505.1 | 96.83 | 3.26 |
| 11. | *Micromonospora coxensis* DSM 45161 | GCA_900090295.1 | 96.62 | 4.27 |
| 12. | *Micromonospora endolithica* DSM 44398 | GCA_007994185.1 | 96.41 | 4.12 |
| 13. | *Micromonospora endophytica* DSM 45430 | GCA_003236305.1 | 91.92 | 3.99 |
| 14. | *Micromonospora fiedleri* MG-37 | GCA_016774385.1 | 93.36 | 4.73 |
| 15. | *Micromonospora fluminis* A38 | GCA_902825365.1 | 98.01 | 4.74 |
| 16. | *Micromonospora foliorum* PSH25 | GCA_022229015.1 | 92.76 | 2.63 |
| 17. | *Micromonospora halophytica* DSM 43171 | GCA_900090245.1 | 94.65 | 4.06 |
| 18. | *Micromonospora harpali* NEAU-JC6 | GCA_045348645.1 | 96.12 | 4.59 |
| 19. | *Micromonospora hortensis* NIE111 | GCA_022230935.1 | 97.25 | 2.1 |
| 20. | *Micromonospora humi* DSM 45647 | GCA_900090105.1 | 97.14 | 3.1 |
| 21. | *"Micromonospora jinlongensis"* DSM 45876 | GCA_013410645.1 | 98.04 | 2.05 |
| 22. | *Micromonospora lacuserhaii* CPCC 205547 | GCA_036902735.1 | 95.57 | 2.7 |
| 23. | *Micromonospora lupini* JCM 16031 | GCA_039531855.1 | 99.33 | 1.51 |
| 24. | *Micromonospora lutea* NBRC 106530 | GCA_016863535.1 | 94.1 | 3.31 |
| 25. | *Micromonospora marina* PCU 269 | GCA_042431765.1 | 97.86 | 0.86 |
| 26. | *Micromonospora maritima* DSM 45782 | GCA_902825405.1 | 92.9 | 3.13 |
| 27. | *Micromonospora mirobrigensis* DSM 44830 | GCA_900091555.1 | 96.5 | 4 |
| 28. | *Micromonospora nigra* DSM 43818 | GCA_900091585.1 | 93.59 | 4.68 |
| 29. | *Micromonospora noduli* GUI43 | GCA_003264365.1 | 98.74 | 3.3 |
| 30. | *Micromonospora orduensis* S2509 | GCA_006228125.1 | 96.86 | 3.58 |

| | | | | |
|---|---|---|---|---|
| 31. | *Micromonospora oryzae* DSM 102119 | GCA_039566965.1 | 96.4 | 4.56 |
| 32. | *Micromonospora palomenae* DSM 102131 | GCA_007829925.1 | 96.68 | 2.67 |
| 33. | *Micromonospora palythoicola* S2-005 | GCA_036689795.1 | 93.43 | 3.61 |
| 34. | *Micromonospora parathelypteridis* DSM 103125 | GCA_014201145.1 | 97.24 | 2.82 |
| 35. | *Micromonospora phaseoli* CGMCC 4.7038 | GCA_900109115.1 | 95.81 | 2.86 |
| 36. | *Micromonospora profundi* DSM 45981 | GCA_011927785.1 | 96.86 | 4.5 |
| 37. | *Micromonospora psammae* CPCC 205556 | GCA_036902725.1 | 96.45 | 4.19 |
| 38. | *Micromonospora purpurea* DSM 43036 | GCA_014203425.1 | 97.77 | 4.95 |
| 39. | *Micromonospora purpureochromogenes* DSM 43821 | GCA_900091515.1 | 95.27 | 3.6 |
| 40. | *Micromonospora qiuiae* NBRC 106684 | GCA_016863555.1 | 90.02 | 4.23 |
| 41. | *Micromonospora rifamycinica* DSM 44983 | GCA_900090265.1 | 95.32 | 4.28 |
| 42. | *Micromonospora robiginosa* 28ISP2-46 | GCA_013694245.2 | 96 | 2.06 |
| 43. | *Micromonospora rubida* NEAU-HG-1 | GCA_009908295.1 | 94.94 | 4.74 |
| 44. | *Micromonospora saelicesensis* DSM 44871 | GCA_900091575.1 | 98.34 | 3.87 |
| 45. | *Micromonospora salmantinae* PSH03 | GCA_022230905.1 | 97.88 | 3.63 |
| 46. | *Micromonospora schwarzwaldensis* DSM 45708 | GCA_039566955.1 | 97.08 | 3.2 |
| 47. | *Micromonospora sediminicola* DSM 45794 | GCA_900089585.1 | 97.67 | 2.23 |
| 48. | *Micromonospora sediminimaris* NBRC 107745 | GCA_016863575.1 | 95.29 | 3.4 |
| 49. | *Micromonospora solifontis* PPF5-17 | GCA_010671795.1 | 91.74 | 2.88 |
| 50. | *Micromonospora sonchi* CGMCC 4.7312 | GCA_014646235.1 | 91.08 | 4.97 |
| 51. | *Micromonospora taraxaci* DSM 45885 | GCA_007830095.1 | 97.94 | 3.34 |
| 52. | *Micromonospora thermarum* HSS6-12 | GCA_012034245.1 | 93.35 | 3.71 |
| 53. | *Micromonospora trifolii* NIE79 | GCA_022229005.1 | 98.38 | 2.16 |
| 54. | *Micromonospora tulbaghiae* DSM 45142 | GCA_900091605.1 | 98.97 | 1.06 |
| 55. | *Micromonospora ureilytica* DSM 101692 | GCA_015751765.1 | 97.32 | 3.22 |
| 56. | *Micromonospora vinacea* DSM 101695 | GCA_015751785.1 | 98.71 | 2.68 |
| 57. | *Micromonospora violae* DSM 45888 | GCA_004217135.1 | 98.03 | 3.07 |
| 58. | *Micromonospora vulcania* CGMCC 4.7144 | GCA_042658605.1 | 96.49 | 4.19 |
| 59. | *Micromonospora zamorensis* DSM 45600 | GCA_900090275.1 | 98.27 | 4.28 |
| 60. | *Micromonospora zingiberis* PLAI 1-1 | GCA_004331455.1 | 92.3 | 4.26 |

Note: COM, Completeness; CON, Contamination.

**Table S3** Cultural characteristics between strain HUAS LYJ1$^T$ and *M. wenchangensis* strain CCTCC AA 2012002$^T$

| Characteristics | LYJ1$^T$ | CCTCC AA 2012002$^T$ |
|---|---|---|
| Color of aerial mycelium on G-1 | Olive Gray | Olive Gray |
| Color of substrate mycelium on G-1 | Oyster White | Alabaster Tint |
| Diffusible pigment on G-1 | None | None |
| Color of aerial mycelium on ISP 2 | None | None |
| Color of substrate mycelium on ISP 2 | None | None |
| Diffusible pigment on ISP 2 | None | None |
| Color of aerial mycelium on ISP 3 | Olive Gray | Ivy Green |
| Color of substrate mycelium on ISP 3 | String | String |
| Diffusible pigment on ISP 3 | None | None |
| Color of aerial mycelium on ISP 4 | Ivy Green | Ivy Green |
| Color of substrate mycelium on ISP 4 | Auranticus | Auranticus |
| Diffusible pigment on ISP 4 | None | None |
| Color of aerial mycelium on ISP 5 | Olive Gray | Ivy Green |
| Color of substrate mycelium on ISP 5 | Oyster White | Alabaster Tint |
| Diffusible pigment on ISP 5 | None | None |
| Color of aerial mycelium on ISP 6 | Auranticus | Auranticus |
| Color of substrate mycelium on ISP 6 | Auranticus | Auranticus |
| Diffusible pigment on ISP 6 | None | None |
| Color of aerial mycelium on ISP 7 | Seashell Pink | Olive Gray |
| Color of substrate mycelium on ISP 7 | Shell | Oyster White |
| Diffusible pigment on ISP 7 | Seashell Pink | None |
