# Supplementary material for: Re-Evaluation of the Relationship Between Average Nucleotide Identity and dDDH Values in the Genus Micromonospora, and Description of Micromonospora cynarisoli sp. nov., a Novel Actinobacterium from the Rhizosphere Soil of Cynara scolymus
Source: Microorganisms. 2026 Apr 27;14(5):981. doi: 10.3390/microorganisms14050981 (PMC13209769; doi:10.3390/microorganisms14050981)
Supplement: Supplementary file 1 [file microorganisms-14-00981-s001.zip › Table S2.pdf]

No.	Species 1	Species 2	ANIm(%)	ANIb(%)	dDDH(%)
1.	Micromonospora oryzae DSM 102119	Micromonospora harpali NEAU-JC6	97.60	97.51	78
2.	Micromonospora brunnea DSM 43814	Micromonospora purpureochromogenes DSM 43821	97.33	96.77	75.1
3.	Micromonospora noduli GUI43	Micromonospora saelicesensis DSM 44871	96.75	96.51	71.2
4.	Micromonospora chalcea DSM 43026	Micromonospora fluminis A38	96.47	96.20	69.6
5.	Micromonospora arida LB32	Micromonospora saelicesensis DSM 44871	96.33	95.79	68.4
6.	Micromonospora jinlongensis DSM 45876	Micromonospora zamorensis DSM 45600	96.33	95.97	68.2
7.	Micromonospora palomenae DSM 102131	Micromonospora brunnea DSM 43814	96.29	95.53	66.8
8.	Micromonospora noduli GUI43	Micromonospora arida LB32	96.04	95.35	66.1
9.	Micromonospora salmantinae PSH03	Micromonospora hortensis NIE111	96.00	95.47	66.1
10.	Micromonospora noduli GUI43	Micromonospora salmantinae PSH03	95.94	95.42	65.8
11.	Micromonospora palomenae DSM 102131	Micromonospora purpureochromogenes DSM 43821	96.10	95.29	65.6
12.	Micromonospora salmantinae PSH03	Micromonospora saelicesensis DSM 44871	95.90	95.40	65.4
13.	Micromonospora noduli GUI43	Micromonospora hortensis NIE111	95.55	95.05	63.1
14.	Micromonospora arida LB32	Micromonospora salmantinae PSH03	95.50	94.75	62.8
15.	Micromonospora purpurea DSM 43036	Micromonospora fluminis A38	95.55	95.28	62.8
16.	Micromonospora hortensis NIE111	Micromonospora saelicesensis DSM 44871	95.53	95.05	62.7
17.	Micromonospora chalcea DSM 43026	Micromonospora purpurea DSM 43036	95.26	94.70	61.2
18.	Micromonospora trifolii NIE79	Micromonospora salmantinae PSH03	95.21	94.57	61
19.	Micromonospora salmantinae PSH03	Micromonospora alfalfae MED01	95.21	94.64	60.7
20.	Micromonospora alfalfae MED01	Micromonospora saelicesensis DSM 44871	95.16	94.70	60.6
21.	Micromonospora arida LB32	Micromonospora hortensis NIE111	95.17	94.38	60.5
22.	Micromonospora trifolii NIE79	Micromonospora saelicesensis DSM 44871	95.13	94.63	60.4
23.	Micromonospora noduli GUI43	Micromonospora alfalfae MED01	95.16	94.57	60.3
24.	Micromonospora noduli GUI43	Micromonospora trifolii NIE79	95.11	94.42	60.3
25.	Micromonospora aurantiaca ATCC 27029	Micromonospora tulbaghiae DSM 45142	95.14	94.30	60.1
26.	Micromonospora taraxaci DSM 45885	Micromonospora chokoriensis DSM 45160	95.17	94.73	60.1
27.	Micromonospora sediminicola DSM 45794	Micromonospora maritima DSM 45782	95.19	94.47	59.7
28.	Micromonospora trifolii NIE79	Micromonospora hortensis NIE111	94.96	94.28	59.2
29.	Micromonospora arida LB32	Micromonospora alfalfae MED01	94.92	94.24	59.1
30.	Micromonospora alfalfae MED01	Micromonospora hortensis NIE111	94.88	94.44	58.7

31.	Micromonospora arida LB32	Micromonospora trifolii NIE79	94.81	94.02	58.4
32.	Micromonospora foliorum PSH25	Micromonospora saelicesensis DSM 44871	94.76	93.95	57.9
33.	Micromonospora foliorum PSH25	Micromonospora salmantinae PSH03	94.73	93.81	57.8
34.	Micromonospora noduli GUI43	Micromonospora vinacea DSM 101695	94.65	94.02	57.6
35.	Micromonospora noduli GUI43	Micromonospora foliorum PSH25	94.73	93.92	57.4
36.	Micromonospora aurantiaca ATCC 27029	Micromonospora marina PCU 269	94.74	93.80	57.3
37.	Micromonospora vinacea DSM 101695	Micromonospora saelicesensis DSM 44871	94.58	94.20	57.3
38.	Micromonospora vinacea DSM 101695	Micromonospora salmantinae PSH03	94.49	94.03	57.3
39.	Micromonospora vinacea DSM 101695	Micromonospora foliorum PSH25	94.61	93.83	57.2
40.	Micromonospora cabrerizensis LAH09	Micromonospora zamorensis DSM 45600	94.59	93.72	56.6
41.	Micromonospora trifolii NIE79	Micromonospora alfalfae MED01	94.51	93.82	56.6
42.	Micromonospora jinlongensis DSM 45876	Micromonospora cabrerizensis LAH09	94.54	93.82	56.4
43.	Micromonospora trifolii NIE79	Micromonospora foliorum PSH25	94.50	93.59	56.4
44.	Micromonospora arida LB32	Micromonospora foliorum PSH25	94.49	93.44	56.3
45.	Micromonospora vinacea DSM 101695	Micromonospora trifolii NIE79	94.34	93.78	55.8
46.	Micromonospora arida LB32	Micromonospora vinacea DSM 101695	94.29	93.46	55.7
47.	Micromonospora ureilytica DSM 101692	Micromonospora foliorum PSH25	94.38	93.16	55.7
48.	Micromonospora foliorum PSH25	Micromonospora hortensis NIE111	94.35	93.37	55.4
49.	Micromonospora vinacea DSM 101695	Micromonospora hortensis NIE111	94.21	93.66	55.3
50.	Micromonospora marina PCU 269	Micromonospora tulbaghiae DSM 45142	94.38	92.75	55.1
51.	Micromonospora ureilytica DSM 101692	Micromonospora saelicesensis DSM 44871	94.18	93.35	55.1
52.	Micromonospora noduli GUI43	Micromonospora ureilytica DSM 101692	94.16	93.38	55
53.	Micromonospora ureilytica DSM 101692	Micromonospora salmantinae PSH03	94.14	93.43	55
54.	Micromonospora ureilytica DSM 101692	Micromonospora trifolii NIE79	94.04	93.15	54.4
55.	Micromonospora ureilytica DSM 101692	Micromonospora vinacea DSM 101695	94.05	93.31	54.4

56.	Micromonospora foliorum PSH25	Micromonospora alfalfae MED01	94.09	93.18	54
57.	Micromonospora arida LB32	Micromonospora ureilytica DSM 101692	93.96	92.78	53.8
58.	Micromonospora vinacea DSM 101695	Micromonospora alfalfae MED01	93.98	93.21	53.8
59.	Micromonospora ureilytica DSM 101692	Micromonospora hortensis NIE111	93.85	93.16	53.5
60.	Micromonospora halophytica DSM 43171	Micromonospora coxensis DSM 45161	94.06	92.56	52.2
61.	Micromonospora ureilytica DSM 101692	Micromonospora alfalfae MED01	93.63	92.88	52.1
62.	Micromonospora aurantiaca ATCC 27029	Micromonospora chalcea DSM 43026	93.63	92.55	51.5
63.	Micromonospora chalcea DSM 43026	Micromonospora tulbaghiae DSM 45142	93.51	92.77	51.3
64.	Micromonospora aurantiaca ATCC 27029	Micromonospora fluminis A38	93.51	92.66	51.1
65.	Micromonospora sonchi CGMCC 4.7312	Micromonospora qiuiae NBRC 106684	93.44	92.25	51
66.	Micromonospora tulbaghiae DSM 45142	Micromonospora fluminis A38	93.52	92.70	51
67.	Micromonospora purpurea DSM 43036	Micromonospora tulbaghiae DSM 45142	93.35	92.58	50.3
68.	Micromonospora aurantiaca ATCC 27029	Micromonospora purpurea DSM 43036	93.26	92.43	50
69.	Micromonospora violae DSM 45888	Micromonospora foliorum PSH25	93.14	91.90	49.4
70.	Micromonospora robiginosa 28ISP2-46	Micromonospora humi DSM 45647	93.55	92.37	49.3
71.	Micromonospora marina PCU 269	Micromonospora fluminis A38	93.02	92.00	48.8
72.	Micromonospora chalcea DSM 43026	Micromonospora marina PCU 269	93.05	91.94	48.7
73.	Micromonospora arida LB32	Micromonospora violae DSM 45888	92.72	91.57	48
74.	Micromonospora purpurea DSM 43036	Micromonospora marina PCU 269	92.81	91.80	47.8
75.	Micromonospora violae DSM 45888	Micromonospora saelicesensis DSM 44871	92.75	91.77	47.8
76.	Micromonospora noduli GUI43	Micromonospora violae DSM 45888	92.70	91.55	47.6
77.	Micromonospora violae DSM 45888	Micromonospora salmantinae PSH03	92.69	91.64	47.6
78.	Micromonospora violae DSM 45888	Micromonospora ureilytica DSM 101692	92.70	91.30	47.6
79.	Micromonospora violae DSM 45888	Micromonospora vinacea DSM 101695	92.64	91.71	47.4
80.	Micromonospora violae DSM 45888	Micromonospora trifolii NIE79	92.57	91.46	47.1

81.	Micromonospora violae DSM 45888	Micromonospora hortensis NIE111	92.45	91.31	46.8
82.	Micromonospora violae DSM 45888	Micromonospora alfalfae MED01	92.37	91.54	46.4
83.	Micromonospora sediminimaris NBRC 107745	Micromonospora palythoicola S2-005	92.28	91.31	45.6
84.	Micromonospora robiginosa 28ISP2-46	Micromonospora schwarzwaldensis DSM 45708	91.87	90.78	43.9
85.	Micromonospora andamanensis NBRC 109075	Micromonospora sediminimaris NBRC 107745	91.47	90.60	42.8
86.	Micromonospora jinlongensis DSM 45876	Micromonospora foliorum PSH25	91.45	89.82	42.5
87.	Micromonospora foliorum PSH25	Micromonospora zamorensis DSM 45600	91.36	89.79	42.2
88.	Micromonospora foliorum PSH25	Micromonospora cabrerizensis LAH09	91.35	89.94	42.1
89.	Micromonospora arida LB32	Micromonospora zamorensis DSM 45600	91.23	89.93	41.9
90.	Micromonospora schwarzwaldensis DSM 45708	Micromonospora humi DSM 45647	91.78	90.09	41.9
91.	Micromonospora andamanensis NBRC 109075	Micromonospora palythoicola S2-005	91.24	90.18	41.7
92.	Micromonospora arida LB32	Micromonospora jinlongensis DSM 45876	91.22	90.32	41.7
93.	Micromonospora parathelypteridis DSM 103125	Micromonospora foliorum PSH25	91.20	89.58	41.7
94.	Micromonospora schwarzwaldensis DSM 45708	Micromonospora maritima DSM 45782	91.24	89.57	41.7
95.	Micromonospora jinlongensis DSM 45876	Micromonospora saelicesensis DSM 44871	91.20	90.26	41.5
96.	Micromonospora taraxaci DSM 45885	Micromonospora jinlongensis DSM 45876	91.18	90.28	41.4
97.	Micromonospora zamorensis DSM 45600	Micromonospora saelicesensis DSM 44871	91.18	90.23	41.4
98.	Micromonospora antibiotica MMS20-R2-23	Micromonospora rifamycinica DSM 44983	91.62	90.30	41.3
99.	Micromonospora noduli GUI43	Micromonospora jinlongensis DSM 45876	91.13	89.93	41.2
100.	Micromonospora robiginosa 28ISP2-46	Micromonospora maritima DSM 45782	91.18	89.68	41.2
101.	Micromonospora arida LB32	Micromonospora cabrerizensis LAH09	91.02	89.91	41.1
102.	Micromonospora jinlongensis DSM 45876	Micromonospora salmantinae PSH03	91.07	89.81	41.1
103.	Micromonospora noduli GUI43	Micromonospora zamorensis DSM 45600	91.08	89.82	41.1
104.	Micromonospora cabrerizensis LAH09	Micromonospora saelicesensis DSM 44871	91.06	90.04	41
105.	Micromonospora jinlongensis DSM 45876	Micromonospora parathelypteridis DSM 103125	91.05	89.20	41

106.	Micromonospora jinlongensis DSM 45876	Micromonospora ureilytica DSM 101692	91.03	89.47	41
107.	Micromonospora jinlongensis DSM 45876	Micromonospora vinacea DSM 101695	91.05	89.74	41
108.	Micromonospora salmantinae PSH03	Micromonospora cabrerizensis LAH09	91.02	89.77	41
109.	Micromonospora taraxaci DSM 45885	Micromonospora cabrerizensis LAH09	91.02	90.10	41
110.	Micromonospora salmantinae PSH03	Micromonospora zamorensis DSM 45600	91.02	89.50	40.9
111.	Micromonospora schwarzwaldensis DSM 45708	Micromonospora sediminicola DSM 45794	91.08	89.62	40.9
112.	Micromonospora ureilytica DSM 101692	Micromonospora cabrerizensis LAH09	90.99	89.52	40.9
113.	Micromonospora noduli GUI43	Micromonospora cabrerizensis LAH09	91.02	89.85	40.8
114.	Micromonospora ureilytica DSM 101692	Micromonospora zamorensis DSM 45600	90.98	89.33	40.8
115.	Micromonospora vinacea DSM 101695	Micromonospora cabrerizensis LAH09	91.00	89.69	40.8
116.	Micromonospora vinacea DSM 101695	Micromonospora zamorensis DSM 45600	91.00	89.61	40.8
117.	Micromonospora jinlongensis DSM 45876	Micromonospora trifolii NIE79	90.96	89.49	40.7
118.	Micromonospora taraxaci DSM 45885	Micromonospora zamorensis DSM 45600	91.03	89.97	40.7
119.	Micromonospora andamanensis NBRC 109075	Micromonospora lutea NBRC 106530	90.86	89.63	40.6
120.	Micromonospora humi DSM 45647	Micromonospora maritima DSM 45782	91.05	89.19	40.6
121.	Micromonospora parathelypteridis DSM 103125	Micromonospora cabrerizensis LAH09	90.95	89.24	40.6
122.	Micromonospora parathelypteridis DSM 103125	Micromonospora zamorensis DSM 45600	90.96	89.00	40.6
123.	Micromonospora trifolii NIE79	Micromonospora cabrerizensis LAH09	90.94	89.60	40.6
124.	Micromonospora trifolii NIE79	Micromonospora zamorensis DSM 45600	90.92	89.41	40.6
125.	Micromonospora arida LB32	Micromonospora parathelypteridis DSM 103125	90.77	89.03	40.5
126.	Micromonospora jinlongensis DSM 45876	Micromonospora chokoriensis DSM 45160	90.89	89.70	40.5
127.	Micromonospora jinlongensis DSM 45876	Micromonospora hortensis NIE111	90.85	89.48	40.4
128.	Micromonospora parathelypteridis DSM 103125	Micromonospora saelicesensis DSM 44871	90.82	89.29	40.4
129.	Micromonospora parathelypteridis DSM 103125	Micromonospora salmantinae PSH03	90.80	89.11	40.4
130.	Micromonospora robiginosa 28ISP2-46	Micromonospora sediminicola DSM 45794	90.94	89.66	40.3

131.	Micromonospora hortensis NIE111	Micromonospora zamorensis DSM 45600	90.80	89.40	40.2
132.	Micromonospora noduli GUI43	Micromonospora parathelypteridis DSM 103125	90.81	89.20	40.2
133.	Micromonospora parathelypteridis DSM 103125	Micromonospora vinacea DSM 101695	90.76	89.28	40.2
134.	Micromonospora hortensis NIE111	Micromonospora cabrerizensis LAH09	90.80	89.55	40.1
135.	Micromonospora parathelypteridis DSM 103125	Micromonospora ureilytica DSM 101692	90.77	89.05	40.1
136.	Micromonospora alfalfae MED01	Micromonospora zamorensis DSM 45600	90.72	89.42	40
137.	Micromonospora cabrerizensis LAH09	Micromonospora chokoriensis DSM 45160	90.78	89.56	40
138.	Micromonospora parathelypteridis DSM 103125	Micromonospora trifolii NIE79	90.71	89.10	40
139.	Micromonospora jinlongensis DSM 45876	Micromonospora alfalfae MED01	90.76	89.49	39.9
140.	Micromonospora zamorensis DSM 45600	Micromonospora chokoriensis DSM 45160	90.74	89.49	39.9
141.	Micromonospora taraxaci DSM 45885	Micromonospora foliorum PSH25	90.70	89.26	39.8
142.	Micromonospora alfalfae MED01	Micromonospora cabrerizensis LAH09	90.66	89.39	39.6
143.	Micromonospora lutea NBRC 106530	Micromonospora sediminimaris NBRC 107745	90.53	89.17	39.5
144.	Micromonospora parathelypteridis DSM 103125	Micromonospora hortensis NIE111	90.53	88.81	39.4
145.	Micromonospora violae DSM 45888	Micromonospora jinlongensis DSM 45876	90.54	88.95	39.3
146.	Micromonospora parathelypteridis DSM 103125	Micromonospora alfalfae MED01	90.45	88.83	39.2
147.	Micromonospora foliorum PSH25	Micromonospora chokoriensis DSM 45160	90.55	88.69	39.1
148.	Micromonospora sediminicola DSM 45794	Micromonospora humi DSM 45647	90.92	89.20	39.1
149.	Micromonospora arida LB32	Micromonospora taraxaci DSM 45885	90.43	89.05	39
150.	Micromonospora violae DSM 45888	Micromonospora cabrerizensis LAH09	90.48	88.91	39
151.	Micromonospora violae DSM 45888	Micromonospora zamorensis DSM 45600	90.44	88.77	39
152.	Micromonospora lutea NBRC 106530	Micromonospora palythoicola S2-005	90.37	88.86	38.9
153.	Micromonospora violae DSM 45888	Micromonospora parathelypteridis DSM 103125	90.39	88.59	38.9
154.	Micromonospora noduli GUI43	Micromonospora taraxaci DSM 45885	90.38	88.88	38.7
155.	Micromonospora taraxaci DSM 45885	Micromonospora saelicesensis DSM 44871	90.40	89.17	38.7

156.	Micromonospora acroterricola 5R2A7	Micromonospora vulcania CGMCC 4.7144	90.31	88.50	38.6
157.	Micromonospora taraxaci DSM 45885	Micromonospora salmantinae PSH03	90.39	89.00	38.6
158.	Micromonospora taraxaci DSM 45885	Micromonospora ureilytica DSM 101692	90.37	88.91	38.6
159.	Micromonospora taraxaci DSM 45885	Micromonospora vinacea DSM 101695	90.39	88.93	38.6
160.	Micromonospora tulbaghiae DSM 45142	Micromonospora maritima DSM 45782	90.32	88.16	38.6
161.	Micromonospora taraxaci DSM 45885	Micromonospora trifolii NIE79	90.32	88.59	38.4
162.	Micromonospora arida LB32	Micromonospora chokoriensis DSM 45160	90.23	89.02	38.3
163.	Micromonospora aurantiaca ATCC 27029	Micromonospora maritima DSM 45782	90.36	88.33	38.2
164.	Micromonospora salmantinae PSH03	Micromonospora chokoriensis DSM 45160	90.22	88.68	38.2
165.	Micromonospora taraxaci DSM 45885	Micromonospora hortensis NIE111	90.26	88.86	38.2
166.	Micromonospora taraxaci DSM 45885	Micromonospora parathelypteridis DSM 103125	90.26	88.53	38.1
167.	Micromonospora vinacea DSM 101695	Micromonospora chokoriensis DSM 45160	90.23	88.69	38.1
168.	Micromonospora chalcea DSM 43026	Micromonospora maritima DSM 45782	90.28	88.06	38
169.	Micromonospora chokoriensis DSM 45160	Micromonospora saelicesensis DSM 44871	90.18	88.93	37.9
170.	Micromonospora fluminis A38	Micromonospora maritima DSM 45782	90.25	88.11	37.9
171.	Micromonospora noduli GUI43	Micromonospora chokoriensis DSM 45160	90.18	88.65	37.9
172.	Micromonospora taraxaci DSM 45885	Micromonospora alfalfae MED01	90.21	88.66	37.9
173.	Micromonospora ureilytica DSM 101692	Micromonospora chokoriensis DSM 45160	90.16	88.58	37.9
174.	Micromonospora violae DSM 45888	Micromonospora taraxaci DSM 45885	90.13	88.50	37.9
175.	Micromonospora orduensis S2509	Micromonospora foliorum PSH25	90.03	87.76	37.8
176.	Micromonospora purpurea DSM 43036	Micromonospora maritima DSM 45782	90.23	88.12	37.8
177.	Micromonospora trifolii NIE79	Micromonospora chokoriensis DSM 45160	90.09	88.43	37.8
178.	Micromonospora acroterricola 5R2A7	Micromonospora orduensis S2509	89.99	88.06	37.6
179.	Micromonospora hortensis NIE111	Micromonospora chokoriensis DSM 45160	90.04	88.43	37.5
180.	Micromonospora violae DSM 45888	Micromonospora chokoriensis DSM 45160	90.02	88.63	37.5

181.	Micromonospora marina PCU 269	Micromonospora maritima DSM 45782	90.11	87.62	37.4
182.	Micromonospora parathelypteridis DSM 103125	Micromonospora chokoriensis DSM 45160	90.08	87.86	37.4
183.	Micromonospora sediminicola DSM 45794	Micromonospora tulbaghiae DSM 45142	90.09	88.28	37.4
184.	Micromonospora alfalfae MED01	Micromonospora chokoriensis DSM 45160	89.99	88.52	37.3
185.	Micromonospora orduensis S2509	Micromonospora jinlongensis DSM 45876	89.79	87.92	37.2
186.	Micromonospora acroterricola 5R2A7	Micromonospora parathelypteridis DSM 103125	89.82	88.04	37
187.	Micromonospora orduensis S2509	Micromonospora lupini JCM 16031	89.76	87.90	37
188.	Micromonospora acroterricola 5R2A7	Micromonospora foliorum PSH25	89.87	87.92	36.9
189.	Micromonospora arida LB32	Micromonospora orduensis S2509	89.72	88.05	36.9
190.	Micromonospora orduensis S2509	Micromonospora cabrerizensis LAH09	89.75	87.71	36.9
191.	Micromonospora orduensis S2509	Micromonospora zamorensis DSM 45600	89.72	87.94	36.9
192.	Micromonospora sediminicola DSM 45794	Micromonospora fluminis A38	89.99	88.16	36.8
193.	Micromonospora orduensis S2509	Micromonospora vinacea DSM 101695	89.71	87.88	36.7
194.	Micromonospora acroterricola 5R2A7	Micromonospora jinlongensis DSM 45876	89.69	87.92	36.6
195.	Micromonospora acroterricola 5R2A7	Micromonospora lupini JCM 16031	89.68	87.76	36.6
196.	Micromonospora aurantiaca ATCC 27029	Micromonospora sediminicola DSM 45794	90.04	88.11	36.6
197.	Micromonospora noduli GUI43	Micromonospora orduensis S2509	89.63	87.87	36.6
198.	Micromonospora orduensis S2509	Micromonospora saelicesensis DSM 44871	89.66	88.11	36.6
199.	Micromonospora orduensis S2509	Micromonospora ureilytica DSM 101692	89.63	87.76	36.6
200.	Micromonospora zingiberis PLAI 1-1	Micromonospora fiedleri MG-37	89.78	88.02	36.6
201.	Micromonospora acroterricola 5R2A7	Micromonospora zamorensis DSM 45600	89.64	87.89	36.5
202.	Micromonospora chalcea DSM 43026	Micromonospora sediminicola DSM 45794	89.96	87.98	36.5
203.	Micromonospora orduensis S2509	Micromonospora parathelypteridis DSM 103125	89.63	87.72	36.5
204.	Micromonospora orduensis S2509	Micromonospora trifolii NIE79	89.58	87.63	36.5
205.	Micromonospora acroterricola 5R2A7	Micromonospora cabrerizensis LAH09	89.67	87.93	36.4

206.	Micromonospora orduensis S2509	Micromonospora salmantinae PSH03	89.58	87.74	36.4
207.	Micromonospora purpurea DSM 43036	Micromonospora sediminicola DSM 45794	89.95	88.12	36.4
208.	Micromonospora acroterricola 5R2A7	Micromonospora salmantinae PSH03	89.50	87.60	36.2
209.	Micromonospora schwarzwaldensis DSM 45708	Micromonospora tulbaghiae DSM 45142	89.67	87.80	36.2
210.	Micromonospora acroterricola 5R2A7	Micromonospora arida LB32	89.48	87.70	36.1
211.	Micromonospora acroterricola 5R2A7	Micromonospora saelicesensis DSM 44871	89.52	87.83	36.1
212.	Micromonospora acroterricola 5R2A7	Micromonospora vinacea DSM 101695	89.51	87.85	36.1
213.	Micromonospora orduensis S2509	Micromonospora alfalfae MED01	89.48	87.78	36.1
214.	Micromonospora orduensis S2509	Micromonospora hortensis NIE111	89.42	87.56	36.1
215.	Micromonospora acroterricola 5R2A7	Micromonospora noduli GUI43	89.49	87.78	36
216.	Micromonospora foliorum PSH25	Micromonospora lupini JCM 16031	89.51	87.45	36
217.	Micromonospora robiginosa 28ISP2-46	Micromonospora tulbaghiae DSM 45142	89.58	87.69	36
218.	Micromonospora violae DSM 45888	Micromonospora orduensis S2509	89.46	87.42	36
219.	Micromonospora acroterricola 5R2A7	Micromonospora ureilytica DSM 101692	89.45	87.35	35.9
220.	Micromonospora humi DSM 45647	Micromonospora tulbaghiae DSM 45142	89.52	87.76	35.8
221.	Micromonospora marina PCU 269	Micromonospora sediminicola DSM 45794	89.80	87.77	35.8
222.	Micromonospora acroterricola 5R2A7	Micromonospora trifolii NIE79	89.41	87.58	35.7
223.	Micromonospora orduensis S2509	Micromonospora taraxaci DSM 45885	89.40	87.62	35.7
224.	Micromonospora schwarzwaldensis DSM 45708	Micromonospora fluminis A38	89.57	87.42	35.6
225.	Micromonospora acroterricola 5R2A7	Micromonospora hortensis NIE111	89.29	87.39	35.5
226.	Micromonospora orduensis S2509	Micromonospora chokoriensis DSM 45160	89.34	87.29	35.5
227.	Micromonospora purpurea DSM 43036	Micromonospora schwarzwaldensis DSM 45708	89.52	87.09	35.5
228.	Micromonospora robiginosa 28ISP2-46	Micromonospora fluminis A38	89.51	87.55	35.5
229.	Micromonospora chalcea DSM 43026	Micromonospora robiginosa 28ISP2-46	89.52	87.49	35.4
230.	Micromonospora endophytica DSM 45430	Micromonospora sonchi CGMCC 4.7312	89.31	86.95	35.4

231.	Micromonospora foliorum PSH25	Micromonospora vulcania CGMCC 4.7144	89.29	86.71	35.4
232.	Micromonospora orduensis S2509	Micromonospora vulcania CGMCC 4.7144	89.24	86.79	35.4
233.	Micromonospora acroterricola 5R2A7	Micromonospora violae DSM 45888	89.26	87.20	35.3
234.	Micromonospora chalcea DSM 43026	Micromonospora schwarzwaldensis DSM 45708	89.50	87.22	35.3
235.	Micromonospora robiginosa 28ISP2-46	Micromonospora purpurea DSM 43036	89.48	87.45	35.3
236.	Micromonospora acroterricola 5R2A7	Micromonospora alfalfae MED01	89.23	87.40	35.2
237.	Micromonospora aurantiaca ATCC 27029	Micromonospora robiginosa 28ISP2-46	89.49	87.43	35.2
238.	Micromonospora aurantiaca ATCC 27029	Micromonospora schwarzwaldensis DSM 45708	89.54	87.51	35.2
239.	Micromonospora humi DSM 45647	Micromonospora fluminis A38	89.54	87.33	35.2
240.	Micromonospora aurantiaca ATCC 27029	Micromonospora humi DSM 45647	89.50	87.38	35.1
241.	Micromonospora chalcea DSM 43026	Micromonospora humi DSM 45647	89.55	87.33	35.1
242.	Micromonospora endophytica DSM 45430	Micromonospora qiuiae NBRC 106684	89.19	86.66	35.1
243.	Micromonospora jinlongensis DSM 45876	Micromonospora lupini JCM 16031	89.22	87.43	35.1
244.	Micromonospora purpurea DSM 43036	Micromonospora humi DSM 45647	89.49	87.39	35.1
245.	Micromonospora acroterricola 5R2A7	Micromonospora taraxaci DSM 45885	89.14	87.24	35
246.	Micromonospora parathelypteridis DSM 103125	Micromonospora vulcania CGMCC 4.7144	89.12	87.07	35
247.	Micromonospora ureilytica DSM 101692	Micromonospora lupini JCM 16031	89.18	87.08	35
248.	Micromonospora vinacea DSM 101695	Micromonospora lupini JCM 16031	89.24	87.18	35
249.	Micromonospora cabrerizensis LAH09	Micromonospora lupini JCM 16031	89.25	87.16	34.9
250.	Micromonospora lupini JCM 16031	Micromonospora saelicesensis DSM 44871	89.19	87.41	34.9
251.	Micromonospora lupini JCM 16031	Micromonospora zamorensis DSM 45600	89.17	87.15	34.9
252.	Micromonospora noduli GUI43	Micromonospora lupini JCM 16031	89.21	87.12	34.9
253.	Micromonospora parathelypteridis DSM 103125	Micromonospora lupini JCM 16031	89.13	86.94	34.9
254.	Micromonospora salmantinae PSH03	Micromonospora lupini JCM 16031	89.19	87.03	34.9
255.	Micromonospora acroterricola 5R2A7	Micromonospora chokoriensis DSM 45160	89.10	86.96	34.8

256.	Micromonospora arida LB32	Micromonospora lupini JCM 16031	89.17	86.92	34.8
257.	Micromonospora acroterricola 5R2A7	Micromonospora profundi DSM 45981	89.00	86.82	34.7
258.	Micromonospora marina PCU 269	Micromonospora humi DSM 45647	89.37	86.88	34.7
259.	Micromonospora trifolii NIE79	Micromonospora lupini JCM 16031	89.12	87.05	34.7
260.	Micromonospora cabrerizensis LAH09	Micromonospora vulcania CGMCC 4.7144	88.95	86.56	34.6
261.	Micromonospora jinlongensis DSM 45876	Micromonospora vulcania CGMCC 4.7144	89.00	86.60	34.6
262.	Micromonospora profundi DSM 45981	Micromonospora lupini JCM 16031	89.10	86.87	34.6
263.	Micromonospora robiginosa 28ISP2-46	Micromonospora marina PCU 269	89.32	87.25	34.6
264.	Micromonospora schwarzwaldensis DSM 45708	Micromonospora marina PCU 269	89.34	87.33	34.6
265.	Micromonospora brunnea DSM 43814	Micromonospora psammae CPCC 205556	89.40	87.38	34.5
266.	Micromonospora orduensis S2509	Micromonospora profundi DSM 45981	88.89	86.67	34.5
267.	Micromonospora palomenae DSM 102131	Micromonospora psammae CPCC 205556	89.43	87.57	34.5
268.	Micromonospora vulcania CGMCC 4.7144	Micromonospora zamorensis DSM 45600	88.97	86.58	34.5
269.	Micromonospora hortensis NIE111	Micromonospora lupini JCM 16031	89.07	86.98	34.4
270.	Micromonospora alfalfae MED01	Micromonospora lupini JCM 16031	89.00	87.00	34.3
271.	Micromonospora arida LB32	Micromonospora vulcania CGMCC 4.7144	88.89	86.27	34.3
272.	Micromonospora lupini JCM 16031	Micromonospora vulcania CGMCC 4.7144	88.97	86.72	34.3
273.	Micromonospora psammae CPCC 205556	Micromonospora purpureochromogenes DSM 43821	89.32	87.27	34.3
274.	Micromonospora salmantinae PSH03	Micromonospora vulcania CGMCC 4.7144	88.91	86.38	34.3
275.	Micromonospora vinacea DSM 101695	Micromonospora vulcania CGMCC 4.7144	88.86	86.71	34.1
276.	Micromonospora violae DSM 45888	Micromonospora lupini JCM 16031	88.91	86.62	34.1
277.	Micromonospora vulcania CGMCC 4.7144	Micromonospora saelicesensis DSM 44871	88.92	86.79	34.1
278.	Micromonospora noduli GUI43	Micromonospora vulcania CGMCC 4.7144	88.87	86.57	34
279.	Micromonospora ureilytica DSM 101692	Micromonospora vulcania CGMCC 4.7144	88.83	86.20	34
280.	Micromonospora brunnea DSM 43814	Micromonospora lacuserhaii CPCC 205547	89.25	87.12	33.9

281.	Micromonospora palomenae DSM 102131	Micromonospora coxensis DSM 45161	89.31	87.03	33.9
282.	Micromonospora trifolii NIE79	Micromonospora vulcania CGMCC 4.7144	88.80	86.51	33.9
283.	Micromonospora alfalfae MED01	Micromonospora vulcania CGMCC 4.7144	88.70	86.41	33.8
284.	Micromonospora lacuserhaii CPCC 205547	Micromonospora purpureochromogenes DSM 43821	89.14	86.99	33.8
285.	Micromonospora palomenae DSM 102131	Micromonospora halophytica DSM 43171	89.23	86.04	33.8
286.	Micromonospora palomenae DSM 102131	Micromonospora lacuserhaii CPCC 205547	89.14	87.00	33.8
287.	Micromonospora profundi DSM 45981	Micromonospora foliorum PSH25	88.68	86.00	33.8
288.	Micromonospora rubida NEAU-HG-1	Micromonospora harpali NEAU-JC6	89.22	86.25	33.8
289.	Micromonospora rubida NEAU-HG-1	Micromonospora oryzae DSM 102119	89.24	86.19	33.8
290.	Micromonospora taraxaci DSM 45885	Micromonospora lupini JCM 16031	88.80	86.52	33.8
291.	Micromonospora brunnea DSM 43814	Micromonospora coxensis DSM 45161	89.26	86.89	33.7
292.	Micromonospora lupini JCM 16031	Micromonospora chokoriensis DSM 45160	88.78	86.59	33.7
293.	Micromonospora brunnea DSM 43814	Micromonospora halophytica DSM 43171	89.19	86.02	33.6
294.	Micromonospora hortensis NIE111	Micromonospora vulcania CGMCC 4.7144	88.74	86.13	33.6
295.	Micromonospora violae DSM 45888	Micromonospora vulcania CGMCC 4.7144	88.72	86.36	33.6
296.	Micromonospora psammae CPCC 205556	Micromonospora lacuserhaii CPCC 205547	88.87	86.48	33.5
297.	Micromonospora coxensis DSM 45161	Micromonospora purpureochromogenes DSM 43821	89.16	86.51	33.4
298.	Micromonospora halophytica DSM 43171	Micromonospora purpureochromogenes DSM 43821	89.11	86.04	33.4
299.	Micromonospora profundi DSM 45981	Micromonospora jinlongensis DSM 45876	88.53	86.42	33.3
300.	Micromonospora profundi DSM 45981	Micromonospora parathelypteridis DSM 103125	88.44	85.97	33.2
301.	Micromonospora taraxaci DSM 45885	Micromonospora vulcania CGMCC 4.7144	88.55	85.86	33.2
302.	Micromonospora profundi DSM 45981	Micromonospora cabrerizensis LAH09	88.46	86.16	33.1
303.	Micromonospora profundi DSM 45981	Micromonospora zamorensis DSM 45600	88.47	86.41	33.1
304.	Micromonospora vulcania CGMCC 4.7144	Micromonospora chokoriensis DSM 45160	88.54	85.90	33.1
305.	Micromonospora arida LB32	Micromonospora profundi DSM 45981	88.43	86.00	33

306.	Micromonospora profundi DSM 45981	Micromonospora saelicesensis DSM 44871	88.44	86.46	32.8
307.	Micromonospora profundi DSM 45981	Micromonospora vinacea DSM 101695	88.38	86.31	32.8
308.	MMicromonospora solifontis PPF5-17	Micromonospora maritima DSM 45782	86.21	80.25	32.8
309.	Micromonospora noduli GUI43	Micromonospora profundi DSM 45981	88.41	86.30	32.7
310.	Micromonospora palythoicola S2-005	Micromonospora phaseoli CGMCC 4.7038	88.31	86.43	32.7
311.	Micromonospora profundi DSM 45981	Micromonospora salmantinae PSH03	88.37	85.78	32.7
312.	Micromonospora profundi DSM 45981	Micromonospora ureilytica DSM 101692	88.33	86.08	32.7
313.	Micromonospora profundi DSM 45981	Micromonospora vulcania CGMCC 4.7144	88.42	85.71	32.7
314.	Micromonospora psammae CPCC 205556	Micromonospora coxensis DSM 45161	88.70	86.41	32.7
315.	Micromonospora sediminimaris NBRC 107745	Micromonospora phaseoli CGMCC 4.7038	88.31	86.33	32.7
316.	Micromonospora profundi DSM 45981	Micromonospora trifolii NIE79	88.31	86.05	32.6
317.	Micromonospora psammae CPCC 205556	Micromonospora halophytica DSM 43171	88.61	85.84	32.5
318.	Micromonospora andamanensis NBRC 109075	Micromonospora phaseoli CGMCC 4.7038	88.23	86.19	32.4
319.	Micromonospora profundi DSM 45981	Micromonospora alfalfae MED01	88.24	86.17	32.4
320.	Micromonospora profundi DSM 45981	Micromonospora hortensis NIE111	88.25	85.67	32.4
321.	Micromonospora violae DSM 45888	Micromonospora profundi DSM 45981	88.22	85.96	32.4
322.	Micromonospora profundi DSM 45981	Micromonospora chokoriensis DSM 45160	88.17	85.81	32.3
323.	Micromonospora zingiberis PLAI 1-1	Micromonospora sonchi CGMCC 4.7312	88.36	85.67	32.3
324.	Micromonospora lutea NBRC 106530	Micromonospora phaseoli CGMCC 4.7038	88.14	86.12	32.2
325.	Micromonospora taraxaci DSM 45885	Micromonospora profundi DSM 45981	88.16	86.06	32.2
326.	Micromonospora zingiberis PLAI 1-1	Micromonospora qiuiae NBRC 106684	88.30	85.24	32.1
327.	MMicromonospora solifontis PPF5-17	Micromonospora sediminicola DSM 45794	88.34	85.90	32.1
328.	Micromonospora lacuserhaii CPCC 205547	Micromonospora coxensis DSM 45161	88.54	85.94	32
329.	Micromonospora lacuserhaii CPCC 205547	Micromonospora halophytica DSM 43171	88.49	85.21	31.7
330.	MMicromonospora solifontis PPF5-17	Micromonospora schwarzwaldensis DSM 45708	88.14	85.51	31.5

331.	MMicromonospora solifontis PPF5-17	Micromonospora humi DSM 45647	85.89	80.20	31.4
332.	MMicromonospora solifontis PPF5-17	Micromonospora robiginosa 28ISP2-46	88.12	85.49	31.4
333.	Micromonospora endophytica DSM 45430	Micromonospora zingiberis PLAI 1-1	88.03	85.16	31.3
334.	Micromonospora sonchi CGMCC 4.7312	Micromonospora phaseoli CGMCC 4.7038	88.00	84.93	31.2
335.	Micromonospora qiuiae NBRC 106684	Micromonospora phaseoli CGMCC 4.7038	87.83	84.83	31
336.	Micromonospora zingiberis PLAI 1-1	Micromonospora phaseoli CGMCC 4.7038	87.83	84.97	30.8
337.	Micromonospora chalcea DSM 43026	MMicromonospora solifontis PPF5-17	87.85	84.90	30.7
338.	MMicromonospora solifontis PPF5-17	Micromonospora fluminis A38	85.94	79.99	30.7
339.	MMicromonospora solifontis PPF5-17	Micromonospora tulbaghiae DSM 45142	87.78	85.01	30.7
340.	Micromonospora aurantiaca ATCC 27029	MMicromonospora solifontis PPF5-17	87.77	84.78	30.6
341.	Micromonospora sonchi CGMCC 4.7312	Micromonospora fiedleri MG-37	87.79	84.82	30.6
342.	Micromonospora sonchi CGMCC 4.7312	Micromonospora sediminimaris NBRC 107745	87.67	84.84	30.5
343.	MMicromonospora solifontis PPF5-17	Micromonospora purpurea DSM 43036	87.75	84.82	30.5
344.	Micromonospora endophytica DSM 45430	Micromonospora phaseoli CGMCC 4.7038	87.64	84.47	30.4
345.	Micromonospora fiedleri MG-37	Micromonospora qiuiae NBRC 106684	87.76	84.21	30.4
346.	Micromonospora qiuiae NBRC 106684	Micromonospora sediminimaris NBRC 107745	87.63	84.67	30.4
347.	Micromonospora sonchi CGMCC 4.7312	Micromonospora palythoicola S2-005	87.62	84.52	30.4
348.	MMicromonospora solifontis PPF5-17	Micromonospora marina PCU 269	85.82	80.05	30.4
349.	Micromonospora endophytica DSM 45430	Micromonospora fiedleri MG-37	87.73	84.61	30.2
350.	Micromonospora qiuiae NBRC 106684	Micromonospora palythoicola S2-005	87.51	84.38	30.1
351.	Micromonospora andamanensis NBRC 109075	Micromonospora qiuiae NBRC 106684	87.40	84.18	30
352.	Micromonospora sonchi CGMCC 4.7312	Micromonospora andamanensis NBRC 109075	87.46	84.43	30
353.	Micromonospora zingiberis PLAI 1-1	Micromonospora palythoicola S2-005	87.55	84.62	30
354.	Micromonospora brunnea DSM 43814	Micromonospora rifamycinica DSM 44983	87.70	83.98	29.9
355.	Micromonospora lacuserhaii CPCC 205547	Micromonospora rifamycinica DSM 44983	87.70	84.29	29.9

356.	Micromonospora antibiotica MMS20-R2-23	Micromonospora lacuserhaii CPCC 205547	87.63	84.26	29.8
357.	Micromonospora endophytica DSM 45430	Micromonospora andamanensis NBRC 109075	87.34	84.20	29.8
358.	Micromonospora palomenae DSM 102131	Micromonospora rifamycinica DSM 44983	87.67	84.04	29.8
359.	Micromonospora psammae CPCC 205556	Micromonospora rifamycinica DSM 44983	87.58	83.97	29.8
360.	Micromonospora sonchi CGMCC 4.7312	Micromonospora lutea NBRC 106530	87.44	84.51	29.8
361.	Micromonospora zingiberis PLAI 1-1	Micromonospora sediminimaris NBRC 107745	87.44	84.48	29.8
362.	Micromonospora endophytica DSM 45430	Micromonospora palythoicola S2-005	87.44	84.32	29.7
363.	Micromonospora endophytica DSM 45430	Micromonospora sediminimaris NBRC 107745	87.37	84.21	29.7
364.	Micromonospora zingiberis PLAI 1-1	Micromonospora andamanensis NBRC 109075	87.43	84.47	29.7
365.	Micromonospora rifamycinica DSM 44983	Micromonospora purpureochromogenes DSM 43821	87.61	83.99	29.6
366.	Micromonospora brunnea DSM 43814	Micromonospora antibiotica MMS20-R2-23	87.67	83.94	29.5
367.	Micromonospora fiedleri MG-37	Micromonospora phaseoli CGMCC 4.7038	87.32	84.21	29.5
368.	Micromonospora lutea NBRC 106530	Micromonospora qiuiae NBRC 106684	87.30	84.17	29.5
369.	Micromonospora antibiotica MMS20-R2-23	Micromonospora psammae CPCC 205556	87.48	84.01	29.4
370.	Micromonospora antibiotica MMS20-R2-23	Micromonospora purpureochromogenes DSM 43821	87.57	83.99	29.4
371.	Micromonospora palomenae DSM 102131	Micromonospora antibiotica MMS20-R2-23	87.65	84.01	29.4
372.	Micromonospora endophytica DSM 45430	Micromonospora lutea NBRC 106530	87.17	83.98	29.3
373.	Micromonospora zingiberis PLAI 1-1	Micromonospora lutea NBRC 106530	87.26	84.11	29.3
374.	Micromonospora rifamycinica DSM 44983	Micromonospora coxensis DSM 45161	87.67	83.59	29.2
375.	Micromonospora fiedleri MG-37	Micromonospora palythoicola S2-005	87.20	84.05	29.1
376.	Micromonospora palomenae DSM 102131	MMicromonospora solifontis PPF5-17	87.59	84.22	29.1
377.	MMicromonospora solifontis PPF5-17	Micromonospora brunnea DSM 43814	86.36	81.09	29.1
378.	Micromonospora antibiotica MMS20-R2-23	Micromonospora coxensis DSM 45161	87.52	83.70	29
379.	MMicromonospora solifontis PPF5-17	Micromonospora lacuserhaii CPCC 205547	86.18	80.80	29
380.	MMicromonospora solifontis PPF5-17	Micromonospora purpureochromogenes DSM 43821	87.49	83.95	29

381.	Micromonospora halophytica DSM 43171	Micromonospora rifamycinica DSM 44983	87.43	83.51	28.9
382.	MMicromonospora solifontis PPF5-17	Micromonospora mirobrigensis DSM 44830	86.00	80.63	28.9
383.	Micromonospora antibiotica MMS20-R2-23	Micromonospora halophytica DSM 43171	87.44	83.33	28.8
384.	Micromonospora fiedleri MG-37	Micromonospora sediminimaris NBRC 107745	87.10	83.83	28.8
385.	Micromonospora lacuserhaii CPCC 205547	Micromonospora mirobrigensis DSM 44830	87.56	84.11	28.8
386.	Micromonospora palomenae DSM 102131	Micromonospora mirobrigensis DSM 44830	87.52	83.98	28.8
387.	Micromonospora brunnea DSM 43814	Micromonospora mirobrigensis DSM 44830	87.54	83.84	28.7
388.	Micromonospora endolithica DSM 44398	Micromonospora thermarum HSS6-12	87.15	84.04	28.7
389.	Micromonospora fiedleri MG-37	Micromonospora andamanensis NBRC 109075	87.13	83.68	28.7
390.	Micromonospora fiedleri MG-37	Micromonospora lutea NBRC 106530	87.13	83.72	28.7
391.	Micromonospora mirobrigensis DSM 44830	Micromonospora maritima DSM 45782	87.39	83.17	28.7
392.	MMicromonospora solifontis PPF5-17	Micromonospora coxensis DSM 45161	86.33	80.74	28.7
393.	MMicromonospora solifontis PPF5-17	Micromonospora halophytica DSM 43171	86.23	80.48	28.7
394.	Micromonospora coxensis DSM 45161	Micromonospora maritima DSM 45782	87.36	83.30	28.6
395.	Micromonospora palomenae DSM 102131	Micromonospora maritima DSM 45782	87.27	83.04	28.6
396.	Micromonospora purpureochromogenes DSM 43821	Micromonospora mirobrigensis DSM 44830	87.46	83.88	28.6
397.	MMicromonospora solifontis PPF5-17	Micromonospora psammae CPCC 205556	87.42	83.74	28.6
398.	Micromonospora brunnea DSM 43814	Micromonospora maritima DSM 45782	87.27	83.00	28.5
399.	Micromonospora coxensis DSM 45161	Micromonospora mirobrigensis DSM 44830	87.55	83.76	28.5
400.	Micromonospora rubida NEAU-HG-1	Micromonospora brunnea DSM 43814	87.41	83.70	28.5
401.	Micromonospora psammae CPCC 205556	Micromonospora mirobrigensis DSM 44830	87.43	83.63	28.4
402.	Micromonospora rubida NEAU-HG-1	Micromonospora psammae CPCC 205556	87.29	83.49	28.4
403.	Micromonospora palomenae DSM 102131	Micromonospora rubida NEAU-HG-1	87.47	83.61	28.3
404.	Micromonospora psammae CPCC 205556	Micromonospora maritima DSM 45782	87.26	82.82	28.3
405.	Micromonospora purpureochromogenes DSM 43821	Micromonospora maritima DSM 45782	87.20	82.92	28.3

406.	Micromonospora rubida NEAU-HG-1	Micromonospora coxensis DSM 45161	87.33	83.53	28.3
407.	Micromonospora rubida NEAU-HG-1	Micromonospora purpureochromogenes DSM 43821	87.37	83.51	28.3
408.	Micromonospora halophytica DSM 43171	Micromonospora maritima DSM 45782	87.20	82.98	28.2
409.	Micromonospora halophytica DSM 43171	Micromonospora mirobrigensis DSM 44830	87.42	83.24	28.2
410.	Micromonospora harpali NEAU-JC6	Micromonospora coxensis DSM 45161	87.34	83.52	28.2
411.	Micromonospora lacuserhaii CPCC 205547	Micromonospora maritima DSM 45782	87.21	83.01	28.2
412.	Micromonospora rubida NEAU-HG-1	Micromonospora lacuserhaii CPCC 205547	87.30	83.29	28.2
413.	Micromonospora acroterricola 5R2A7	Micromonospora brunnea DSM 43814	87.06	82.96	28.1
414.	Micromonospora oryzae DSM 102119	Micromonospora coxensis DSM 45161	87.38	83.51	28.1
415.	Micromonospora acroterricola 5R2A7	Micromonospora palomenae DSM 102131	87.04	82.91	28
416.	Micromonospora acroterricola 5R2A7	Micromonospora purpureochromogenes DSM 43821	86.99	83.03	28
417.	Micromonospora harpali NEAU-JC6	Micromonospora halophytica DSM 43171	87.22	83.32	28
418.	Micromonospora brunnea DSM 43814	Micromonospora harpali NEAU-JC6	87.29	83.30	27.9
419.	Micromonospora brunnea DSM 43814	Micromonospora oryzae DSM 102119	87.31	83.32	27.9
420.	Micromonospora palomenae DSM 102131	Micromonospora harpali NEAU-JC6	87.32	83.19	27.9
421.	Micromonospora palomenae DSM 102131	Micromonospora oryzae DSM 102119	87.30	83.20	27.9
422.	Micromonospora rubida NEAU-HG-1	Micromonospora halophytica DSM 43171	87.24	83.02	27.9
423.	Micromonospora harpali NEAU-JC6	Micromonospora purpureochromogenes DSM 43821	87.22	83.26	27.8
424.	Micromonospora lacuserhaii CPCC 205547	Micromonospora harpali NEAU-JC6	87.06	83.02	27.8
425.	Micromonospora oryzae DSM 102119	Micromonospora halophytica DSM 43171	87.17	83.20	27.8
426.	Micromonospora rubida NEAU-HG-1	MMicromonospora solifontis PPF5-17	87.11	83.02	27.8
427.	MMicromonospora solifontis PPF5-17	Micromonospora harpali NEAU-JC6	86.12	80.45	27.8
428.	MMicromonospora solifontis PPF5-17	Micromonospora rifamycinica DSM 44983	86.90	82.63	27.8
429.	Micromonospora lacuserhaii CPCC 205547	Micromonospora oryzae DSM 102119	87.09	83.01	27.7
430.	Micromonospora oryzae DSM 102119	Micromonospora purpureochromogenes DSM 43821	87.23	83.22	27.7

431.	Micromonospora psammae CPCC 205556	Micromonospora harpali NEAU-JC6	87.12	83.23	27.7
432.	Micromonospora acroterricola 5R2A7	Micromonospora lacuserhaii CPCC 205547	86.90	82.63	27.6
433.	Micromonospora acroterricola 5R2A7	Micromonospora psammae CPCC 205556	86.93	82.58	27.6
434.	Micromonospora psammae CPCC 205556	Micromonospora oryzae DSM 102119	87.12	83.14	27.6
435.	Micromonospora rubida NEAU-HG-1	Micromonospora mirobrigensis DSM 44830	87.03	82.80	27.6
436.	MMicromonospora solifontis PPF5-17	Micromonospora oryzae DSM 102119	87.00	82.94	27.6
437.	Micromonospora acroterricola 5R2A7	Micromonospora coxensis DSM 45161	86.82	82.49	27.5
438.	Micromonospora acroterricola 5R2A7	Micromonospora rubida NEAU-HG-1	86.79	82.22	27.5
439.	Micromonospora harpali NEAU-JC6	Micromonospora maritima DSM 45782	86.92	82.51	27.5
440.	Micromonospora oryzae DSM 102119	Micromonospora maritima DSM 45782	86.97	82.58	27.5
441.	Micromonospora palomenae DSM 102131	Micromonospora thermarum HSS6-12	87.05	83.30	27.5
442.	Micromonospora rifamycinica DSM 44983	Micromonospora maritima DSM 45782	86.84	81.95	27.5
443.	Micromonospora rifamycinica DSM 44983	Micromonospora mirobrigensis DSM 44830	87.00	82.46	27.5
444.	Micromonospora robiginosa 28ISP2-46	Micromonospora coxensis DSM 45161	86.95	83.16	27.5
445.	Micromonospora rubida NEAU-HG-1	Micromonospora maritima DSM 45782	86.93	82.33	27.5
446.	Micromonospora sediminicola DSM 45794	Micromonospora coxensis DSM 45161	87.05	83.33	27.5
447.	Micromonospora sediminicola DSM 45794	Micromonospora mirobrigensis DSM 44830	87.15	83.44	27.5
448.	Micromonospora antibiotica MMS20-R2-23	Micromonospora maritima DSM 45782	86.84	81.82	27.4
449.	Micromonospora antibiotica MMS20-R2-23	Micromonospora mirobrigensis DSM 44830	86.99	82.33	27.4
450.	Micromonospora humi DSM 45647	Micromonospora mirobrigensis DSM 44830	87.02	83.18	27.4
451.	Micromonospora palomenae DSM 102131	Micromonospora robiginosa 28ISP2-46	87.01	82.61	27.4
452.	Micromonospora palomenae DSM 102131	Micromonospora sediminicola DSM 45794	87.02	83.33	27.4
453.	Micromonospora robiginosa 28ISP2-46	Micromonospora mirobrigensis DSM 44830	87.06	83.25	27.4
454.	Micromonospora schwarzwaldensis DSM 45708	Micromonospora coxensis DSM 45161	86.92	82.95	27.4
455.	Micromonospora schwarzwaldensis DSM 45708	Micromonospora mirobrigensis DSM 44830	87.01	83.05	27.4

456.	Micromonospora thermarum HSS6-12	Micromonospora brunnea DSM 43814	87.00	83.28	27.4
457.	Micromonospora acroterricola 5R2A7	Micromonospora endolithica DSM 44398	86.69	82.51	27.3
458.	Micromonospora acroterricola 5R2A7	Micromonospora halophytica DSM 43171	86.70	82.50	27.3
459.	Micromonospora antibiotica MMS20-R2-23	Micromonospora harpali NEAU-JC6	87.06	82.31	27.3
460.	Micromonospora brunnea DSM 43814	Micromonospora foliorum PSH25	86.73	81.82	27.3
461.	Micromonospora brunnea DSM 43814	Micromonospora humi DSM 45647	86.95	82.99	27.3
462.	Micromonospora brunnea DSM 43814	Micromonospora robiginosa 28ISP2-46	86.99	83.07	27.3
463.	Micromonospora brunnea DSM 43814	Micromonospora sediminicola DSM 45794	87.06	82.83	27.3
464.	Micromonospora brunnea DSM 43814	Micromonospora vulcania CGMCC 4.7144	86.84	82.81	27.3
465.	Micromonospora endolithica DSM 44398	Micromonospora brunnea DSM 43814	86.95	82.68	27.3
466.	Micromonospora harpali NEAU-JC6	Micromonospora mirobrigensis DSM 44830	86.96	82.71	27.3
467.	Micromonospora harpali NEAU-JC6	Micromonospora rifamycinica DSM 44983	87.14	82.62	27.3
468.	Micromonospora lacuserhaii CPCC 205547	Micromonospora humi DSM 45647	86.91	83.16	27.3
469.	Micromonospora oryzae DSM 102119	Micromonospora mirobrigensis DSM 44830	86.96	82.75	27.3
470.	Micromonospora oryzae DSM 102119	Micromonospora rifamycinica DSM 44983	87.10	82.45	27.3
471.	Micromonospora palomenae DSM 102131	Micromonospora endolithica DSM 44398	86.91	82.94	27.3
472.	Micromonospora palomenae DSM 102131	Micromonospora foliorum PSH25	86.75	81.78	27.3
473.	Micromonospora palomenae DSM 102131	Micromonospora humi DSM 45647	86.92	82.69	27.3
474.	Micromonospora palomenae DSM 102131	Micromonospora schwarzwaldensis DSM 45708	86.95	83.06	27.3
475.	Micromonospora psammae CPCC 205556	Micromonospora sediminicola DSM 45794	87.01	83.25	27.3
476.	Micromonospora thermarum HSS6-12	Micromonospora purpureochromogenes DSM 43821	87.01	83.19	27.3
477.	MMicromonospora solifontis PPF5-17	Micromonospora antibiotica MMS20-R2-23	85.84	80.04	27.3
478.	Micromonospora brunnea DSM 43814	Micromonospora schwarzwaldensis DSM 45708	86.95	83.00	27.2
479.	Micromonospora foliorum PSH25	Micromonospora purpureochromogenes DSM 43821	86.72	81.76	27.2
480.	Micromonospora humi DSM 45647	Micromonospora purpureochromogenes DSM 43821	86.85	83.05	27.2

481.	Micromonospora palomenae DSM 102131	Micromonospora vulcania CGMCC 4.7144	86.80	82.81	27.2
482.	Micromonospora psammae CPCC 205556	Micromonospora schwarzwaldensis DSM 45708	86.88	82.88	27.2
483.	Micromonospora robiginosa 28ISP2-46	Micromonospora lacuserhaii CPCC 205547	86.94	83.07	27.2
484.	Micromonospora robiginosa 28ISP2-46	Micromonospora psammae CPCC 205556	86.91	82.97	27.2
485.	Micromonospora sediminicola DSM 45794	Micromonospora halophytica DSM 43171	86.82	82.90	27.2
486.	Micromonospora sediminicola DSM 45794	Micromonospora purpureochromogenes DSM 43821	86.98	83.26	27.2
487.	Micromonospora thermarum HSS6-12	Micromonospora coxensis DSM 45161	86.96	82.93	27.2
488.	Micromonospora thermarum HSS6-12	Micromonospora psammae CPCC 205556	86.85	82.99	27.2
489.	Micromonospora acroterricola 5R2A7	Micromonospora harpali NEAU-JC6	86.60	81.93	27.1
490.	Micromonospora acroterricola 5R2A7	Micromonospora oryzae DSM 102119	86.67	81.92	27.1
491.	Micromonospora acroterricola 5R2A7	Micromonospora thermarum HSS6-12	86.61	82.33	27.1
492.	Micromonospora antibiotica MMS20-R2-23	Micromonospora oryzae DSM 102119	87.01	82.27	27.1
493.	Micromonospora coxensis DSM 45161	Micromonospora fluminis A38	86.86	82.51	27.1
494.	Micromonospora endolithica DSM 44398	Micromonospora purpureochromogenes DSM 43821	86.89	82.81	27.1
495.	Micromonospora lacuserhaii CPCC 205547	Micromonospora sediminicola DSM 45794	86.99	83.22	27.1
496.	Micromonospora mirobrigensis DSM 44830	Micromonospora fluminis A38	86.84	82.74	27.1
497.	Micromonospora orduensis S2509	Micromonospora brunnea DSM 43814	86.79	82.31	27.1
498.	Micromonospora orduensis S2509	Micromonospora palomenae DSM 102131	86.80	82.26	27.1
499.	Micromonospora psammae CPCC 205556	Micromonospora humi DSM 45647	86.87	82.83	27.1
500.	Micromonospora robiginosa 28ISP2-46	Micromonospora halophytica DSM 43171	86.78	82.62	27.1
501.	Micromonospora robiginosa 28ISP2-46	Micromonospora purpureochromogenes DSM 43821	86.90	82.55	27.1
502.	Micromonospora schwarzwaldensis DSM 45708	Micromonospora halophytica DSM 43171	86.78	82.60	27.1
503.	Micromonospora vulcania CGMCC 4.7144	Micromonospora purpureochromogenes DSM 43821	86.78	82.76	27.1
504.	MMicromonospora solifontis PPF5-17	Micromonospora thermarum HSS6-12	86.88	82.72	27.1
505.	Micromonospora acroterricola 5R2A7	MMicromonospora solifontis PPF5-17	86.64	82.43	27

506.	Micromonospora brunnea DSM 43814	Micromonospora fluminis A38	86.82	82.26	27
507.	Micromonospora endolithica DSM 44398	Micromonospora coxensis DSM 45161	86.89	82.40	27
508.	Micromonospora endolithica DSM 44398	Micromonospora foliorum PSH25	86.48	81.58	27
509.	Micromonospora endolithica DSM 44398	Micromonospora psammae CPCC 205556	86.90	83.08	27
510.	Micromonospora foliorum PSH25	Micromonospora lacuserhaii CPCC 205547	86.64	82.39	27
511.	Micromonospora foliorum PSH25	Micromonospora psammae CPCC 205556	86.62	81.50	27
512.	Micromonospora lacuserhaii CPCC 205547	Micromonospora schwarzwaldensis DSM 45708	86.83	83.07	27
513.	Micromonospora orduensis S2509	Micromonospora purpureochromogenes DSM 43821	86.72	82.13	27
514.	Micromonospora palomenae DSM 102131	Micromonospora fluminis A38	86.83	82.21	27
515.	Micromonospora palomenae DSM 102131	Micromonospora tulbaghiae DSM 45142	86.81	82.46	27
516.	Micromonospora rubida NEAU-HG-1	Micromonospora foliorum PSH25	86.59	81.25	27
517.	Micromonospora schwarzwaldensis DSM 45708	Micromonospora purpureochromogenes DSM 43821	86.87	83.01	27
518.	Micromonospora thermarum HSS6-12	Micromonospora lacuserhaii CPCC 205547	86.74	82.86	27
519.	Micromonospora acroterricola 5R2A7	Micromonospora rifamycinica DSM 44983	86.46	81.45	26.9
520.	Micromonospora aurantiaca ATCC 27029	Micromonospora coxensis DSM 45161	86.80	82.64	26.9
521.	Micromonospora aurantiaca ATCC 27029	Micromonospora mirobrigensis DSM 44830	86.85	82.53	26.9
522.	Micromonospora brunnea DSM 43814	Micromonospora tulbaghiae DSM 45142	86.76	82.53	26.9
523.	Micromonospora chalcea DSM 43026	Micromonospora coxensis DSM 45161	86.84	82.63	26.9
524.	Micromonospora chalcea DSM 43026	Micromonospora mirobrigensis DSM 44830	86.85	82.66	26.9
525.	Micromonospora chalcea DSM 43026	Micromonospora palomenae DSM 102131	86.82	82.66	26.9
526.	Micromonospora coxensis DSM 45161	Micromonospora tulbaghiae DSM 45142	86.89	82.47	26.9
527.	Micromonospora endolithica DSM 44398	Micromonospora maritima DSM 45782	86.65	81.94	26.9
528.	Micromonospora endolithica DSM 44398	Micromonospora rubida NEAU-HG-1	86.69	81.69	26.9
529.	Micromonospora foliorum PSH25	Micromonospora coxensis DSM 45161	86.60	81.39	26.9
530.	Micromonospora humi DSM 45647	Micromonospora coxensis DSM 45161	87.01	82.63	26.9

531.	Micromonospora psammae CPCC 205556	Micromonospora vulcania CGMCC 4.7144	86.75	82.89	26.9
532.	Micromonospora rubida NEAU-HG-1	Micromonospora antibiotica MMS20-R2-23	86.86	81.34	26.9
533.	Micromonospora thermarum HSS6-12	Micromonospora maritima DSM 45782	86.67	81.97	26.9
534.	Micromonospora acroterricola 5R2A7	Micromonospora maritima DSM 45782	86.65	81.82	26.8
535.	Micromonospora acroterricola 5R2A7	Micromonospora mirobrigensis DSM 44830	86.65	81.68	26.8
536.	Micromonospora brunnea DSM 43814	Micromonospora lupini JCM 16031	86.64	82.66	26.8
537.	Micromonospora endolithica DSM 44398	Micromonospora halophytica DSM 43171	86.74	81.87	26.8
538.	Micromonospora endolithica DSM 44398	Micromonospora lacuserhaii CPCC 205547	86.69	82.46	26.8
539.	Micromonospora endophytica DSM 45430	Micromonospora nigra DSM 43818	86.27	81.31	26.8
540.	Micromonospora foliorum PSH25	Micromonospora harpali NEAU-JC6	86.46	81.07	26.8
541.	Micromonospora harpali NEAU-JC6	Micromonospora sediminicola DSM 45794	86.68	82.55	26.8
542.	Micromonospora lacuserhaii CPCC 205547	Micromonospora tulbaghiae DSM 45142	86.69	82.53	26.8
543.	Micromonospora marina PCU 269	Micromonospora coxensis DSM 45161	86.79	82.59	26.8
544.	Micromonospora mirobrigensis DSM 44830	Micromonospora tulbaghiae DSM 45142	86.79	82.63	26.8
545.	Micromonospora orduensis S2509	Micromonospora rubida NEAU-HG-1	86.59	81.66	26.8
546.	Micromonospora purpurea DSM 43036	Micromonospora mirobrigensis DSM 44830	86.79	82.65	26.8
547.	Micromonospora purpureochromogenes DSM 43821	Micromonospora fluminis A38	86.79	82.09	26.8
548.	Micromonospora purpureochromogenes DSM 43821	Micromonospora tulbaghiae DSM 45142	86.72	82.36	26.8
549.	Micromonospora rubida NEAU-HG-1	Micromonospora rifamycinica DSM 44983	86.92	81.62	26.8
550.	Micromonospora rubida NEAU-HG-1	Micromonospora thermarum HSS6-12	86.69	82.27	26.8
551.	Micromonospora rubida NEAU-HG-1	Micromonospora vulcania CGMCC 4.7144	86.57	81.64	26.8
552.	Micromonospora thermarum HSS6-12	Micromonospora halophytica DSM 43171	86.87	82.54	26.8
553.	Micromonospora thermarum HSS6-12	Micromonospora nigra DSM 43818	86.50	82.49	26.8
554.	Micromonospora aurantiaca ATCC 27029	Micromonospora brunnea DSM 43814	86.76	82.63	26.7
555.	Micromonospora aurantiaca ATCC 27029	Micromonospora halophytica DSM 43171	86.81	82.37	26.7

556.	Micromonospora brunnea DSM 43814	Micromonospora purpurea DSM 43036	86.73	82.55	26.7
557.	Micromonospora chalcea DSM 43026	Micromonospora brunnea DSM 43814	86.77	82.57	26.7
558.	Micromonospora endolithica DSM 44398	Micromonospora nigra DSM 43818	86.58	81.70	26.7
559.	Micromonospora foliorum PSH25	Micromonospora halophytica DSM 43171	86.48	81.68	26.7
560.	Micromonospora foliorum PSH25	Micromonospora oryzae DSM 102119	86.47	81.01	26.7
561.	Micromonospora halophytica DSM 43171	Micromonospora fluminis A38	86.69	82.38	26.7
562.	Micromonospora humi DSM 45647	Micromonospora halophytica DSM 43171	86.83	82.24	26.7
563.	Micromonospora lacuserhaii CPCC 205547	Micromonospora fluminis A38	86.74	82.63	26.7
564.	Micromonospora lacuserhaii CPCC 205547	Micromonospora vulcania CGMCC 4.7144	86.67	82.60	26.7
565.	Micromonospora lupini JCM 16031	Micromonospora purpureochromogenes DSM 43821	86.60	82.58	26.7
566.	Micromonospora marina PCU 269	Micromonospora mirobrigensis DSM 44830	86.79	82.25	26.7
567.	Micromonospora orduensis S2509	Micromonospora harpali NEAU-JC6	86.47	81.54	26.7
568.	Micromonospora orduensis S2509	Micromonospora lacuserhaii CPCC 205547	86.57	82.41	26.7
569.	Micromonospora orduensis S2509	Micromonospora psammae CPCC 205556	86.61	82.75	26.7
570.	Micromonospora oryzae DSM 102119	Micromonospora sediminicola DSM 45794	86.70	82.59	26.7
571.	Micromonospora palomenae DSM 102131	Micromonospora purpurea DSM 43036	86.76	82.54	26.7
572.	Micromonospora psammae CPCC 205556	Micromonospora fluminis A38	86.73	82.59	26.7
573.	Micromonospora purpurea DSM 43036	Micromonospora coxensis DSM 45161	86.82	82.56	26.7
574.	Micromonospora schwarzwaldensis DSM 45708	Micromonospora harpali NEAU-JC6	86.71	82.13	26.7
575.	Micromonospora schwarzwaldensis DSM 45708	Micromonospora oryzae DSM 102119	86.73	82.21	26.7
576.	Micromonospora sonchi CGMCC 4.7312	Micromonospora nigra DSM 43818	86.29	81.32	26.7
577.	Micromonospora thermarum HSS6-12	Micromonospora harpali NEAU-JC6	86.69	82.09	26.7
578.	Micromonospora thermarum HSS6-12	Micromonospora oryzae DSM 102119	86.69	82.24	26.7
579.	Micromonospora acroterricola 5R2A7	Micromonospora antibiotica MMS20-R2-23	86.43	81.39	26.6
580.	Micromonospora arida LB32	Micromonospora rubida NEAU-HG-1	86.49	81.24	26.6

581.	Micromonospora aurantiaca ATCC 27029	Micromonospora palomenae DSM 102131	86.77	82.57	26.6
582.	Micromonospora aurantiaca ATCC 27029	Micromonospora purpureochromogenes DSM 43821	86.74	82.60	26.6
583.	Micromonospora brunnea DSM 43814	Micromonospora marina PCU 269	86.70	82.21	26.6
584.	Micromonospora chalcea DSM 43026	Micromonospora halophytica DSM 43171	86.69	82.22	26.6
585.	Micromonospora chalcea DSM 43026	Micromonospora psammae CPCC 205556	86.80	82.52	26.6
586.	Micromonospora chalcea DSM 43026	Micromonospora purpureochromogenes DSM 43821	86.73	82.60	26.6
587.	Micromonospora coxensis DSM 45161	Micromonospora nigra DSM 43818	86.63	82.20	26.6
588.	Micromonospora endolithica DSM 44398	Micromonospora vulcania CGMCC 4.7144	86.45	81.64	26.6
589.	Micromonospora halophytica DSM 43171	Micromonospora tulbaghiae DSM 45142	86.76	82.16	26.6
590.	Micromonospora orduensis S2509	Micromonospora coxensis DSM 45161	86.56	81.70	26.6
591.	Micromonospora orduensis S2509	Micromonospora endolithica DSM 44398	86.55	81.98	26.6
592.	Micromonospora psammae CPCC 205556	Micromonospora lupini JCM 16031	86.44	82.57	26.6
593.	Micromonospora purpurea DSM 43036	Micromonospora purpureochromogenes DSM 43821	86.66	82.50	26.6
594.	Micromonospora robiginosa 28ISP2-46	Micromonospora harpali NEAU-JC6	86.66	82.29	26.6
595.	Micromonospora robiginosa 28ISP2-46	Micromonospora oryzae DSM 102119	86.69	82.32	26.6
596.	Micromonospora rubida NEAU-HG-1	Micromonospora fluminis A38	86.46	82.03	26.6
597.	Micromonospora rubida NEAU-HG-1	Micromonospora robiginosa 28ISP2-46	86.55	82.11	26.6
598.	Micromonospora rubida NEAU-HG-1	Micromonospora sediminicola DSM 45794	86.64	82.31	26.6
599.	Micromonospora vulcania CGMCC 4.7144	Micromonospora coxensis DSM 45161	86.65	81.68	26.6
600.	Micromonospora arida LB32	Micromonospora brunnea DSM 43814	86.50	81.51	26.5
601.	Micromonospora arida LB32	Micromonospora palomenae DSM 102131	86.50	81.51	26.5
602.	Micromonospora arida LB32	Micromonospora purpureochromogenes DSM 43821	86.47	81.44	26.5
603.	Micromonospora aurantiaca ATCC 27029	Micromonospora lacuserhaii CPCC 205547	86.68	82.42	26.5
604.	Micromonospora brunnea DSM 43814	Micromonospora jinlongensis DSM 45876	86.62	81.55	26.5
605.	Micromonospora chalcea DSM 43026	Micromonospora lacuserhaii CPCC 205547	86.74	82.49	26.5

606.	Micromonospora chalcea DSM 43026	Micromonospora rubida NEAU-HG-1	86.47	81.82	26.5
607.	Micromonospora endolithica DSM 44398	Micromonospora oryzae DSM 102119	86.63	81.49	26.5
608.	Micromonospora endolithica DSM 44398	MMicromonospora solifontis PPF5-17	86.61	82.14	26.5
609.	Micromonospora foliorum PSH25	Micromonospora rifamycinica DSM 44983	86.33	80.82	26.5
610.	Micromonospora halophytica DSM 43171	Micromonospora nigra DSM 43818	86.55	82.41	26.5
611.	Micromonospora lacuserhaii CPCC 205547	Micromonospora lupini JCM 16031	86.46	82.39	26.5
612.	Micromonospora noduli GUI43	Micromonospora brunnea DSM 43814	86.52	82.37	26.5
613.	Micromonospora noduli GUI43	Micromonospora palomenae DSM 102131	86.48	82.43	26.5
614.	Micromonospora noduli GUI43	Micromonospora purpureochromogenes DSM 43821	86.53	82.22	26.5
615.	Micromonospora orduensis S2509	Micromonospora oryzae DSM 102119	86.41	81.34	26.5
616.	Micromonospora orduensis S2509	Micromonospora thermarum HSS6-12	86.36	82.38	26.5
617.	Micromonospora palomenae DSM 102131	Micromonospora lupini JCM 16031	86.57	82.58	26.5
618.	Micromonospora palomenae DSM 102131	Micromonospora marina PCU 269	86.65	82.25	26.5
619.	Micromonospora purpurea DSM 43036	Micromonospora halophytica DSM 43171	86.65	82.28	26.5
620.	Micromonospora purpurea DSM 43036	Micromonospora lacuserhaii CPCC 205547	86.65	82.52	26.5
621.	Micromonospora rubida NEAU-HG-1	Micromonospora lupini JCM 16031	86.49	81.18	26.5
622.	Micromonospora antibiotica MMS20-R2-23	Micromonospora foliorum PSH25	86.29	80.83	26.4
623.	Micromonospora arida LB32	Micromonospora psammae CPCC 205556	86.44	82.45	26.4
624.	Micromonospora aurantiaca ATCC 27029	Micromonospora psammae CPCC 205556	86.70	82.54	26.4
625.	Micromonospora brunnea DSM 43814	Micromonospora cabrerizensis LAH09	86.54	81.51	26.4
626.	Micromonospora brunnea DSM 43814	Micromonospora parathelypteridis DSM 103125	86.49	81.35	26.4
627.	Micromonospora brunnea DSM 43814	Micromonospora saelicesensis DSM 44871	86.52	81.71	26.4
628.	Micromonospora brunnea DSM 43814	Micromonospora ureilytica DSM 101692	86.52	82.12	26.4
629.	Micromonospora brunnea DSM 43814	Micromonospora vinacea DSM 101695	86.50	82.37	26.4
630.	Micromonospora endolithica DSM 44398	Micromonospora harpali NEAU-JC6	86.58	81.43	26.4

631.	Micromonospora lacuserhaii CPCC 205547	Micromonospora marina PCU 269	86.65	81.97	26.4
632.	Micromonospora lupini JCM 16031	Micromonospora oryzae DSM 102119	86.43	80.88	26.4
633.	Micromonospora marina PCU 269	Micromonospora purpureochromogenes DSM 43821	86.61	82.11	26.4
634.	Micromonospora orduensis S2509	Micromonospora halophytica DSM 43171	86.46	82.01	26.4
635.	Micromonospora psammae CPCC 205556	Micromonospora marina PCU 269	86.66	82.12	26.4
636.	Micromonospora psammae CPCC 205556	Micromonospora tulbaghiae DSM 45142	86.69	82.49	26.4
637.	Micromonospora qiuiae NBRC 106684	Micromonospora nigra DSM 43818	86.23	80.88	26.4
638.	Micromonospora robiginosa 28ISP2-46	Micromonospora rifamycinica DSM 44983	86.61	81.72	26.4
639.	Micromonospora rubida NEAU-HG-1	Micromonospora purpurea DSM 43036	86.38	81.77	26.4
640.	Micromonospora rubida NEAU-HG-1	Micromonospora salmantinae PSH03	86.37	81.00	26.4
641.	Micromonospora rubida NEAU-HG-1	Micromonospora schwarzwaldensis DSM 45708	86.45	81.94	26.4
642.	Micromonospora schwarzwaldensis DSM 45708	Micromonospora rifamycinica DSM 44983	86.65	81.60	26.4
643.	Micromonospora sediminicola DSM 45794	Micromonospora rifamycinica DSM 44983	86.67	81.62	26.4
644.	Micromonospora thermarum HSS6-12	Micromonospora foliorum PSH25	86.29	82.04	26.4
645.	Micromonospora vinacea DSM 101695	Micromonospora purpureochromogenes DSM 43821	86.49	82.24	26.4
646.	Micromonospora violae DSM 45888	Micromonospora brunnea DSM 43814	86.52	82.18	26.4
647.	Micromonospora violae DSM 45888	Micromonospora palomenae DSM 102131	86.52	82.24	26.4
648.	Micromonospora vulcania CGMCC 4.7144	Micromonospora halophytica DSM 43171	86.52	82.02	26.4
649.	Micromonospora acroterricola 5R2A7	Micromonospora nigra DSM 43818	86.21	81.39	26.3
650.	Micromonospora acroterricola 5R2A7	Micromonospora schwarzwaldensis DSM 45708	86.39	81.68	26.3
651.	Micromonospora acroterricola 5R2A7	Micromonospora sediminicola DSM 45794	86.37	81.51	26.3
652.	Micromonospora antibiotica MMS20-R2-23	Micromonospora humi DSM 45647	86.51	81.63	26.3
653.	Micromonospora antibiotica MMS20-R2-23	Micromonospora schwarzwaldensis DSM 45708	86.58	81.52	26.3
654.	Micromonospora antibiotica MMS20-R2-23	Micromonospora sediminicola DSM 45794	86.63	81.67	26.3
655.	Micromonospora arida LB32	Micromonospora coxensis DSM 45161	86.32	81.13	26.3

656.	Micromonospora arida LB32	Micromonospora lacuserhaii CPCC 205547	86.35	82.25	26.3
657.	Micromonospora aurantiaca ATCC 27029	Micromonospora rubida NEAU-HG-1	86.44	81.86	26.3
658.	Micromonospora brunnea DSM 43814	Micromonospora alfalfae MED01	86.34	82.11	26.3
659.	Micromonospora brunnea DSM 43814	Micromonospora hortensis NIE111	86.39	81.29	26.3
660.	Micromonospora brunnea DSM 43814	Micromonospora nigra DSM 43818	86.56	81.26	26.3
661.	Micromonospora brunnea DSM 43814	Micromonospora salmantinae PSH03	86.41	81.41	26.3
662.	Micromonospora brunnea DSM 43814	Micromonospora trifolii NIE79	86.38	82.29	26.3
663.	Micromonospora brunnea DSM 43814	Micromonospora zamorensis DSM 45600	86.52	81.40	26.3
664.	Micromonospora cabrerizensis LAH09	Micromonospora psammae CPCC 205556	86.50	82.39	26.3
665.	Micromonospora cabrerizensis LAH09	Micromonospora purpureochromogenes DSM 43821	86.51	81.52	26.3
666.	Micromonospora endolithica DSM 44398	Micromonospora lupini JCM 16031	86.28	81.82	26.3
667.	Micromonospora endolithica DSM 44398	Micromonospora mirobrigensis DSM 44830	86.59	82.31	26.3
668.	Micromonospora foliorum PSH25	Micromonospora maritima DSM 45782	86.31	81.08	26.3
669.	Micromonospora foliorum PSH25	Micromonospora mirobrigensis DSM 44830	86.42	81.76	26.3
670.	Micromonospora harpali NEAU-JC6	Micromonospora fluminis A38	86.53	81.94	26.3
671.	Micromonospora harpali NEAU-JC6	Micromonospora humi DSM 45647	86.67	81.93	26.3
672.	Micromonospora harpali NEAU-JC6	Micromonospora tulbaghiae DSM 45142	86.54	81.87	26.3
673.	Micromonospora humi DSM 45647	Micromonospora rifamycinica DSM 44983	86.58	81.68	26.3
674.	Micromonospora jinlongensis DSM 45876	Micromonospora purpureochromogenes DSM 43821	86.57	81.50	26.3
675.	Micromonospora lupini JCM 16031	Micromonospora coxensis DSM 45161	86.45	81.77	26.3
676.	Micromonospora lupini JCM 16031	Micromonospora harpali NEAU-JC6	86.38	80.94	26.3
677.	Micromonospora marina PCU 269	Micromonospora halophytica DSM 43171	86.66	82.35	26.3
678.	Micromonospora nigra DSM 43818	Micromonospora maritima DSM 45782	86.33	81.20	26.3
679.	Micromonospora noduli GUI43	Micromonospora rubida NEAU-HG-1	86.33	80.96	26.3
680.	Micromonospora orduensis S2509	Micromonospora maritima DSM 45782	86.41	81.46	26.3

681.	Micromonospora oryzae DSM 102119	Micromonospora fluminis A38	86.57	81.95	26.3
682.	Micromonospora oryzae DSM 102119	Micromonospora tulbaghiae DSM 45142	86.61	81.90	26.3
683.	Micromonospora oryzae DSM 102119	Micromonospora vulcania CGMCC 4.7144	86.39	81.08	26.3
684.	Micromonospora palomenae DSM 102131	Micromonospora parathelypteridis DSM 103125	86.47	81.22	26.3
685.	Micromonospora palomenae DSM 102131	Micromonospora saelicesensis DSM 44871	86.49	81.55	26.3
686.	Micromonospora palomenae DSM 102131	Micromonospora salmantinae PSH03	86.42	81.39	26.3
687.	Micromonospora palomenae DSM 102131	Micromonospora trifolii NIE79	86.43	82.34	26.3
688.	Micromonospora palomenae DSM 102131	Micromonospora ureilytica DSM 101692	86.48	82.17	26.3
689.	Micromonospora palomenae DSM 102131	Micromonospora vinacea DSM 101695	86.50	82.36	26.3
690.	Micromonospora palomenae DSM 102131	Micromonospora zamorensis DSM 45600	86.50	81.44	26.3
691.	Micromonospora purpurea DSM 43036	Micromonospora psammae CPCC 205556	86.66	82.47	26.3
692.	Micromonospora purpureochromogenes DSM 43821	Micromonospora saelicesensis DSM 44871	86.45	81.51	26.3
693.	Micromonospora robiginosa 28ISP2-46	Micromonospora antibiotica MMS20-R2-23	86.53	81.74	26.3
694.	Micromonospora rubida NEAU-HG-1	Micromonospora humi DSM 45647	86.54	81.93	26.3
695.	Micromonospora rubida NEAU-HG-1	Micromonospora saelicesensis DSM 44871	86.36	81.25	26.3
696.	Micromonospora rubida NEAU-HG-1	Micromonospora tulbaghiae DSM 45142	86.46	81.75	26.3
697.	Micromonospora rubida NEAU-HG-1	Micromonospora vinacea DSM 101695	86.36	81.06	26.3
698.	Micromonospora salmantinae PSH03	Micromonospora purpureochromogenes DSM 43821	86.37	81.37	26.3
699.	Micromonospora thermarum HSS6-12	Micromonospora mirobrigensis DSM 44830	86.54	82.27	26.3
700.	Micromonospora thermarum HSS6-12	Micromonospora vulcania CGMCC 4.7144	86.35	82.29	26.3
701.	Micromonospora ureilytica DSM 101692	Micromonospora purpureochromogenes DSM 43821	86.47	82.01	26.3
702.	Micromonospora violae DSM 45888	Micromonospora purpureochromogenes DSM 43821	86.45	82.10	26.3
703.	Micromonospora zamorensis DSM 45600	Micromonospora purpureochromogenes DSM 43821	86.48	81.42	26.3
704.	Micromonospora zingiberis PLAI 1-1	Micromonospora nigra DSM 43818	86.25	81.46	26.3
705.	MMicromonospora solifontis PPF5-17	Micromonospora vulcania CGMCC 4.7144	86.39	82.15	26.3

706.	Micromonospora acroterricola 5R2A7	Micromonospora humi DSM 45647	86.33	81.38	26.2
707.	Micromonospora acroterricola 5R2A7	Micromonospora robiginosa 28ISP2-46	86.35	81.41	26.2
708.	Micromonospora alfalfae MED01	Micromonospora purpureochromogenes DSM 43821	86.33	82.07	26.2
709.	Micromonospora arida LB32	Micromonospora halophytica DSM 43171	86.23	82.16	26.2
710.	Micromonospora aurantiaca ATCC 27029	Micromonospora harpali NEAU-JC6	86.65	82.04	26.2
711.	Micromonospora endolithica DSM 44398	Micromonospora cabrerizensis LAH09	86.28	81.29	26.2
712.	Micromonospora endolithica DSM 44398	Micromonospora rifamycinica DSM 44983	86.48	81.06	26.2
713.	Micromonospora endolithica DSM 44398	Micromonospora salmantinae PSH03	86.23	81.34	26.2
714.	Micromonospora hortensis NIE111	Micromonospora purpureochromogenes DSM 43821	86.35	81.12	26.2
715.	Micromonospora lupini JCM 16031	Micromonospora halophytica DSM 43171	86.35	81.73	26.2
716.	Micromonospora lupini JCM 16031	Micromonospora maritima DSM 45782	86.41	81.44	26.2
717.	Micromonospora nigra DSM 43818	Micromonospora phaseoli CGMCC 4.7038	86.16	81.69	26.2
718.	Micromonospora noduli GUI43	Micromonospora lacuserhaii CPCC 205547	86.36	82.13	26.2
719.	Micromonospora noduli GUI43	Micromonospora psammae CPCC 205556	86.39	82.35	26.2
720.	Micromonospora orduensis S2509	Micromonospora rifamycinica DSM 44983	86.34	81.07	26.2
721.	Micromonospora orduensis S2509	MMicromonospora solifontis PPF5-17	86.32	82.02	26.2
722.	Micromonospora oryzae DSM 102119	Micromonospora humi DSM 45647	86.66	81.95	26.2
723.	Micromonospora palomenae DSM 102131	Micromonospora alfalfae MED01	86.36	82.23	26.2
724.	Micromonospora palomenae DSM 102131	Micromonospora cabrerizensis LAH09	86.53	81.43	26.2
725.	Micromonospora palomenae DSM 102131	Micromonospora hortensis NIE111	86.40	81.18	26.2
726.	Micromonospora palomenae DSM 102131	Micromonospora jinlongensis DSM 45876	86.59	81.45	26.2
727.	Micromonospora palythoicola S2-005	Micromonospora nigra DSM 43818	86.14	81.57	26.2
728.	Micromonospora parathelypteridis DSM 103125	Micromonospora psammae CPCC 205556	86.41	82.29	26.2
729.	Micromonospora parathelypteridis DSM 103125	Micromonospora purpureochromogenes DSM 43821	86.46	81.29	26.2
730.	Micromonospora purpurea DSM 43036	Micromonospora harpali NEAU-JC6	86.49	81.72	26.2

731.	Micromonospora rifamycinica DSM 44983	Micromonospora tulbaghiae DSM 45142	86.42	81.42	26.2
732.	Micromonospora rubida NEAU-HG-1	Micromonospora hortensis NIE111	86.24	80.77	26.2
733.	Micromonospora rubida NEAU-HG-1	Micromonospora marina PCU 269	86.41	81.90	26.2
734.	Micromonospora rubida NEAU-HG-1	Micromonospora ureilytica DSM 101692	86.28	80.77	26.2
735.	Micromonospora salmantinae PSH03	Micromonospora psammae CPCC 205556	86.39	82.29	26.2
736.	Micromonospora trifolii NIE79	Micromonospora purpureochromogenes DSM 43821	86.36	82.12	26.2
737.	Micromonospora vinacea DSM 101695	Micromonospora lacuserhaii CPCC 205547	86.44	82.07	26.2
738.	Micromonospora violae DSM 45888	Micromonospora psammae CPCC 205556	86.41	82.14	26.2
739.	Micromonospora vulcania CGMCC 4.7144	Micromonospora maritima DSM 45782	86.40	81.36	26.2
740.	MMicromonospora solifontis PPF5-17	Micromonospora foliorum PSH25	85.85	79.90	26.2
741.	Micromonospora alfalfae MED01	Micromonospora psammae CPCC 205556	86.25	82.19	26.1
742.	Micromonospora arida LB32	Micromonospora endolithica DSM 44398	86.20	81.21	26.1
743.	Micromonospora brunnea DSM 43814	Micromonospora chokoriensis DSM 45160	86.49	81.20	26.1
744.	Micromonospora cabrerizensis LAH09	Micromonospora lacuserhaii CPCC 205547	86.45	82.13	26.1
745.	Micromonospora chalcea DSM 43026	Micromonospora harpali NEAU-JC6	86.52	81.63	26.1
746.	Micromonospora chalcea DSM 43026	Micromonospora oryzae DSM 102119	86.53	81.69	26.1
747.	Micromonospora chokoriensis DSM 45160	Micromonospora purpureochromogenes DSM 43821	86.42	81.14	26.1
748.	Micromonospora coxensis DSM 45161	Micromonospora saelicesensis DSM 44871	86.28	81.28	26.1
749.	Micromonospora endolithica DSM 44398	Micromonospora chokoriensis DSM 45160	86.30	81.04	26.1
750.	Micromonospora jinlongensis DSM 45876	Micromonospora psammae CPCC 205556	86.44	82.40	26.1
751.	Micromonospora lacuserhaii CPCC 205547	Micromonospora nigra DSM 43818	86.37	81.08	26.1
752.	Micromonospora noduli GUI43	Micromonospora coxensis DSM 45161	86.35	80.98	26.1
753.	Micromonospora palomenae DSM 102131	Micromonospora nigra DSM 43818	86.49	81.29	26.1
754.	Micromonospora palomenae DSM 102131	Micromonospora profundi DSM 45981	86.31	81.23	26.1
755.	Micromonospora parathelypteridis DSM 103125	Micromonospora lacuserhaii CPCC 205547	86.36	81.90	26.1

756.	Micromonospora profundi DSM 45981	Micromonospora brunnea DSM 43814	86.28	81.37	26.1
757.	Micromonospora psammae CPCC 205556	Micromonospora nigra DSM 43818	86.55	81.52	26.1
758.	Micromonospora psammae CPCC 205556	Micromonospora saelicesensis DSM 44871	86.46	82.53	26.1
759.	Micromonospora psammae CPCC 205556	Micromonospora zamorensis DSM 45600	86.39	82.34	26.1
760.	Micromonospora purpurea DSM 43036	Micromonospora oryzae DSM 102119	86.55	81.64	26.1
761.	Micromonospora purpureochromogenes DSM 43821	Micromonospora nigra DSM 43818	86.49	81.13	26.1
762.	Micromonospora rifamycinica DSM 44983	Micromonospora fluminis A38	86.37	81.17	26.1
763.	Micromonospora rubida NEAU-HG-1	Micromonospora alfalfae MED01	86.28	80.81	26.1
764.	Micromonospora rubida NEAU-HG-1	Micromonospora nigra DSM 43818	86.44	81.34	26.1
765.	Micromonospora rubida NEAU-HG-1	Micromonospora parathelypteridis DSM 103125	86.30	80.83	26.1
766.	Micromonospora rubida NEAU-HG-1	Micromonospora trifolii NIE79	86.22	80.96	26.1
767.	Micromonospora salmantinae PSH03	Micromonospora lacuserhaii CPCC 205547	86.37	82.11	26.1
768.	Micromonospora sonchi CGMCC 4.7312	Micromonospora coxensis DSM 45161	86.33	80.74	26.1
769.	Micromonospora taraxaci DSM 45885	Micromonospora brunnea DSM 43814	86.38	80.87	26.1
770.	Micromonospora thermarum HSS6-12	Micromonospora lupini JCM 16031	86.20	82.12	26.1
771.	Micromonospora ureilytica DSM 101692	Micromonospora coxensis DSM 45161	86.30	80.82	26.1
772.	Micromonospora vinacea DSM 101695	Micromonospora coxensis DSM 45161	86.34	81.82	26.1
773.	Micromonospora vinacea DSM 101695	Micromonospora psammae CPCC 205556	86.41	82.27	26.1
774.	Micromonospora violae DSM 45888	Micromonospora coxensis DSM 45161	86.33	81.53	26.1
775.	Micromonospora violae DSM 45888	Micromonospora rubida NEAU-HG-1	86.31	81.00	26.1
776.	Micromonospora vulcania CGMCC 4.7144	Micromonospora harpali NEAU-JC6	86.39	81.20	26.1
777.	Micromonospora vulcania CGMCC 4.7144	Micromonospora mirobrigensis DSM 44830	86.43	81.83	26.1
778.	Micromonospora vulcania CGMCC 4.7144	Micromonospora rifamycinica DSM 44983	86.35	80.73	26.1
779.	MMicromonospora solifontis PPF5-17	Micromonospora nigra DSM 43818	86.29	81.32	26.1
780.	Micromonospora alfalfae MED01	Micromonospora coxensis DSM 45161	86.20	81.73	26

781.	Micromonospora alfalfae MED01	Micromonospora lacuserhaii CPCC 205547	86.26	82.00	26
782.	Micromonospora andamanensis NBRC 109075	Micromonospora nigra DSM 43818	85.99	81.49	26
783.	Micromonospora antibiotica MMS20-R2-23	Micromonospora tulbaghiae DSM 45142	86.29	81.47	26
784.	Micromonospora antibiotica MMS20-R2-23	Micromonospora vulcania CGMCC 4.7144	86.29	80.69	26
785.	Micromonospora arida LB32	Micromonospora harpali NEAU-JC6	86.26	80.83	26
786.	Micromonospora arida LB32	Micromonospora oryzae DSM 102119	86.26	80.80	26
787.	Micromonospora arida LB32	Micromonospora rifamycinica DSM 44983	86.12	80.55	26
788.	Micromonospora aurantiaca ATCC 27029	Micromonospora oryzae DSM 102119	86.59	81.92	26
789.	Micromonospora chalcea DSM 43026	Micromonospora rifamycinica DSM 44983	86.37	80.99	26
790.	Micromonospora endolithica DSM 44398	Micromonospora antibiotica MMS20-R2-23	86.40	81.01	26
791.	Micromonospora endolithica DSM 44398	Micromonospora hortensis NIE111	86.17	81.21	26
792.	Micromonospora endolithica DSM 44398	Micromonospora saelicesensis DSM 44871	86.17	81.50	26
793.	Micromonospora endolithica DSM 44398	Micromonospora ureilytica DSM 101692	86.18	81.00	26
794.	Micromonospora endolithica DSM 44398	Micromonospora vinacea DSM 101695	86.25	81.71	26
795.	Micromonospora fiedleri MG-37	Micromonospora nigra DSM 43818	86.13	81.08	26
796.	Micromonospora foliorum PSH25	Micromonospora nigra DSM 43818	85.98	80.98	26
797.	Micromonospora hortensis NIE111	Micromonospora coxensis DSM 45161	86.23	80.83	26
798.	Micromonospora hortensis NIE111	Micromonospora lacuserhaii CPCC 205547	86.32	81.90	26
799.	Micromonospora hortensis NIE111	Micromonospora psammae CPCC 205556	86.28	82.14	26
800.	Micromonospora lacuserhaii CPCC 205547	Micromonospora chokoriensis DSM 45160	86.35	81.78	26
801.	Micromonospora lacuserhaii CPCC 205547	Micromonospora saelicesensis DSM 44871	86.34	82.28	26
802.	Micromonospora lupini JCM 16031	Micromonospora rifamycinica DSM 44983	86.27	80.64	26
803.	Micromonospora marina PCU 269	Micromonospora rifamycinica DSM 44983	86.40	81.25	26
804.	Micromonospora noduli GUI43	Micromonospora endolithica DSM 44398	86.21	81.20	26
805.	Micromonospora noduli GUI43	Micromonospora halophytica DSM 43171	86.25	81.73	26

806.	Micromonospora orduensis S2509	Micromonospora antibiotica MMS20-R2-23	86.29	80.97	26
807.	Micromonospora palomenae DSM 102131	Micromonospora chokoriensis DSM 45160	86.52	81.17	26
808.	Micromonospora palomenae DSM 102131	Micromonospora sonchi CGMCC 4.7312	86.36	80.99	26
809.	Micromonospora palomenae DSM 102131	Micromonospora taraxaci DSM 45885	86.43	80.90	26
810.	Micromonospora profundi DSM 45981	Micromonospora purpureochromogenes DSM 43821	86.25	81.29	26
811.	Micromonospora purpurea DSM 43036	Micromonospora rifamycinica DSM 44983	86.37	80.94	26
812.	Micromonospora salmantinae PSH03	Micromonospora coxensis DSM 45161	86.32	81.00	26
813.	Micromonospora taraxaci DSM 45885	Micromonospora purpureochromogenes DSM 43821	86.35	80.84	26
814.	Micromonospora thermarum HSS6-12	Micromonospora qiuiae NBRC 106684	86.31	81.47	26
815.	Micromonospora thermarum HSS6-12	Micromonospora rifamycinica DSM 44983	86.25	81.37	26
816.	Micromonospora thermarum HSS6-12	Micromonospora sediminicola DSM 45794	86.43	82.09	26
817.	Micromonospora thermarum HSS6-12	Micromonospora sonchi CGMCC 4.7312	86.39	81.66	26
818.	Micromonospora trifolii NIE79	Micromonospora coxensis DSM 45161	86.22	81.62	26
819.	Micromonospora trifolii NIE79	Micromonospora lacuserhaii CPCC 205547	86.35	82.07	26
820.	Micromonospora trifolii NIE79	Micromonospora psammae CPCC 205556	86.31	82.16	26
821.	Micromonospora ureilytica DSM 101692	Micromonospora lacuserhaii CPCC 205547	86.29	81.85	26
822.	Micromonospora ureilytica DSM 101692	Micromonospora psammae CPCC 205556	86.31	82.12	26
823.	Micromonospora violae DSM 45888	Micromonospora lacuserhaii CPCC 205547	86.34	81.99	26
824.	MMicromonospora solifontis PPF5-17	Micromonospora lupini JCM 16031	85.70	80.19	26
825.	Micromonospora antibiotica MMS20-R2-23	Micromonospora fluminis A38	86.36	81.14	25.9
826.	Micromonospora arida LB32	Micromonospora thermarum HSS6-12	86.05	81.92	25.9
827.	Micromonospora aurantiaca ATCC 27029	Micromonospora rifamycinica DSM 44983	86.34	81.05	25.9
828.	Micromonospora brunnea DSM 43814	Micromonospora sonchi CGMCC 4.7312	86.36	81.09	25.9
829.	Micromonospora endolithica DSM 44398	Micromonospora parathelypteridis DSM 103125	86.23	80.97	25.9
830.	Micromonospora endolithica DSM 44398	Micromonospora phaseoli CGMCC 4.7038	86.21	81.53	25.9

831.	Micromonospora endolithica DSM 44398	Micromonospora sediminicola DSM 45794	86.42	81.70	25.9
832.	Micromonospora endolithica DSM 44398	Micromonospora trifolii NIE79	86.19	81.07	25.9
833.	Micromonospora endolithica DSM 44398	Micromonospora zamorensis DSM 45600	86.26	81.12	25.9
834.	Micromonospora endophytica DSM 45430	Micromonospora coxensis DSM 45161	86.27	80.61	25.9
835.	Micromonospora endophytica DSM 45430	Micromonospora endolithica DSM 44398	86.16	81.00	25.9
836.	Micromonospora endophytica DSM 45430	Micromonospora thermarum HSS6-12	86.21	80.92	25.9
837.	Micromonospora halophytica DSM 43171	Micromonospora saelicesensis DSM 44871	86.17	82.16	25.9
838.	Micromonospora harpali NEAU-JC6	Micromonospora nigra DSM 43818	86.40	81.64	25.9
839.	Micromonospora jinlongensis DSM 45876	Micromonospora lacuserhaii CPCC 205547	86.35	82.08	25.9
840.	Micromonospora lacuserhaii CPCC 205547	Micromonospora zamorensis DSM 45600	86.37	82.06	25.9
841.	Micromonospora marina PCU 269	Micromonospora harpali NEAU-JC6	86.50	82.04	25.9
842.	Micromonospora noduli GUI43	Micromonospora harpali NEAU-JC6	86.26	80.96	25.9
843.	Micromonospora noduli GUI43	Micromonospora maritima DSM 45782	86.17	81.13	25.9
844.	Micromonospora orduensis S2509	Micromonospora mirobrigensis DSM 44830	86.33	81.97	25.9
845.	Micromonospora oryzae DSM 102119	Micromonospora nigra DSM 43818	86.43	81.59	25.9
846.	Micromonospora parathelypteridis DSM 103125	Micromonospora coxensis DSM 45161	86.31	80.92	25.9
847.	Micromonospora profundi DSM 45981	Micromonospora coxensis DSM 45161	86.20	81.02	25.9
848.	Micromonospora profundi DSM 45981	Micromonospora lacuserhaii CPCC 205547	86.20	81.74	25.9
849.	Micromonospora profundi DSM 45981	Micromonospora psammae CPCC 205556	86.30	81.92	25.9
850.	Micromonospora psammae CPCC 205556	Micromonospora chokoriensis DSM 45160	86.39	82.07	25.9
851.	Micromonospora rubida NEAU-HG-1	Micromonospora profundi DSM 45981	86.16	80.94	25.9
852.	Micromonospora rubida NEAU-HG-1	Micromonospora zamorensis DSM 45600	86.43	81.08	25.9
853.	Micromonospora salmantinae PSH03	Micromonospora halophytica DSM 43171	86.20	81.60	25.9
854.	Micromonospora sediminimaris NBRC 107745	Micromonospora nigra DSM 43818	86.05	81.29	25.9
855.	Micromonospora sonchi CGMCC 4.7312	Micromonospora purpureochromogenes DSM 43821	86.34	80.90	25.9

856.	Micromonospora taraxaci DSM 45885	Micromonospora psammae CPCC 205556	86.30	82.06	25.9
857.	Micromonospora thermarum HSS6-12	Micromonospora cabrerizensis LAH09	86.11	81.97	25.9
858.	Micromonospora thermarum HSS6-12	Micromonospora schwarzwaldensis DSM 45708	86.38	81.80	25.9
859.	Micromonospora vinacea DSM 101695	Micromonospora halophytica DSM 43171	86.23	81.86	25.9
860.	Micromonospora violae DSM 45888	Micromonospora endolithica DSM 44398	86.27	81.13	25.9
861.	Micromonospora violae DSM 45888	Micromonospora halophytica DSM 43171	86.15	81.58	25.9
862.	Micromonospora violae DSM 45888	Micromonospora harpali NEAU-JC6	86.22	80.93	25.9
863.	Micromonospora violae DSM 45888	Micromonospora maritima DSM 45782	86.16	80.99	25.9
864.	Micromonospora zingiberis PLAI 1-1	Micromonospora thermarum HSS6-12	86.25	81.71	25.9
865.	Micromonospora alfalfae MED01	Micromonospora maritima DSM 45782	86.12	80.94	25.8
866.	Micromonospora antibiotica MMS20-R2-23	Micromonospora lupini JCM 16031	86.25	80.62	25.8
867.	Micromonospora arida LB32	Micromonospora maritima DSM 45782	86.19	81.30	25.8
868.	Micromonospora aurantiaca ATCC 27029	Micromonospora acroterricola 5R2A7	86.20	81.02	25.8
869.	Micromonospora aurantiaca ATCC 27029	Micromonospora antibiotica MMS20-R2-23	86.31	81.11	25.8
870.	Micromonospora brunnea DSM 43814	Micromonospora phaseoli CGMCC 4.7038	86.19	81.05	25.8
871.	Micromonospora endolithica DSM 44398	Micromonospora alfalfae MED01	86.15	81.59	25.8
872.	Micromonospora endolithica DSM 44398	Micromonospora jinlongensis DSM 45876	86.24	81.28	25.8
873.	Micromonospora endolithica DSM 44398	Micromonospora schwarzwaldensis DSM 45708	86.33	81.32	25.8
874.	Micromonospora endolithica DSM 44398	Micromonospora sonchi CGMCC 4.7312	86.21	81.08	25.8
875.	Micromonospora endophytica DSM 45430	Micromonospora brunnea DSM 43814	86.31	80.52	25.8
876.	Micromonospora endophytica DSM 45430	Micromonospora palomenae DSM 102131	86.30	80.54	25.8
877.	Micromonospora foliorum PSH25	Micromonospora schwarzwaldensis DSM 45708	86.13	80.64	25.8
878.	Micromonospora foliorum PSH25	Micromonospora sediminicola DSM 45794	86.16	80.73	25.8
879.	Micromonospora hortensis NIE111	Micromonospora halophytica DSM 43171	86.11	81.28	25.8
880.	Micromonospora mirobrigensis DSM 44830	Micromonospora nigra DSM 43818	86.27	81.29	25.8

881.	Micromonospora noduli GUI43	Micromonospora oryzae DSM 102119	86.23	80.79	25.8
882.	Micromonospora noduli GUI43	Micromonospora rifamycinica DSM 44983	86.08	80.52	25.8
883.	Micromonospora noduli GUI43	Micromonospora thermarum HSS6-12	86.08	81.95	25.8
884.	Micromonospora oryzae DSM 102119	Micromonospora marina PCU 269	86.48	82.00	25.8
885.	Micromonospora rubida NEAU-HG-1	Micromonospora cabrerizensis LAH09	86.35	80.96	25.8
886.	Micromonospora saelicesensis DSM 44871	Micromonospora maritima DSM 45782	86.15	81.41	25.8
887.	Micromonospora salmantinae PSH03	Micromonospora harpali NEAU-JC6	86.17	80.81	25.8
888.	Micromonospora salmantinae PSH03	Micromonospora maritima DSM 45782	86.17	81.05	25.8
889.	Micromonospora sonchi CGMCC 4.7312	Micromonospora maritima DSM 45782	86.21	80.25	25.8
890.	Micromonospora taraxaci DSM 45885	Micromonospora lacuserhaii CPCC 205547	86.27	81.76	25.8
891.	Micromonospora thermarum HSS6-12	Micromonospora jinlongensis DSM 45876	86.10	81.87	25.8
892.	Micromonospora thermarum HSS6-12	Micromonospora parathelypteridis DSM 103125	86.09	81.89	25.8
893.	Micromonospora thermarum HSS6-12	Micromonospora robiginosa 28ISP2-46	86.42	81.93	25.8
894.	Micromonospora thermarum HSS6-12	Micromonospora salmantinae PSH03	86.01	81.84	25.8
895.	Micromonospora thermarum HSS6-12	Micromonospora vinacea DSM 101695	86.09	81.89	25.8
896.	Micromonospora thermarum HSS6-12	Micromonospora zamorensis DSM 45600	86.04	81.90	25.8
897.	Micromonospora ureilytica DSM 101692	Micromonospora halophytica DSM 43171	86.20	81.24	25.8
898.	Micromonospora vinacea DSM 101695	Micromonospora harpali NEAU-JC6	86.26	80.83	25.8
899.	Micromonospora vinacea DSM 101695	Micromonospora maritima DSM 45782	86.10	81.03	25.8
900.	Micromonospora vinacea DSM 101695	Micromonospora oryzae DSM 102119	86.23	80.74	25.8
901.	Micromonospora violae DSM 45888	Micromonospora oryzae DSM 102119	86.23	80.85	25.8
902.	Micromonospora violae DSM 45888	Micromonospora rifamycinica DSM 44983	86.11	80.43	25.8
903.	Micromonospora violae DSM 45888	MMicromonospora solifontis PPF5-17	86.17	81.67	25.8
904.	Micromonospora zingiberis PLAI 1-1	Micromonospora coxensis DSM 45161	86.38	81.17	25.8
905.	Micromonospora acroterricola 5R2A7	Micromonospora endophytica DSM 45430	86.01	80.40	25.7

906.	Micromonospora acroterricola 5R2A7	Micromonospora fluminis A38	86.24	81.17	25.7
907.	Micromonospora acroterricola 5R2A7	Micromonospora sonchi CGMCC 4.7312	86.00	80.76	25.7
908.	Micromonospora acroterricola 5R2A7	Micromonospora zingiberis PLAI 1-1	86.03	80.67	25.7
909.	Micromonospora alfalfae MED01	Micromonospora halophytica DSM 43171	86.10	81.62	25.7
910.	Micromonospora antibiotica MMS20-R2-23	Micromonospora marina PCU 269	86.32	81.20	25.7
911.	Micromonospora arida LB32	Micromonospora antibiotica MMS20-R2-23	86.09	80.49	25.7
912.	Micromonospora arida LB32	Micromonospora mirobrigensis DSM 44830	86.17	81.59	25.7
913.	Micromonospora arida LB32	MMicromonospora solifontis PPF5-17	86.20	81.74	25.7
914.	Micromonospora aurantiaca ATCC 27029	Micromonospora thermarum HSS6-12	86.38	81.67	25.7
915.	Micromonospora brunnea DSM 43814	Micromonospora qiuiae NBRC 106684	86.21	80.51	25.7
916.	Micromonospora cabrerizensis LAH09	Micromonospora coxensis DSM 45161	86.35	81.13	25.7
917.	Micromonospora cabrerizensis LAH09	Micromonospora maritima DSM 45782	86.18	81.17	25.7
918.	Micromonospora chalcea DSM 43026	Micromonospora acroterricola 5R2A7	86.24	81.01	25.7
919.	Micromonospora chalcea DSM 43026	Micromonospora antibiotica MMS20-R2-23	86.32	80.82	25.7
920.	Micromonospora endolithica DSM 44398	Micromonospora fluminis A38	86.31	81.03	25.7
921.	Micromonospora endolithica DSM 44398	Micromonospora palythoicola S2-005	86.14	81.31	25.7
922.	Micromonospora endolithica DSM 44398	Micromonospora profundi DSM 45981	86.08	81.21	25.7
923.	Micromonospora endolithica DSM 44398	Micromonospora qiuiae NBRC 106684	86.10	80.65	25.7
924.	Micromonospora endolithica DSM 44398	Micromonospora robiginosa 28ISP2-46	86.35	81.44	25.7
925.	Micromonospora endophytica DSM 45430	Micromonospora halophytica DSM 43171	86.20	80.57	25.7
926.	Micromonospora endophytica DSM 45430	Micromonospora maritima DSM 45782	86.22	80.30	25.7
927.	Micromonospora endophytica DSM 45430	Micromonospora purpureochromogenes DSM 43821	86.22	80.46	25.7
928.	Micromonospora harpali NEAU-JC6	Micromonospora saelicesensis DSM 44871	86.20	81.11	25.7
929.	Micromonospora hortensis NIE111	Micromonospora harpali NEAU-JC6	86.14	80.72	25.7
930.	Micromonospora hortensis NIE111	Micromonospora maritima DSM 45782	86.06	80.90	25.7

931.	Micromonospora hortensis NIE111	Micromonospora oryzae DSM 102119	86.13	80.68	25.7
932.	Micromonospora jinlongensis DSM 45876	Micromonospora coxensis DSM 45161	86.31	81.15	25.7
933.	Micromonospora lupini JCM 16031	Micromonospora mirobrigensis DSM 44830	86.29	81.65	25.7
934.	Micromonospora noduli GUI43	MMicromonospora solifontis PPF5-17	86.21	81.76	25.7
935.	Micromonospora orduensis S2509	Micromonospora nigra DSM 43818	85.92	81.09	25.7
936.	Micromonospora parathelypteridis DSM 103125	Micromonospora halophytica DSM 43171	86.23	81.12	25.7
937.	Micromonospora parathelypteridis DSM 103125	Micromonospora harpali NEAU-JC6	86.18	80.69	25.7
938.	Micromonospora parathelypteridis DSM 103125	Micromonospora oryzae DSM 102119	86.15	80.66	25.7
939.	Micromonospora profundi DSM 45981	Micromonospora maritima DSM 45782	86.06	80.95	25.7
940.	Micromonospora purpurea DSM 43036	Micromonospora antibiotica MMS20-R2-23	86.26	80.91	25.7
941.	Micromonospora qiuiae NBRC 106684	Micromonospora halophytica DSM 43171	86.33	80.18	25.7
942.	Micromonospora robiginosa 28ISP2-46	Micromonospora foliorum PSH25	86.14	80.62	25.7
943.	Micromonospora rubida NEAU-HG-1	Micromonospora jinlongensis DSM 45876	86.34	80.98	25.7
944.	Micromonospora salmantinae PSH03	Micromonospora oryzae DSM 102119	86.18	80.75	25.7
945.	Micromonospora salmantinae PSH03	Micromonospora rifamycinica DSM 44983	86.08	80.58	25.7
946.	Micromonospora sonchi CGMCC 4.7312	Micromonospora psammae CPCC 205556	86.26	80.81	25.7
947.	Micromonospora taraxaci DSM 45885	Micromonospora endolithica DSM 44398	86.07	80.84	25.7
948.	Micromonospora thermarum HSS6-12	Micromonospora chokoriensis DSM 45160	86.08	81.82	25.7
949.	Micromonospora thermarum HSS6-12	Micromonospora fluminis A38	86.35	81.77	25.7
950.	Micromonospora thermarum HSS6-12	Micromonospora palythoicola S2-005	86.19	81.34	25.7
951.	Micromonospora thermarum HSS6-12	Micromonospora phaseoli CGMCC 4.7038	86.13	81.66	25.7
952.	Micromonospora thermarum HSS6-12	Micromonospora saelicesensis DSM 44871	86.05	81.96	25.7
953.	Micromonospora thermarum HSS6-12	Micromonospora trifolii NIE79	85.99	81.79	25.7
954.	Micromonospora trifolii NIE79	Micromonospora halophytica DSM 43171	86.11	81.40	25.7
955.	Micromonospora trifolii NIE79	Micromonospora harpali NEAU-JC6	86.14	80.84	25.7

956.	Micromonospora trifolii NIE79	Micromonospora maritima DSM 45782	86.14	80.87	25.7
957.	Micromonospora trifolii NIE79	Micromonospora oryzae DSM 102119	86.14	80.77	25.7
958.	Micromonospora ureilytica DSM 101692	Micromonospora harpali NEAU-JC6	86.15	80.53	25.7
959.	Micromonospora ureilytica DSM 101692	Micromonospora maritima DSM 45782	86.12	80.77	25.7
960.	Micromonospora ureilytica DSM 101692	Micromonospora oryzae DSM 102119	86.13	80.46	25.7
961.	Micromonospora ureilytica DSM 101692	Micromonospora rifamycinica DSM 44983	86.06	80.46	25.7
962.	Micromonospora vinacea DSM 101695	Micromonospora rifamycinica DSM 44983	86.16	80.44	25.7
963.	Micromonospora violae DSM 45888	Micromonospora thermarum HSS6-12	86.11	81.79	25.7
964.	Micromonospora zingiberis PLAI 1-1	Micromonospora maritima DSM 45782	86.16	80.33	25.7
965.	MMicromonospora solifontis PPF5-17	Micromonospora parathelypteridis DSM 103125	86.14	81.48	25.7
966.	Micromonospora acroterricola 5R2A7	Micromonospora purpurea DSM 43036	86.19	81.06	25.6
967.	Micromonospora acroterricola 5R2A7	Micromonospora tulbaghiae DSM 45142	86.12	81.06	25.6
968.	Micromonospora alfalfae MED01	Micromonospora harpali NEAU-JC6	86.11	80.70	25.6
969.	Micromonospora alfalfae MED01	Micromonospora rifamycinica DSM 44983	86.02	81.09	25.6
970.	Micromonospora aurantiaca ATCC 27029	Micromonospora endolithica DSM 44398	86.42	81.12	25.6
971.	Micromonospora cabrerizensis LAH09	Micromonospora mirobrigensis DSM 44830	86.22	81.64	25.6
972.	Micromonospora chokoriensis DSM 45160	Micromonospora maritima DSM 45782	86.17	80.99	25.6
973.	Micromonospora coxensis DSM 45161	Micromonospora chokoriensis DSM 45160	86.30	80.95	25.6
974.	Micromonospora endolithica DSM 44398	Micromonospora humi DSM 45647	86.41	81.37	25.6
975.	Micromonospora endophytica DSM 45430	Micromonospora foliorum PSH25	85.96	80.09	25.6
976.	Micromonospora endophytica DSM 45430	Micromonospora psammae CPCC 205556	86.24	80.43	25.6
977.	Micromonospora endophytica DSM 45430	Micromonospora rubida NEAU-HG-1	86.10	80.11	25.6
978.	Micromonospora foliorum PSH25	Micromonospora humi DSM 45647	86.13	80.62	25.6
979.	Micromonospora hortensis NIE111	Micromonospora rifamycinica DSM 44983	86.02	80.44	25.6
980.	Micromonospora jinlongensis DSM 45876	Micromonospora maritima DSM 45782	86.18	81.11	25.6

981.	Micromonospora lutea NBRC 106530	Micromonospora nigra DSM 43818	85.91	81.03	25.6
982.	Micromonospora orduensis S2509	Micromonospora schwarzwaldensis DSM 45708	86.11	80.96	25.6
983.	Micromonospora oryzae DSM 102119	Micromonospora saelicesensis DSM 44871	86.19	81.03	25.6
984.	Micromonospora palomenae DSM 102131	Micromonospora phaseoli CGMCC 4.7038	86.18	80.99	25.6
985.	Micromonospora palomenae DSM 102131	Micromonospora qiuiae NBRC 106684	86.24	80.41	25.6
986.	Micromonospora parathelypteridis DSM 103125	Micromonospora maritima DSM 45782	86.05	80.86	25.6
987.	Micromonospora profundi DSM 45981	Micromonospora halophytica DSM 43171	86.09	81.37	25.6
988.	Micromonospora profundi DSM 45981	Micromonospora harpali NEAU-JC6	86.02	80.58	25.6
989.	Micromonospora qiuiae NBRC 106684	Micromonospora coxensis DSM 45161	86.19	80.29	25.6
990.	Micromonospora qiuiae NBRC 106684	Micromonospora maritima DSM 45782	86.09	79.91	25.6
991.	Micromonospora qiuiae NBRC 106684	Micromonospora purpureochromogenes DSM 43821	86.21	80.30	25.6
992.	Micromonospora rifamycinica DSM 44983	Micromonospora saelicesensis DSM 44871	86.08	80.63	25.6
993.	Micromonospora rubida NEAU-HG-1	Micromonospora chokoriensis DSM 45160	86.24	80.77	25.6
994.	Micromonospora rubida NEAU-HG-1	Micromonospora qiuiae NBRC 106684	86.07	79.74	25.6
995.	Micromonospora rubida NEAU-HG-1	Micromonospora sonchi CGMCC 4.7312	86.11	80.51	25.6
996.	Micromonospora sonchi CGMCC 4.7312	Micromonospora halophytica DSM 43171	86.23	80.48	25.6
997.	Micromonospora taraxaci DSM 45885	Micromonospora thermarum HSS6-12	86.00	81.85	25.6
998.	Micromonospora thermarum HSS6-12	Micromonospora antibiotica MMS20-R2-23	86.21	81.32	25.6
999.	Micromonospora thermarum HSS6-12	Micromonospora hortensis NIE111	85.97	81.66	25.6
1000.	Micromonospora thermarum HSS6-12	Micromonospora humi DSM 45647	86.28	81.93	25.6
1001.	Micromonospora thermarum HSS6-12	Micromonospora ureilytica DSM 101692	85.99	81.60	25.6
1002.	Micromonospora trifolii NIE79	Micromonospora rifamycinica DSM 44983	86.05	80.42	25.6
1003.	Micromonospora vinacea DSM 101695	Micromonospora antibiotica MMS20-R2-23	86.04	80.47	25.6
1004.	Micromonospora violae DSM 45888	Micromonospora antibiotica MMS20-R2-23	86.11	80.43	25.6
1005.	Micromonospora violae DSM 45888	Micromonospora mirobrigensis DSM 44830	86.19	81.49	25.6

1006.	Micromonospora vulcania CGMCC 4.7144	Micromonospora nigra DSM 43818	86.07	80.94	25.6
1007.	Micromonospora zamorensis DSM 45600	Micromonospora maritima DSM 45782	86.13	81.07	25.6
1008.	Micromonospora zingiberis PLAI 1-1	Micromonospora endolithica DSM 44398	86.17	81.15	25.6
1009.	MMicromonospora solifontis PPF5-17	Micromonospora cabrerizensis LAH09	85.71	81.21	25.6
1010.	MMicromonospora solifontis PPF5-17	Micromonospora jinlongensis DSM 45876	85.62	80.21	25.6
1011.	MMicromonospora solifontis PPF5-17	Micromonospora saelicesensis DSM 44871	86.20	81.98	25.6
1012.	MMicromonospora solifontis PPF5-17	Micromonospora salmantinae PSH03	86.16	81.65	25.6
1013.	MMicromonospora solifontis PPF5-17	Micromonospora sonchi CGMCC 4.7312	86.23	80.94	25.6
1014.	MMicromonospora solifontis PPF5-17	Micromonospora trifolii NIE79	86.13	81.59	25.6
1015.	MMicromonospora solifontis PPF5-17	Micromonospora vinacea DSM 101695	86.17	81.67	25.6
1016.	Micromonospora acroterricola 5R2A7	Micromonospora marina PCU 269	86.12	81.38	25.5
1017.	Micromonospora acroterricola 5R2A7	Micromonospora phaseoli CGMCC 4.7038	85.88	80.76	25.5
1018.	Micromonospora alfalfae MED01	Micromonospora oryzae DSM 102119	86.10	80.64	25.5
1019.	Micromonospora antibiotica MMS20-R2-23	Micromonospora hortensis NIE111	86.02	80.22	25.5
1020.	Micromonospora antibiotica MMS20-R2-23	Micromonospora saelicesensis DSM 44871	86.05	80.58	25.5
1021.	Micromonospora antibiotica MMS20-R2-23	Micromonospora salmantinae PSH03	86.02	80.47	25.5
1022.	Micromonospora antibiotica MMS20-R2-23	Micromonospora trifolii NIE79	85.96	80.40	25.5
1023.	Micromonospora brunnea DSM 43814	Micromonospora fiedleri MG-37	86.28	80.33	25.5
1024.	Micromonospora brunnea DSM 43814	Micromonospora palythoicola S2-005	86.16	80.73	25.5
1025.	Micromonospora chalcea DSM 43026	Micromonospora endolithica DSM 44398	86.33	81.14	25.5
1026.	Micromonospora chalcea DSM 43026	Micromonospora thermarum HSS6-12	86.40	81.59	25.5
1027.	Micromonospora chokoriensis DSM 45160	Micromonospora mirobrigensis DSM 44830	86.08	81.48	25.5
1028.	Micromonospora coxensis DSM 45161	Micromonospora phaseoli CGMCC 4.7038	86.13	80.81	25.5
1029.	Micromonospora endolithica DSM 44398	Micromonospora tulbaghiae DSM 45142	86.27	81.20	25.5
1030.	Micromonospora endophytica DSM 45430	Micromonospora lacuserhaii CPCC 205547	86.18	80.37	25.5

1031.	Micromonospora endophytica DSM 45430	Micromonospora oryzae DSM 102119	86.16	80.54	25.5
1032.	Micromonospora endophytica DSM 45430	MMicromonospora solifontis PPF5-17	86.20	80.82	25.5
1033.	Micromonospora fiedleri MG-37	Micromonospora coxensis DSM 45161	86.26	80.29	25.5
1034.	Micromonospora hortensis NIE111	Micromonospora mirobrigensis DSM 44830	86.12	81.32	25.5
1035.	Micromonospora jinlongensis DSM 45876	Micromonospora mirobrigensis DSM 44830	86.19	81.59	25.5
1036.	Micromonospora jinlongensis DSM 45876	Micromonospora oryzae DSM 102119	86.21	80.78	25.5
1037.	Micromonospora lupini JCM 16031	Micromonospora humi DSM 45647	86.07	81.32	25.5
1038.	Micromonospora lupini JCM 16031	Micromonospora nigra DSM 43818	85.87	80.96	25.5
1039.	Micromonospora lupini JCM 16031	Micromonospora schwarzwaldensis DSM 45708	86.16	80.83	25.5
1040.	Micromonospora lupini JCM 16031	Micromonospora sediminicola DSM 45794	86.16	81.48	25.5
1041.	Micromonospora mirobrigensis DSM 44830	Micromonospora saelicesensis DSM 44871	86.18	81.75	25.5
1042.	Micromonospora noduli GUI43	Micromonospora antibiotica MMS20-R2-23	86.09	80.37	25.5
1043.	Micromonospora noduli GUI43	Micromonospora mirobrigensis DSM 44830	86.19	81.57	25.5
1044.	Micromonospora orduensis S2509	Micromonospora sediminicola DSM 45794	86.14	81.09	25.5
1045.	Micromonospora phaseoli CGMCC 4.7038	Micromonospora maritima DSM 45782	86.07	80.42	25.5
1046.	Micromonospora profundi DSM 45981	Micromonospora oryzae DSM 102119	86.06	80.64	25.5
1047.	Micromonospora purpureochromogenes DSM 43821	Micromonospora phaseoli CGMCC 4.7038	86.13	80.94	25.5
1048.	Micromonospora qiuiae NBRC 106684	Micromonospora lacuserhaii CPCC 205547	86.13	80.58	25.5
1049.	Micromonospora robiginosa 28ISP2-46	Micromonospora lupini JCM 16031	86.08	81.30	25.5
1050.	Micromonospora rubida NEAU-HG-1	Micromonospora phaseoli CGMCC 4.7038	86.01	80.35	25.5
1051.	Micromonospora salmantinae PSH03	Micromonospora mirobrigensis DSM 44830	86.16	81.53	25.5
1052.	Micromonospora sonchi CGMCC 4.7312	Micromonospora foliorum PSH25	85.85	79.90	25.5
1053.	Micromonospora sonchi CGMCC 4.7312	Micromonospora lacuserhaii CPCC 205547	86.18	80.80	25.5
1054.	Micromonospora taraxaci DSM 45885	Micromonospora maritima DSM 45782	86.07	80.96	25.5
1055.	Micromonospora taraxaci DSM 45885	Micromonospora rubida NEAU-HG-1	86.21	80.71	25.5

1056.	Micromonospora thermarum HSS6-12	Micromonospora alfalfae MED01	85.88	81.64	25.5
1057.	Micromonospora thermarum HSS6-12	Micromonospora purpurea DSM 43036	86.30	81.65	25.5
1058.	Micromonospora thermarum HSS6-12	Micromonospora sediminimaris NBRC 107745	86.11	80.94	25.5
1059.	Micromonospora thermarum HSS6-12	Micromonospora tulbaghiae DSM 45142	86.31	81.77	25.5
1060.	Micromonospora ureilytica DSM 101692	Micromonospora antibiotica MMS20-R2-23	85.98	80.38	25.5
1061.	Micromonospora ureilytica DSM 101692	Micromonospora mirobrigensis DSM 44830	86.20	81.23	25.5
1062.	Micromonospora vinacea DSM 101695	Micromonospora mirobrigensis DSM 44830	86.22	81.61	25.5
1063.	Micromonospora zamorensis DSM 45600	Micromonospora coxensis DSM 45161	86.22	80.96	25.5
1064.	Micromonospora zingiberis PLAI 1-1	Micromonospora brunnea DSM 43814	86.32	80.99	25.5
1065.	Micromonospora zingiberis PLAI 1-1	Micromonospora halophytica DSM 43171	86.22	80.41	25.5
1066.	MMicromonospora solifontis PPF5-17	Micromonospora chokoriensis DSM 45160	85.62	80.19	25.5
1067.	MMicromonospora solifontis PPF5-17	Micromonospora hortensis NIE111	85.59	80.06	25.5
1068.	MMicromonospora solifontis PPF5-17	Micromonospora profundi DSM 45981	85.93	81.34	25.5
1069.	MMicromonospora solifontis PPF5-17	Micromonospora ureilytica DSM 101692	86.09	81.54	25.5
1070.	MMicromonospora solifontis PPF5-17	Micromonospora zamorensis DSM 45600	86.07	81.53	25.5
1071.	Micromonospora acroterricola 5R2A7	Micromonospora qiuiae NBRC 106684	85.88	80.46	25.4
1072.	Micromonospora antibiotica MMS20-R2-23	Micromonospora cabrerizensis LAH09	86.07	80.40	25.4
1073.	Micromonospora antibiotica MMS20-R2-23	Micromonospora zamorensis DSM 45600	86.04	80.20	25.4
1074.	Micromonospora arida LB32	Micromonospora nigra DSM 43818	85.82	81.22	25.4
1075.	Micromonospora aurantiaca ATCC 27029	Micromonospora foliorum PSH25	86.02	80.15	25.4
1076.	Micromonospora cabrerizensis LAH09	Micromonospora harpali NEAU-JC6	86.20	80.80	25.4
1077.	Micromonospora cabrerizensis LAH09	Micromonospora oryzae DSM 102119	86.25	80.79	25.4
1078.	Micromonospora cabrerizensis LAH09	Micromonospora rifamycinica DSM 44983	86.09	80.40	25.4
1079.	Micromonospora endolithica DSM 44398	Micromonospora fiedleri MG-37	86.12	80.72	25.4
1080.	Micromonospora endolithica DSM 44398	Micromonospora purpurea DSM 43036	86.27	80.94	25.4

1081.	Micromonospora fiedleri MG-37	Micromonospora maritima DSM 45782	86.16	80.14	25.4
1082.	Micromonospora foliorum PSH25	Micromonospora phaseoli CGMCC 4.7038	85.81	80.33	25.4
1083.	Micromonospora harpali NEAU-JC6	Micromonospora zamorensis DSM 45600	86.15	80.65	25.4
1084.	Micromonospora jinlongensis DSM 45876	Micromonospora antibiotica MMS20-R2-23	86.11	80.39	25.4
1085.	Micromonospora jinlongensis DSM 45876	Micromonospora harpali NEAU-JC6	86.19	80.80	25.4
1086.	Micromonospora jinlongensis DSM 45876	Micromonospora rifamycinica DSM 44983	86.08	80.45	25.4
1087.	Micromonospora noduli GUI43	Micromonospora nigra DSM 43818	85.78	80.87	25.4
1088.	Micromonospora orduensis S2509	Micromonospora humi DSM 45647	86.07	81.02	25.4
1089.	Micromonospora orduensis S2509	Micromonospora robiginosa 28ISP2-46	86.10	80.87	25.4
1090.	Micromonospora palomenae DSM 102131	Micromonospora fiedleri MG-37	86.29	80.30	25.4
1091.	Micromonospora palythoicola S2-005	Micromonospora coxensis DSM 45161	86.10	80.66	25.4
1092.	Micromonospora palythoicola S2-005	Micromonospora oryzae DSM 102119	86.01	80.42	25.4
1093.	Micromonospora parathelypteridis DSM 103125	Micromonospora antibiotica MMS20-R2-23	86.02	80.14	25.4
1094.	Micromonospora parathelypteridis DSM 103125	Micromonospora mirobrigensis DSM 44830	86.17	81.26	25.4
1095.	Micromonospora parathelypteridis DSM 103125	Micromonospora rifamycinica DSM 44983	86.08	80.26	25.4
1096.	Micromonospora profundi DSM 45981	Micromonospora thermarum HSS6-12	85.90	81.61	25.4
1097.	Micromonospora psammae CPCC 205556	Micromonospora phaseoli CGMCC 4.7038	86.17	80.78	25.4
1098.	Micromonospora qiuiae NBRC 106684	Micromonospora oryzae DSM 102119	86.08	79.82	25.4
1099.	Micromonospora qiuiae NBRC 106684	Micromonospora psammae CPCC 205556	86.23	80.95	25.4
1100.	Micromonospora rifamycinica DSM 44983	Micromonospora zamorensis DSM 45600	86.05	80.30	25.4
1101.	Micromonospora robiginosa 28ISP2-46	Micromonospora vulcania CGMCC 4.7144	86.15	80.95	25.4
1102.	Micromonospora rubida NEAU-HG-1	Micromonospora palythoicola S2-005	85.95	80.36	25.4
1103.	Micromonospora schwarzwaldensis DSM 45708	Micromonospora vulcania CGMCC 4.7144	86.18	80.95	25.4
1104.	Micromonospora sonchi CGMCC 4.7312	Micromonospora oryzae DSM 102119	86.14	80.48	25.4
1105.	Micromonospora taraxaci DSM 45885	Micromonospora coxensis DSM 45161	86.18	80.57	25.4

1106.	Micromonospora thermarum HSS6-12	Micromonospora andamanensis NBRC 109075	86.02	81.44	25.4
1107.	Micromonospora thermarum HSS6-12	Micromonospora fiedleri MG-37	86.18	81.39	25.4
1108.	Micromonospora thermarum HSS6-12	Micromonospora marina PCU 269	86.25	81.76	25.4
1109.	Micromonospora trifolii NIE79	Micromonospora mirobrigensis DSM 44830	86.13	81.46	25.4
1110.	Micromonospora vulcania CGMCC 4.7144	Micromonospora sediminicola DSM 45794	86.17	80.98	25.4
1111.	Micromonospora zamorensis DSM 45600	Micromonospora mirobrigensis DSM 44830	86.14	81.57	25.4
1112.	Micromonospora zingiberis PLAI 1-1	Micromonospora foliorum PSH25	85.92	79.78	25.4
1113.	Micromonospora zingiberis PLAI 1-1	Micromonospora palomenae DSM 102131	86.35	80.87	25.4
1114.	Micromonospora zingiberis PLAI 1-1	Micromonospora psammae CPCC 205556	86.29	80.79	25.4
1115.	Micromonospora zingiberis PLAI 1-1	Micromonospora purpureochromogenes DSM 43821	86.25	80.90	25.4
1116.	Micromonospora zingiberis PLAI 1-1	MMicromonospora solifontis PPF5-17	86.15	80.90	25.4
1117.	MMicromonospora solifontis PPF5-17	Micromonospora alfalfae MED01	85.53	80.12	25.4
1118.	MMicromonospora solifontis PPF5-17	Micromonospora qiuiae NBRC 106684	86.15	80.62	25.4
1119.	Micromonospora acroterricola 5R2A7	Micromonospora fiedleri MG-37	85.96	80.42	25.3
1120.	Micromonospora acroterricola 5R2A7	Micromonospora palythoicola S2-005	85.84	80.71	25.3
1121.	Micromonospora alfalfae MED01	Micromonospora mirobrigensis DSM 44830	86.03	81.49	25.3
1122.	Micromonospora antibiotica MMS20-R2-23	Micromonospora alfalfae MED01	85.98	80.11	25.3
1123.	Micromonospora aurantiaca ATCC 27029	Micromonospora orduensis S2509	86.10	80.76	25.3
1124.	Micromonospora cabrerizensis LAH09	Micromonospora halophytica DSM 43171	86.24	81.30	25.3
1125.	Micromonospora chalcea DSM 43026	Micromonospora foliorum PSH25	86.03	80.17	25.3
1126.	Micromonospora chalcea DSM 43026	Micromonospora orduensis S2509	86.12	80.60	25.3
1127.	Micromonospora endolithica DSM 44398	Micromonospora marina PCU 269	86.22	81.29	25.3
1128.	Micromonospora endophytica DSM 45430	Micromonospora harpali NEAU-JC6	86.12	80.47	25.3
1129.	Micromonospora foliorum PSH25	Micromonospora fluminis A38	86.03	80.24	25.3
1130.	Micromonospora foliorum PSH25	Micromonospora marina PCU 269	85.96	80.89	25.3

1131.	Micromonospora halophytica DSM 43171	Micromonospora phaseoli CGMCC 4.7038	86.03	80.98	25.3
1132.	Micromonospora halophytica DSM 43171	Micromonospora zamorensis DSM 45600	86.12	81.25	25.3
1133.	Micromonospora harpali NEAU-JC6	Micromonospora chokoriensis DSM 45160	86.13	80.75	25.3
1134.	Micromonospora jinlongensis DSM 45876	Micromonospora halophytica DSM 43171	86.15	81.30	25.3
1135.	Micromonospora orduensis S2509	Micromonospora fluminis A38	86.08	80.64	25.3
1136.	Micromonospora oryzae DSM 102119	Micromonospora chokoriensis DSM 45160	86.13	80.66	25.3
1137.	Micromonospora oryzae DSM 102119	Micromonospora zamorensis DSM 45600	86.18	80.69	25.3
1138.	Micromonospora palomenae DSM 102131	Micromonospora palythoicola S2-005	86.14	80.68	25.3
1139.	Micromonospora palythoicola S2-005	Micromonospora harpali NEAU-JC6	85.99	80.39	25.3
1140.	Micromonospora palythoicola S2-005	Micromonospora lacuserhaii CPCC 205547	86.01	80.58	25.3
1141.	Micromonospora palythoicola S2-005	Micromonospora maritima DSM 45782	86.06	80.13	25.3
1142.	Micromonospora palythoicola S2-005	Micromonospora purpureochromogenes DSM 43821	86.13	80.67	25.3
1143.	Micromonospora profundi DSM 45981	Micromonospora rifamycinica DSM 44983	85.96	80.49	25.3
1144.	Micromonospora qiuiae NBRC 106684	Micromonospora foliorum PSH25	85.77	79.73	25.3
1145.	Micromonospora qiuiae NBRC 106684	Micromonospora harpali NEAU-JC6	86.02	79.83	25.3
1146.	Micromonospora rifamycinica DSM 44983	Micromonospora nigra DSM 43818	86.18	80.63	25.3
1147.	Micromonospora salmantinae PSH03	Micromonospora nigra DSM 43818	85.85	80.75	25.3
1148.	Micromonospora sediminicola DSM 45794	Micromonospora nigra DSM 43818	86.07	80.89	25.3
1149.	Micromonospora sonchi CGMCC 4.7312	Micromonospora harpali NEAU-JC6	86.12	80.45	25.3
1150.	Micromonospora sonchi CGMCC 4.7312	Micromonospora rifamycinica DSM 44983	85.96	80.14	25.3
1151.	Micromonospora taraxaci DSM 45885	Micromonospora harpali NEAU-JC6	86.05	80.50	25.3
1152.	Micromonospora taraxaci DSM 45885	Micromonospora oryzae DSM 102119	86.04	80.50	25.3
1153.	Micromonospora taraxaci DSM 45885	MMicromonospora solifontis PPF5-17	85.99	81.47	25.3
1154.	Micromonospora vulcania CGMCC 4.7144	Micromonospora humi DSM 45647	86.12	80.89	25.3
1155.	Micromonospora zingiberis PLAI 1-1	Micromonospora lacuserhaii CPCC 205547	86.09	80.83	25.3

1156.	Micromonospora zingiberis PLAI 1-1	Micromonospora rubida NEAU-HG-1	86.14	80.44	25.3
1157.	MMicromonospora solifontis PPF5-17	Micromonospora fiedleri MG-37	87.79	84.82	25.3
1158.	Micromonospora andamanensis NBRC 109075	Micromonospora coxensis DSM 45161	85.94	80.02	25.2
1159.	Micromonospora brunnea DSM 43814	Micromonospora sediminimaris NBRC 107745	86.04	80.62	25.2
1160.	Micromonospora endolithica DSM 44398	Micromonospora sediminimaris NBRC 107745	85.96	81.02	25.2
1161.	Micromonospora endophytica DSM 45430	Micromonospora lupini JCM 16031	85.83	79.96	25.2
1162.	Micromonospora endophytica DSM 45430	Micromonospora orduensis S2509	85.91	80.20	25.2
1163.	Micromonospora endophytica DSM 45430	Micromonospora rifamycinica DSM 44983	85.95	80.06	25.2
1164.	Micromonospora fiedleri MG-37	Micromonospora foliorum PSH25	85.87	79.79	25.2
1165.	Micromonospora fiedleri MG-37	Micromonospora halophytica DSM 43171	86.15	80.17	25.2
1166.	Micromonospora fiedleri MG-37	Micromonospora psammae CPCC 205556	86.21	80.85	25.2
1167.	Micromonospora fiedleri MG-37	Micromonospora purpureochromogenes DSM 43821	86.22	80.17	25.2
1168.	Micromonospora foliorum PSH25	Micromonospora palythoicola S2-005	85.70	80.53	25.2
1169.	Micromonospora foliorum PSH25	Micromonospora tulbaghiae DSM 45142	85.93	80.27	25.2
1170.	Micromonospora halophytica DSM 43171	Micromonospora chokoriensis DSM 45160	86.18	80.99	25.2
1171.	Micromonospora harpali NEAU-JC6	Micromonospora phaseoli CGMCC 4.7038	85.98	80.59	25.2
1172.	Micromonospora lacuserhaii CPCC 205547	Micromonospora phaseoli CGMCC 4.7038	86.07	81.05	25.2
1173.	Micromonospora noduli GUI43	Micromonospora schwarzwaldensis DSM 45708	85.99	80.40	25.2
1174.	Micromonospora oryzae DSM 102119	Micromonospora phaseoli CGMCC 4.7038	85.92	80.54	25.2
1175.	Micromonospora palythoicola S2-005	Micromonospora halophytica DSM 43171	86.01	80.53	25.2
1176.	Micromonospora profundi DSM 45981	Micromonospora antibiotica MMS20-R2-23	85.90	80.34	25.2
1177.	Micromonospora profundi DSM 45981	Micromonospora mirobrigensis DSM 44830	86.07	81.24	25.2
1178.	Micromonospora purpurea DSM 43036	Micromonospora foliorum PSH25	86.03	80.13	25.2
1179.	Micromonospora qiuiae NBRC 106684	Micromonospora rifamycinica DSM 44983	85.86	79.76	25.2
1180.	Micromonospora rifamycinica DSM 44983	Micromonospora chokoriensis DSM 45160	86.03	80.29	25.2

1181.	Micromonospora rubida NEAU-HG-1	Micromonospora fiedleri MG-37	85.95	79.64	25.2
1182.	Micromonospora saelicesensis DSM 44871	Micromonospora nigra DSM 43818	85.72	81.46	25.2
1183.	Micromonospora sediminimaris NBRC 107745	Micromonospora maritima DSM 45782	85.98	80.14	25.2
1184.	Micromonospora sonchi CGMCC 4.7312	Micromonospora mirobrigensis DSM 44830	86.00	80.63	25.2
1185.	Micromonospora taraxaci DSM 45885	Micromonospora halophytica DSM 43171	86.07	80.97	25.2
1186.	Micromonospora taraxaci DSM 45885	Micromonospora mirobrigensis DSM 44830	86.01	81.40	25.2
1187.	Micromonospora ureilytica DSM 101692	Micromonospora nigra DSM 43818	85.70	80.57	25.2
1188.	Micromonospora vinacea DSM 101695	Micromonospora nigra DSM 43818	85.82	80.89	25.2
1189.	Micromonospora violae DSM 45888	Micromonospora nigra DSM 43818	85.79	80.77	25.2
1190.	Micromonospora vulcania CGMCC 4.7144	Micromonospora fluminis A38	86.07	80.96	25.2
1191.	MMicromonospora solifontis PPF5-17	Micromonospora phaseoli CGMCC 4.7038	85.98	81.04	25.2
1192.	Micromonospora alfalfae MED01	Micromonospora nigra DSM 43818	85.61	80.79	25.1
1193.	Micromonospora antibiotica MMS20-R2-23	Micromonospora chokoriensis DSM 45160	86.03	80.35	25.1
1194.	Micromonospora arida LB32	Micromonospora schwarzwaldensis DSM 45708	86.00	80.53	25.1
1195.	Micromonospora arida LB32	Micromonospora sediminicola DSM 45794	85.93	80.61	25.1
1196.	Micromonospora brunnea DSM 43814	Micromonospora andamanensis NBRC 109075	86.08	80.71	25.1
1197.	Micromonospora cabrerizensis LAH09	Micromonospora nigra DSM 43818	85.72	80.61	25.1
1198.	Micromonospora endolithica DSM 44398	Micromonospora andamanensis NBRC 109075	85.98	80.64	25.1
1199.	Micromonospora endophytica DSM 45430	Micromonospora arida LB32	85.71	80.03	25.1
1200.	Micromonospora endophytica DSM 45430	Micromonospora mirobrigensis DSM 44830	86.06	80.08	25.1
1201.	Micromonospora endophytica DSM 45430	Micromonospora vulcania CGMCC 4.7144	85.86	80.04	25.1
1202.	Micromonospora fiedleri MG-37	Micromonospora rifamycinica DSM 44983	86.06	79.96	25.1
1203.	Micromonospora lupini JCM 16031	Micromonospora fluminis A38	86.07	80.86	25.1
1204.	Micromonospora nigra DSM 43818	Micromonospora fluminis A38	85.93	80.70	25.1
1205.	Micromonospora orduensis S2509	Micromonospora purpurea DSM 43036	86.03	80.40	25.1

1206.	Micromonospora orduensis S2509	Micromonospora qiuiae NBRC 106684	85.79	80.07	25.1
1207.	Micromonospora orduensis S2509	Micromonospora sonchi CGMCC 4.7312	85.84	80.57	25.1
1208.	Micromonospora orduensis S2509	Micromonospora tulbaghiae DSM 45142	86.06	80.53	25.1
1209.	Micromonospora palythoicola S2-005	Micromonospora psammae CPCC 205556	86.12	80.57	25.1
1210.	Micromonospora parathelypteridis DSM 103125	Micromonospora nigra DSM 43818	85.78	80.51	25.1
1211.	Micromonospora qiuiae NBRC 106684	Micromonospora mirobrigensis DSM 44830	85.94	80.41	25.1
1212.	Micromonospora robiginosa 28ISP2-46	Micromonospora nigra DSM 43818	85.98	80.77	25.1
1213.	Micromonospora rubida NEAU-HG-1	Micromonospora andamanensis NBRC 109075	85.85	79.67	25.1
1214.	Micromonospora salmantinae PSH03	Micromonospora schwarzwaldensis DSM 45708	86.02	80.44	25.1
1215.	Micromonospora schwarzwaldensis DSM 45708	Micromonospora saelicesensis DSM 44871	85.99	80.63	25.1
1216.	Micromonospora sonchi CGMCC 4.7312	Micromonospora vulcania CGMCC 4.7144	85.87	80.37	25.1
1217.	Micromonospora taraxaci DSM 45885	Micromonospora antibiotica MMS20-R2-23	85.97	80.15	25.1
1218.	Micromonospora taraxaci DSM 45885	Micromonospora rifamycinica DSM 44983	86.01	80.13	25.1
1219.	Micromonospora thermarum HSS6-12	Micromonospora lutea NBRC 106530	85.95	80.79	25.1
1220.	Micromonospora trifolii NIE79	Micromonospora nigra DSM 43818	85.77	80.45	25.1
1221.	Micromonospora ureilytica DSM 101692	Micromonospora schwarzwaldensis DSM 45708	85.99	80.30	25.1
1222.	Micromonospora violae DSM 45888	Micromonospora schwarzwaldensis DSM 45708	85.99	80.41	25.1
1223.	Micromonospora zingiberis PLAI 1-1	Micromonospora mirobrigensis DSM 44830	86.09	80.78	25.1
1224.	Micromonospora zingiberis PLAI 1-1	Micromonospora vulcania CGMCC 4.7144	85.91	80.42	25.1
1225.	MMicromonospora solifontis PPF5-17	Micromonospora palythoicola S2-005	86.03	80.37	25.1
1226.	Micromonospora acroterricola 5R2A7	Micromonospora andamanensis NBRC 109075	85.73	80.36	25
1227.	Micromonospora acroterricola 5R2A7	Micromonospora sediminimaris NBRC 107745	85.70	80.46	25
1228.	Micromonospora andamanensis NBRC 109075	Micromonospora foliorum PSH25	85.62	79.88	25
1229.	Micromonospora andamanensis NBRC 109075	Micromonospora maritima DSM 45782	85.89	80.12	25
1230.	Micromonospora antibiotica MMS20-R2-23	Micromonospora nigra DSM 43818	86.16	80.28	25

1231.	Micromonospora arida LB32	Micromonospora humi DSM 45647	85.95	80.44	25
1232.	Micromonospora arida LB32	Micromonospora robiginosa 28ISP2-46	85.97	80.44	25
1233.	Micromonospora arida LB32	Micromonospora sonchi CGMCC 4.7312	85.67	80.39	25
1234.	Micromonospora chalcea DSM 43026	Micromonospora lupini JCM 16031	86.02	80.74	25
1235.	Micromonospora endophytica DSM 45430	Micromonospora antibiotica MMS20-R2-23	85.90	80.06	25
1236.	Micromonospora endophytica DSM 45430	Micromonospora noduli GUI43	85.76	79.97	25
1237.	Micromonospora endophytica DSM 45430	Micromonospora sediminicola DSM 45794	86.03	80.14	25
1238.	Micromonospora fiedleri MG-37	Micromonospora lacuserhaii CPCC 205547	86.10	80.51	25
1239.	Micromonospora hortensis NIE111	Micromonospora nigra DSM 43818	85.74	80.51	25
1240.	Micromonospora hortensis NIE111	Micromonospora schwarzwaldensis DSM 45708	85.92	80.30	25
1241.	Micromonospora jinlongensis DSM 45876	Micromonospora nigra DSM 43818	85.74	80.49	25
1242.	Micromonospora nigra DSM 43818	Micromonospora tulbaghiae DSM 45142	85.91	80.44	25
1243.	Micromonospora noduli GUI43	Micromonospora sediminicola DSM 45794	85.93	80.50	25
1244.	Micromonospora orduensis S2509	Micromonospora marina PCU 269	85.98	81.04	25
1245.	Micromonospora palomenae DSM 102131	Micromonospora andamanensis NBRC 109075	86.04	80.85	25
1246.	Micromonospora palomenae DSM 102131	Micromonospora sediminimaris NBRC 107745	85.97	80.43	25
1247.	Micromonospora profundi DSM 45981	Micromonospora fluminis A38	85.91	80.84	25
1248.	Micromonospora profundi DSM 45981	Micromonospora purpurea DSM 43036	85.93	80.49	25
1249.	Micromonospora profundi DSM 45981	Micromonospora schwarzwaldensis DSM 45708	85.87	80.41	25
1250.	Micromonospora profundi DSM 45981	Micromonospora sediminicola DSM 45794	85.88	80.42	25
1251.	Micromonospora purpurea DSM 43036	Micromonospora lupini JCM 16031	85.97	80.16	25
1252.	Micromonospora qiuiae NBRC 106684	Micromonospora antibiotica MMS20-R2-23	85.80	79.48	25
1253.	Micromonospora qiuiae NBRC 106684	Micromonospora vulcania CGMCC 4.7144	85.73	79.98	25
1254.	Micromonospora robiginosa 28ISP2-46	Micromonospora ureilytica DSM 101692	85.92	80.05	25
1255.	Micromonospora rubida NEAU-HG-1	Micromonospora sediminimaris NBRC 107745	85.82	79.97	25

1256.	Micromonospora schwarzwaldensis DSM 45708	Micromonospora nigra DSM 43818	85.90	80.78	25
1257.	Micromonospora sediminimaris NBRC 107745	Micromonospora psammae CPCC 205556	85.97	80.33	25
1258.	Micromonospora sediminimaris NBRC 107745	Micromonospora purpureochromogenes DSM 43821	85.98	80.35	25
1259.	Micromonospora sonchi CGMCC 4.7312	Micromonospora antibiotica MMS20-R2-23	85.84	80.04	25
1260.	Micromonospora sonchi CGMCC 4.7312	Micromonospora lupini JCM 16031	85.70	80.19	25
1261.	Micromonospora sonchi CGMCC 4.7312	Micromonospora sediminicola DSM 45794	86.01	80.23	25
1262.	Micromonospora ureilytica DSM 101692	Micromonospora sediminicola DSM 45794	85.87	80.21	25
1263.	Micromonospora vinacea DSM 101695	Micromonospora schwarzwaldensis DSM 45708	86.01	80.43	25
1264.	Micromonospora vinacea DSM 101695	Micromonospora sediminicola DSM 45794	86.02	80.40	25
1265.	Micromonospora violae DSM 45888	Micromonospora robiginosa 28ISP2-46	85.94	80.35	25
1266.	Micromonospora violae DSM 45888	Micromonospora sediminicola DSM 45794	86.00	80.53	25
1267.	Micromonospora vulcania CGMCC 4.7144	Micromonospora phaseoli CGMCC 4.7038	85.75	80.39	25
1268.	Micromonospora zamorensis DSM 45600	Micromonospora nigra DSM 43818	85.74	80.50	25
1269.	Micromonospora zingiberis PLAI 1-1	Micromonospora oryzae DSM 102119	86.06	80.53	25
1270.	Micromonospora alfalfae MED01	Micromonospora schwarzwaldensis DSM 45708	85.91	80.26	24.9
1271.	Micromonospora alfalfae MED01	Micromonospora sediminicola DSM 45794	85.88	81.09	24.9
1272.	Micromonospora andamanensis NBRC 109075	Micromonospora halophytica DSM 43171	85.85	80.47	24.9
1273.	Micromonospora andamanensis NBRC 109075	Micromonospora purpureochromogenes DSM 43821	85.98	80.83	24.9
1274.	Micromonospora antibiotica MMS20-R2-23	Micromonospora phaseoli CGMCC 4.7038	85.76	80.07	24.9
1275.	Micromonospora arida LB32	Micromonospora fluminis A38	85.86	80.26	24.9
1276.	Micromonospora arida LB32	Micromonospora phaseoli CGMCC 4.7038	85.64	80.51	24.9
1277.	Micromonospora arida LB32	Micromonospora qiuiae NBRC 106684	85.50	79.98	24.9
1278.	Micromonospora aurantiaca ATCC 27029	Micromonospora lupini JCM 16031	86.01	80.28	24.9
1279.	Micromonospora aurantiaca ATCC 27029	Micromonospora nigra DSM 43818	86.02	80.57	24.9
1280.	Micromonospora aurantiaca ATCC 27029	Micromonospora vulcania CGMCC 4.7144	86.00	80.42	24.9

1281.	Micromonospora brunnea DSM 43814	Micromonospora lutea NBRC 106530	85.94	80.30	24.9
1282.	Micromonospora chalcea DSM 43026	Micromonospora nigra DSM 43818	85.99	80.54	24.9
1283.	Micromonospora chalcea DSM 43026	Micromonospora vulcania CGMCC 4.7144	86.03	80.50	24.9
1284.	Micromonospora chokoriensis DSM 45160	Micromonospora nigra DSM 43818	85.79	80.55	24.9
1285.	Micromonospora endolithica DSM 44398	Micromonospora lutea NBRC 106530	85.85	80.79	24.9
1286.	Micromonospora endophytica DSM 45430	Micromonospora cabrerizensis LAH09	85.77	80.01	24.9
1287.	Micromonospora endophytica DSM 45430	Micromonospora humi DSM 45647	85.94	79.95	24.9
1288.	Micromonospora endophytica DSM 45430	Micromonospora robiginosa 28ISP2-46	85.96	79.98	24.9
1289.	Micromonospora endophytica DSM 45430	Micromonospora ureilytica DSM 101692	85.75	79.80	24.9
1290.	Micromonospora endophytica DSM 45430	Micromonospora vinacea DSM 101695	85.79	79.91	24.9
1291.	Micromonospora fiedleri MG-37	Micromonospora mirobrigensis DSM 44830	86.04	80.44	24.9
1292.	Micromonospora fiedleri MG-37	Micromonospora oryzae DSM 102119	85.97	79.77	24.9
1293.	Micromonospora hortensis NIE111	Micromonospora humi DSM 45647	85.83	80.24	24.9
1294.	Micromonospora lupini JCM 16031	Micromonospora tulbaghiae DSM 45142	85.96	80.79	24.9
1295.	Micromonospora lutea NBRC 106530	Micromonospora coxensis DSM 45161	85.89	80.14	24.9
1296.	Micromonospora lutea NBRC 106530	Micromonospora halophytica DSM 43171	85.91	79.99	24.9
1297.	Micromonospora lutea NBRC 106530	Micromonospora maritima DSM 45782	85.85	79.83	24.9
1298.	Micromonospora mirobrigensis DSM 44830	Micromonospora phaseoli CGMCC 4.7038	85.94	80.64	24.9
1299.	Micromonospora noduli GUI43	Micromonospora humi DSM 45647	85.95	80.27	24.9
1300.	Micromonospora noduli GUI43	Micromonospora robiginosa 28ISP2-46	85.94	80.34	24.9
1301.	Micromonospora noduli GUI43	Micromonospora sonchi CGMCC 4.7312	85.64	80.38	24.9
1302.	Micromonospora orduensis S2509	Micromonospora fiedleri MG-37	85.78	80.08	24.9
1303.	Micromonospora orduensis S2509	Micromonospora phaseoli CGMCC 4.7038	85.71	80.64	24.9
1304.	Micromonospora profundi DSM 45981	Micromonospora nigra DSM 43818	85.72	80.70	24.9
1305.	Micromonospora profundi DSM 45981	Micromonospora robiginosa 28ISP2-46	85.91	80.41	24.9

1306.	Micromonospora qiuiae NBRC 106684	Micromonospora lupini JCM 16031	85.59	79.65	24.9
1307.	Micromonospora rifamycinica DSM 44983	Micromonospora phaseoli CGMCC 4.7038	85.74	80.16	24.9
1308.	Micromonospora robiginosa 28ISP2-46	Micromonospora hortensis NIE111	85.91	80.27	24.9
1309.	Micromonospora robiginosa 28ISP2-46	Micromonospora saelicesensis DSM 44871	85.97	80.56	24.9
1310.	Micromonospora robiginosa 28ISP2-46	Micromonospora salmantinae PSH03	85.94	80.40	24.9
1311.	Micromonospora robiginosa 28ISP2-46	Micromonospora trifolii NIE79	85.87	80.80	24.9
1312.	Micromonospora robiginosa 28ISP2-46	Micromonospora vinacea DSM 101695	85.99	80.96	24.9
1313.	Micromonospora salmantinae PSH03	Micromonospora humi DSM 45647	85.91	80.33	24.9
1314.	Micromonospora salmantinae PSH03	Micromonospora sediminicola DSM 45794	85.92	80.51	24.9
1315.	Micromonospora sediminicola DSM 45794	Micromonospora saelicesensis DSM 44871	85.94	80.61	24.9
1316.	Micromonospora sediminimaris NBRC 107745	Micromonospora coxensis DSM 45161	86.00	80.50	24.9
1317.	Micromonospora sediminimaris NBRC 107745	Micromonospora foliorum PSH25	85.73	80.32	24.9
1318.	Micromonospora sediminimaris NBRC 107745	Micromonospora halophytica DSM 43171	85.90	80.38	24.9
1319.	Micromonospora sediminimaris NBRC 107745	Micromonospora lacuserhaii CPCC 205547	85.86	80.42	24.9
1320.	Micromonospora trifolii NIE79	Micromonospora schwarzwaldensis DSM 45708	85.93	80.36	24.9
1321.	Micromonospora trifolii NIE79	Micromonospora sediminicola DSM 45794	85.90	80.36	24.9
1322.	Micromonospora ureilytica DSM 101692	Micromonospora humi DSM 45647	85.93	80.11	24.9
1323.	Micromonospora violae DSM 45888	Micromonospora fluminis A38	85.82	80.65	24.9
1324.	Micromonospora violae DSM 45888	Micromonospora humi DSM 45647	85.97	80.33	24.9
1325.	Micromonospora zingiberis PLAI 1-1	Micromonospora harpali NEAU-JC6	86.07	80.50	24.9
1326.	Micromonospora zingiberis PLAI 1-1	Micromonospora orduensis S2509	85.81	80.40	24.9
1327.	Micromonospora zingiberis PLAI 1-1	Micromonospora rifamycinica DSM 44983	86.00	80.21	24.9
1328.	Micromonospora alfalfae MED01	Micromonospora humi DSM 45647	85.82	80.75	24.8
1329.	Micromonospora andamanensis NBRC 109075	Micromonospora harpali NEAU-JC6	85.80	79.89	24.8
1330.	Micromonospora andamanensis NBRC 109075	Micromonospora oryzae DSM 102119	85.86	79.88	24.8

1331.	Micromonospora arida LB32	Micromonospora zingiberis PLAI 1-1	85.75	80.47	24.8
1332.	Micromonospora aurantiaca ATCC 27029	Micromonospora profundi DSM 45981	85.86	79.84	24.8
1333.	Micromonospora cabrerizensis LAH09	Micromonospora tulbaghiae DSM 45142	85.85	80.16	24.8
1334.	Micromonospora chalcea DSM 43026	Micromonospora profundi DSM 45981	85.91	80.36	24.8
1335.	Micromonospora chalcea DSM 43026	Micromonospora violae DSM 45888	85.86	80.14	24.8
1336.	Micromonospora endophytica DSM 45430	Micromonospora chokoriensis DSM 45160	85.67	79.91	24.8
1337.	Micromonospora endophytica DSM 45430	Micromonospora hortensis NIE111	85.71	79.89	24.8
1338.	Micromonospora endophytica DSM 45430	Micromonospora jinlongensis DSM 45876	85.74	79.95	24.8
1339.	Micromonospora endophytica DSM 45430	Micromonospora parathelypteridis DSM 103125	85.72	79.70	24.8
1340.	Micromonospora endophytica DSM 45430	Micromonospora profundi DSM 45981	85.59	79.70	24.8
1341.	Micromonospora endophytica DSM 45430	Micromonospora saelicesensis DSM 44871	85.76	80.03	24.8
1342.	Micromonospora endophytica DSM 45430	Micromonospora salmantinae PSH03	85.77	79.99	24.8
1343.	Micromonospora endophytica DSM 45430	Micromonospora schwarzwaldensis DSM 45708	85.96	79.90	24.8
1344.	Micromonospora endophytica DSM 45430	Micromonospora trifolii NIE79	85.72	79.87	24.8
1345.	Micromonospora endophytica DSM 45430	Micromonospora zamorensis DSM 45600	85.73	79.92	24.8
1346.	Micromonospora fiedleri MG-37	Micromonospora antibiotica MMS20-R2-23	85.86	79.64	24.8
1347.	Micromonospora fiedleri MG-37	Micromonospora harpali NEAU-JC6	85.98	79.77	24.8
1348.	Micromonospora fiedleri MG-37	Micromonospora vulcania CGMCC 4.7144	85.83	79.91	24.8
1349.	Micromonospora hortensis NIE111	Micromonospora sediminicola DSM 45794	85.83	80.38	24.8
1350.	Micromonospora humi DSM 45647	Micromonospora nigra DSM 43818	85.99	80.25	24.8
1351.	Micromonospora humi DSM 45647	Micromonospora saelicesensis DSM 44871	85.96	80.54	24.8
1352.	Micromonospora jinlongensis DSM 45876	Micromonospora sonchi CGMCC 4.7312	85.62	80.21	24.8
1353.	Micromonospora lupini JCM 16031	Micromonospora marina PCU 269	85.95	80.74	24.8
1354.	Micromonospora lupini JCM 16031	Micromonospora phaseoli CGMCC 4.7038	85.62	80.10	24.8
1355.	Micromonospora noduli GUI43	Micromonospora phaseoli CGMCC 4.7038	85.58	80.41	24.8

1356.	Micromonospora orduensis S2509	Micromonospora palythoicola S2-005	85.64	80.41	24.8
1357.	Micromonospora palomenae DSM 102131	Micromonospora lutea NBRC 106530	86.00	80.26	24.8
1358.	Micromonospora palythoicola S2-005	Micromonospora mirobrigensis DSM 44830	86.01	80.17	24.8
1359.	Micromonospora palythoicola S2-005	Micromonospora rifamycinica DSM 44983	85.85	80.26	24.8
1360.	Micromonospora parathelypteridis DSM 103125	Micromonospora schwarzwaldensis DSM 45708	85.90	80.14	24.8
1361.	Micromonospora parathelypteridis DSM 103125	Micromonospora sediminicola DSM 45794	85.89	80.31	24.8
1362.	Micromonospora profundi DSM 45981	Micromonospora humi DSM 45647	85.79	80.42	24.8
1363.	Micromonospora profundi DSM 45981	Micromonospora tulbaghiae DSM 45142	85.84	80.39	24.8
1364.	Micromonospora purpurea DSM 43036	Micromonospora nigra DSM 43818	85.97	80.49	24.8
1365.	Micromonospora purpurea DSM 43036	Micromonospora vulcania CGMCC 4.7144	85.92	80.31	24.8
1366.	Micromonospora qiuiae NBRC 106684	Micromonospora fluminis A38	85.88	79.56	24.8
1367.	Micromonospora qiuiae NBRC 106684	Micromonospora sediminicola DSM 45794	85.89	79.86	24.8
1368.	Micromonospora robiginosa 28ISP2-46	Micromonospora alfalfae MED01	85.87	80.88	24.8
1369.	Micromonospora robiginosa 28ISP2-46	Micromonospora parathelypteridis DSM 103125	85.88	80.05	24.8
1370.	Micromonospora robiginosa 28ISP2-46	Micromonospora sonchi CGMCC 4.7312	85.97	80.29	24.8
1371.	Micromonospora rubida NEAU-HG-1	Micromonospora lutea NBRC 106530	85.70	79.77	24.8
1372.	Micromonospora sediminimaris NBRC 107745	Micromonospora harpali NEAU-JC6	85.84	80.18	24.8
1373.	Micromonospora sediminimaris NBRC 107745	Micromonospora oryzae DSM 102119	85.85	80.20	24.8
1374.	Micromonospora sonchi CGMCC 4.7312	Micromonospora cabrerizensis LAH09	85.71	80.21	24.8
1375.	Micromonospora sonchi CGMCC 4.7312	Micromonospora chokoriensis DSM 45160	85.62	80.19	24.8
1376.	Micromonospora sonchi CGMCC 4.7312	Micromonospora fluminis A38	85.94	79.99	24.8
1377.	Micromonospora sonchi CGMCC 4.7312	Micromonospora schwarzwaldensis DSM 45708	85.93	79.99	24.8
1378.	Micromonospora sonchi CGMCC 4.7312	Micromonospora ureilytica DSM 101692	85.63	80.08	24.8
1379.	Micromonospora sonchi CGMCC 4.7312	Micromonospora vinacea DSM 101695	85.68	80.35	24.8
1380.	Micromonospora sonchi CGMCC 4.7312	Micromonospora zamorensis DSM 45600	85.60	80.22	24.8

1381.	Micromonospora taraxaci DSM 45885	Micromonospora nigra DSM 43818	85.74	80.48	24.8
1382.	Micromonospora taraxaci DSM 45885	Micromonospora sonchi CGMCC 4.7312	85.59	80.10	24.8
1383.	Micromonospora trifolii NIE79	Micromonospora humi DSM 45647	85.87	80.73	24.8
1384.	Micromonospora vinacea DSM 101695	Micromonospora fluminis A38	85.86	80.75	24.8
1385.	Micromonospora vinacea DSM 101695	Micromonospora humi DSM 45647	85.92	80.92	24.8
1386.	Micromonospora violae DSM 45888	Micromonospora qiuiae NBRC 106684	85.64	79.93	24.8
1387.	Micromonospora violae DSM 45888	Micromonospora sonchi CGMCC 4.7312	85.69	80.31	24.8
1388.	Micromonospora vulcania CGMCC 4.7144	Micromonospora tulbaghiae DSM 45142	85.96	80.94	24.8
1389.	Micromonospora zingiberis PLAI 1-1	Micromonospora antibiotica MMS20-R2-23	85.92	80.03	24.8
1390.	Micromonospora zingiberis PLAI 1-1	Micromonospora lupini JCM 16031	85.72	80.17	24.8
1391.	Micromonospora zingiberis PLAI 1-1	Micromonospora sediminicola DSM 45794	86.00	80.45	24.8
1392.	MMicromonospora solifontis PPF5-17	Micromonospora andamanensis NBRC 109075	87.46	84.43	24.8
1393.	MMicromonospora solifontis PPF5-17	Micromonospora sediminimaris NBRC 107745	85.86	80.64	24.8
1394.	Micromonospora acroterricola 5R2A7	Micromonospora lutea NBRC 106530	85.57	80.09	24.7
1395.	Micromonospora alfalfae MED01	Micromonospora fluminis A38	85.77	80.63	24.7
1396.	Micromonospora andamanensis NBRC 109075	Micromonospora lacuserhaii CPCC 205547	85.91	80.55	24.7
1397.	Micromonospora andamanensis NBRC 109075	Micromonospora psammae CPCC 205556	85.94	80.89	24.7
1398.	Micromonospora arida LB32	Micromonospora fiedleri MG-37	85.67	79.94	24.7
1399.	Micromonospora arida LB32	Micromonospora purpurea DSM 43036	85.84	80.06	24.7
1400.	Micromonospora aurantiaca ATCC 27029	Micromonospora arida LB32	85.80	80.17	24.7
1401.	Micromonospora cabrerizensis LAH09	Micromonospora humi DSM 45647	85.93	80.43	24.7
1402.	Micromonospora cabrerizensis LAH09	Micromonospora sediminicola DSM 45794	85.91	80.64	24.7
1403.	Micromonospora chalcea DSM 43026	Micromonospora arida LB32	85.89	80.18	24.7
1404.	Micromonospora chalcea DSM 43026	Micromonospora vinacea DSM 101695	85.87	80.05	24.7
1405.	Micromonospora endophytica DSM 45430	Micromonospora alfalfae MED01	85.67	79.85	24.7

1406.	Micromonospora endophytica DSM 45430	Micromonospora fluminis A38	85.92	79.80	24.7
1407.	Micromonospora endophytica DSM 45430	Micromonospora violae DSM 45888	85.75	79.87	24.7
1408.	Micromonospora fiedleri MG-37	Micromonospora lupini JCM 16031	85.72	79.66	24.7
1409.	Micromonospora fiedleri MG-37	Micromonospora sediminicola DSM 45794	85.98	79.84	24.7
1410.	Micromonospora jinlongensis DSM 45876	Micromonospora humi DSM 45647	85.92	80.42	24.7
1411.	Micromonospora jinlongensis DSM 45876	Micromonospora phaseoli CGMCC 4.7038	85.61	80.41	24.7
1412.	Micromonospora jinlongensis DSM 45876	Micromonospora schwarzwaldensis DSM 45708	85.98	80.43	24.7
1413.	Micromonospora lutea NBRC 106530	Micromonospora foliorum PSH25	85.57	79.94	24.7
1414.	Micromonospora lutea NBRC 106530	Micromonospora oryzae DSM 102119	85.74	79.85	24.7
1415.	Micromonospora marina PCU 269	Micromonospora nigra DSM 43818	85.92	80.78	24.7
1416.	Micromonospora marina PCU 269	Micromonospora vulcania CGMCC 4.7144	85.93	80.85	24.7
1417.	Micromonospora noduli GUI43	Micromonospora fluminis A38	85.85	80.70	24.7
1418.	Micromonospora noduli GUI43	Micromonospora qiuiae NBRC 106684	85.52	79.88	24.7
1419.	Micromonospora palythoicola S2-005	Micromonospora vulcania CGMCC 4.7144	85.71	80.20	24.7
1420.	Micromonospora parathelypteridis DSM 103125	Micromonospora humi DSM 45647	85.85	80.08	24.7
1421.	Micromonospora parathelypteridis DSM 103125	Micromonospora sonchi CGMCC 4.7312	85.64	80.07	24.7
1422.	Micromonospora qiuiae NBRC 106684	Micromonospora cabrerizensis LAH09	85.57	79.25	24.7
1423.	Micromonospora qiuiae NBRC 106684	Micromonospora schwarzwaldensis DSM 45708	85.92	79.61	24.7
1424.	Micromonospora robiginosa 28ISP2-46	Micromonospora cabrerizensis LAH09	85.92	80.41	24.7
1425.	Micromonospora saelicesensis DSM 44871	Micromonospora fluminis A38	85.85	81.00	24.7
1426.	Micromonospora saelicesensis DSM 44871	Micromonospora phaseoli CGMCC 4.7038	85.57	80.51	24.7
1427.	Micromonospora salmantinae PSH03	Micromonospora fluminis A38	85.84	80.06	24.7
1428.	Micromonospora schwarzwaldensis DSM 45708	Micromonospora chokoriensis DSM 45160	85.94	80.22	24.7
1429.	Micromonospora schwarzwaldensis DSM 45708	Micromonospora zamorensis DSM 45600	85.98	80.43	24.7
1430.	Micromonospora sediminicola DSM 45794	Micromonospora chokoriensis DSM 45160	86.01	80.50	24.7

1431.	Micromonospora sediminicola DSM 45794	Micromonospora zamorensis DSM 45600	85.97	80.47	24.7
1432.	Micromonospora sonchi CGMCC 4.7312	Micromonospora hortensis NIE111	85.59	80.06	24.7
1433.	Micromonospora sonchi CGMCC 4.7312	Micromonospora humi DSM 45647	85.89	80.20	24.7
1434.	Micromonospora sonchi CGMCC 4.7312	Micromonospora saelicesensis DSM 44871	85.63	80.40	24.7
1435.	Micromonospora sonchi CGMCC 4.7312	Micromonospora salmantinae PSH03	85.64	80.21	24.7
1436.	Micromonospora sonchi CGMCC 4.7312	Micromonospora trifolii NIE79	85.58	80.15	24.7
1437.	Micromonospora ureilytica DSM 101692	Micromonospora fluminis A38	85.77	80.45	24.7
1438.	Micromonospora ureilytica DSM 101692	Micromonospora qiuiae NBRC 106684	85.48	79.73	24.7
1439.	Micromonospora vinacea DSM 101695	Micromonospora phaseoli CGMCC 4.7038	85.64	80.43	24.7
1440.	Micromonospora vinacea DSM 101695	Micromonospora qiuiae NBRC 106684	85.55	79.89	24.7
1441.	Micromonospora violae DSM 45888	Micromonospora purpurea DSM 43036	85.81	79.94	24.7
1442.	Micromonospora violae DSM 45888	Micromonospora zingiberis PLAI 1-1	85.67	80.20	24.7
1443.	Micromonospora zamorensis DSM 45600	Micromonospora phaseoli CGMCC 4.7038	85.67	80.37	24.7
1444.	Micromonospora zingiberis PLAI 1-1	Micromonospora humi DSM 45647	85.90	80.22	24.7
1445.	Micromonospora zingiberis PLAI 1-1	Micromonospora vinacea DSM 101695	85.68	80.28	24.7
1446.	Micromonospora antibiotica MMS20-R2-23	Micromonospora palythoicola S2-005	85.78	80.13	24.6
1447.	Micromonospora arida LB32	Micromonospora marina PCU 269	85.75	81.00	24.6
1448.	Micromonospora arida LB32	Micromonospora palythoicola S2-005	85.45	80.36	24.6
1449.	Micromonospora arida LB32	Micromonospora tulbaghiae DSM 45142	85.79	80.84	24.6
1450.	Micromonospora aurantiaca ATCC 27029	Micromonospora noduli GUI43	85.78	80.05	24.6
1451.	Micromonospora aurantiaca ATCC 27029	Micromonospora qiuiae NBRC 106684	85.79	79.59	24.6
1452.	Micromonospora aurantiaca ATCC 27029	Micromonospora salmantinae PSH03	85.84	79.99	24.6
1453.	Micromonospora aurantiaca ATCC 27029	Micromonospora sonchi CGMCC 4.7312	85.80	79.99	24.6
1454.	Micromonospora aurantiaca ATCC 27029	Micromonospora violae DSM 45888	85.86	80.00	24.6
1455.	Micromonospora cabrerizensis LAH09	Micromonospora phaseoli CGMCC 4.7038	85.63	80.42	24.6

1456.	Micromonospora cabrerizensis LAH09	Micromonospora schwarzwaldensis DSM 45708	85.93	80.48	24.6
1457.	Micromonospora chalcea DSM 43026	Micromonospora alfalfae MED01	85.80	80.51	24.6
1458.	Micromonospora chalcea DSM 43026	Micromonospora endophytica DSM 45430	85.95	79.78	24.6
1459.	Micromonospora chalcea DSM 43026	Micromonospora noduli GUI43	85.87	80.20	24.6
1460.	Micromonospora chalcea DSM 43026	Micromonospora qiuiae NBRC 106684	85.86	79.65	24.6
1461.	Micromonospora chalcea DSM 43026	Micromonospora salmantinae PSH03	85.85	80.05	24.6
1462.	Micromonospora chalcea DSM 43026	Micromonospora sonchi CGMCC 4.7312	85.90	79.72	24.6
1463.	Micromonospora chalcea DSM 43026	Micromonospora trifolii NIE79	85.77	80.01	24.6
1464.	Micromonospora chalcea DSM 43026	Micromonospora ureilytica DSM 101692	85.81	79.93	24.6
1465.	Micromonospora chokoriensis DSM 45160	Micromonospora phaseoli CGMCC 4.7038	85.56	80.40	24.6
1466.	Micromonospora chokoriensis DSM 45160	Micromonospora tulbaghiae DSM 45142	85.80	80.18	24.6
1467.	Micromonospora endophytica DSM 45430	Micromonospora taraxaci DSM 45885	85.60	79.84	24.6
1468.	Micromonospora fiedleri MG-37	Micromonospora chokoriensis DSM 45160	85.66	79.54	24.6
1469.	Micromonospora fiedleri MG-37	Micromonospora fluminis A38	86.01	79.84	24.6
1470.	Micromonospora hortensis NIE111	Micromonospora phaseoli CGMCC 4.7038	85.53	80.09	24.6
1471.	Micromonospora humi DSM 45647	Micromonospora zamorensis DSM 45600	85.90	80.26	24.6
1472.	Micromonospora jinlongensis DSM 45876	Micromonospora qiuiae NBRC 106684	85.56	79.44	24.6
1473.	Micromonospora jinlongensis DSM 45876	Micromonospora robiginosa 28ISP2-46	85.96	80.41	24.6
1474.	Micromonospora jinlongensis DSM 45876	Micromonospora sediminicola DSM 45794	85.95	80.50	24.6
1475.	Micromonospora lutea NBRC 106530	Micromonospora harpali NEAU-JC6	85.74	79.92	24.6
1476.	Micromonospora lutea NBRC 106530	Micromonospora lacuserhaii CPCC 205547	85.77	80.09	24.6
1477.	Micromonospora lutea NBRC 106530	Micromonospora purpureochromogenes DSM 43821	85.90	80.13	24.6
1478.	Micromonospora noduli GUI43	Micromonospora purpurea DSM 43036	85.80	80.05	24.6
1479.	Micromonospora noduli GUI43	Micromonospora zingiberis PLAI 1-1	85.65	80.35	24.6
1480.	Micromonospora palythoicola S2-005	Micromonospora humi DSM 45647	85.87	80.24	24.6

1481.	Micromonospora profundi DSM 45981	Micromonospora marina PCU 269	85.80	80.77	24.6
1482.	Micromonospora purpurea DSM 43036	Micromonospora qiuiae NBRC 106684	85.93	79.59	24.6
1483.	Micromonospora purpurea DSM 43036	Micromonospora sonchi CGMCC 4.7312	85.93	79.69	24.6
1484.	Micromonospora purpurea DSM 43036	Micromonospora vinacea DSM 101695	85.86	79.99	24.6
1485.	Micromonospora qiuiae NBRC 106684	Micromonospora chokoriensis DSM 45160	85.50	79.38	24.6
1486.	Micromonospora qiuiae NBRC 106684	Micromonospora humi DSM 45647	85.85	79.63	24.6
1487.	Micromonospora qiuiae NBRC 106684	Micromonospora saelicesensis DSM 44871	85.53	80.09	24.6
1488.	Micromonospora qiuiae NBRC 106684	Micromonospora salmantinae PSH03	85.53	79.83	24.6
1489.	Micromonospora qiuiae NBRC 106684	Micromonospora tulbaghiae DSM 45142	85.83	79.73	24.6
1490.	Micromonospora qiuiae NBRC 106684	Micromonospora zamorensis DSM 45600	85.57	79.65	24.6
1491.	Micromonospora robiginosa 28ISP2-46	Micromonospora palythoicola S2-005	85.85	80.14	24.6
1492.	Micromonospora robiginosa 28ISP2-46	Micromonospora qiuiae NBRC 106684	85.86	79.65	24.6
1493.	Micromonospora robiginosa 28ISP2-46	Micromonospora zamorensis DSM 45600	85.94	80.28	24.6
1494.	Micromonospora salmantinae PSH03	Micromonospora phaseoli CGMCC 4.7038	85.57	80.30	24.6
1495.	Micromonospora schwarzwaldensis DSM 45708	Micromonospora phaseoli CGMCC 4.7038	85.82	80.05	24.6
1496.	Micromonospora sediminicola DSM 45794	Micromonospora phaseoli CGMCC 4.7038	85.84	80.44	24.6
1497.	Micromonospora sediminimaris NBRC 107745	Micromonospora mirobrigensis DSM 44830	85.89	79.83	24.6
1498.	Micromonospora sonchi CGMCC 4.7312	Micromonospora alfalfae MED01	85.53	80.12	24.6
1499.	Micromonospora sonchi CGMCC 4.7312	Micromonospora tulbaghiae DSM 45142	85.87	79.84	24.6
1500.	Micromonospora taraxaci DSM 45885	Micromonospora sediminicola DSM 45794	85.93	80.25	24.6
1501.	Micromonospora trifolii NIE79	Micromonospora fluminis A38	85.77	80.56	24.6
1502.	Micromonospora trifolii NIE79	Micromonospora phaseoli CGMCC 4.7038	85.58	80.22	24.6
1503.	Micromonospora ureilytica DSM 101692	Micromonospora phaseoli CGMCC 4.7038	85.58	80.24	24.6
1504.	Micromonospora violae DSM 45888	Micromonospora phaseoli CGMCC 4.7038	85.59	80.34	24.6
1505.	Micromonospora violae DSM 45888	Micromonospora tulbaghiae DSM 45142	85.78	80.68	24.6

1506.	Micromonospora zingiberis PLAI 1-1	Micromonospora alfalfae MED01	85.59	80.22	24.6
1507.	Micromonospora zingiberis PLAI 1-1	Micromonospora cabrerizensis LAH09	85.71	80.27	24.6
1508.	Micromonospora zingiberis PLAI 1-1	Micromonospora fluminis A38	85.94	80.16	24.6
1509.	Micromonospora zingiberis PLAI 1-1	Micromonospora jinlongensis DSM 45876	85.72	80.16	24.6
1510.	Micromonospora zingiberis PLAI 1-1	Micromonospora parathelypteridis DSM 103125	85.72	80.15	24.6
1511.	Micromonospora zingiberis PLAI 1-1	Micromonospora saelicesensis DSM 44871	85.68	80.38	24.6
1512.	Micromonospora zingiberis PLAI 1-1	Micromonospora salmantinae PSH03	85.69	80.16	24.6
1513.	Micromonospora zingiberis PLAI 1-1	Micromonospora ureilytica DSM 101692	85.68	80.06	24.6
1514.	Micromonospora zingiberis PLAI 1-1	Micromonospora zamorensis DSM 45600	85.67	80.07	24.6
1515.	MMicromonospora solifontis PPF5-17	Micromonospora lutea NBRC 106530	87.44	84.51	24.6
1516.	Micromonospora alfalfae MED01	Micromonospora phaseoli CGMCC 4.7038	85.49	80.26	24.5
1517.	Micromonospora andamanensis NBRC 109075	Micromonospora mirobrigensis DSM 44830	85.82	80.43	24.5
1518.	Micromonospora andamanensis NBRC 109075	Micromonospora rifamycinica DSM 44983	85.66	79.46	24.5
1519.	Micromonospora andamanensis NBRC 109075	Micromonospora vulcania CGMCC 4.7144	85.55	79.59	24.5
1520.	Micromonospora aurantiaca ATCC 27029	Micromonospora alfalfae MED01	85.74	79.83	24.5
1521.	Micromonospora aurantiaca ATCC 27029	Micromonospora endophytica DSM 45430	85.95	79.82	24.5
1522.	Micromonospora aurantiaca ATCC 27029	Micromonospora hortensis NIE111	85.82	79.84	24.5
1523.	Micromonospora aurantiaca ATCC 27029	Micromonospora parathelypteridis DSM 103125	85.75	79.71	24.5
1524.	Micromonospora aurantiaca ATCC 27029	Micromonospora saelicesensis DSM 44871	85.79	80.24	24.5
1525.	Micromonospora aurantiaca ATCC 27029	Micromonospora ureilytica DSM 101692	85.78	79.80	24.5
1526.	Micromonospora aurantiaca ATCC 27029	Micromonospora vinacea DSM 101695	85.86	80.00	24.5
1527.	Micromonospora cabrerizensis LAH09	Micromonospora fluminis A38	85.84	80.51	24.5
1528.	Micromonospora chalcea DSM 43026	Micromonospora cabrerizensis LAH09	85.88	80.10	24.5
1529.	Micromonospora chalcea DSM 43026	Micromonospora hortensis NIE111	85.84	79.96	24.5
1530.	Micromonospora chalcea DSM 43026	Micromonospora saelicesensis DSM 44871	85.87	80.31	24.5

1531.	Micromonospora chokoriensis DSM 45160	Micromonospora fluminis A38	85.87	80.43	24.5
1532.	Micromonospora endophytica DSM 45430	Micromonospora purpurea DSM 43036	85.90	79.68	24.5
1533.	Micromonospora endophytica DSM 45430	Micromonospora tulbaghiae DSM 45142	85.86	79.73	24.5
1534.	Micromonospora fiedleri MG-37	Micromonospora cabrerizensis LAH09	85.61	79.42	24.5
1535.	Micromonospora fiedleri MG-37	Micromonospora saelicesensis DSM 44871	85.63	80.06	24.5
1536.	Micromonospora fiedleri MG-37	Micromonospora salmantinae PSH03	85.66	80.00	24.5
1537.	Micromonospora fiedleri MG-37	Micromonospora schwarzwaldensis DSM 45708	85.85	79.55	24.5
1538.	Micromonospora fiedleri MG-37	Micromonospora zamorensis DSM 45600	85.64	79.74	24.5
1539.	Micromonospora hortensis NIE111	Micromonospora fluminis A38	85.76	79.98	24.5
1540.	Micromonospora humi DSM 45647	Micromonospora chokoriensis DSM 45160	85.84	80.21	24.5
1541.	Micromonospora lutea NBRC 106530	Micromonospora psammae CPCC 205556	85.84	80.11	24.5
1542.	Micromonospora marina PCU 269	Micromonospora saelicesensis DSM 44871	85.72	81.19	24.5
1543.	Micromonospora noduli GUI43	Micromonospora fiedleri MG-37	85.63	79.89	24.5
1544.	Micromonospora noduli GUI43	Micromonospora marina PCU 269	85.72	80.80	24.5
1545.	Micromonospora noduli GUI43	Micromonospora tulbaghiae DSM 45142	85.76	80.69	24.5
1546.	Micromonospora orduensis S2509	Micromonospora andamanensis NBRC 109075	85.56	80.07	24.5
1547.	Micromonospora orduensis S2509	Micromonospora sediminimaris NBRC 107745	85.56	80.16	24.5
1548.	Micromonospora palythoicola S2-005	Micromonospora lupini JCM 16031	85.60	80.23	24.5
1549.	Micromonospora palythoicola S2-005	Micromonospora sediminicola DSM 45794	85.93	80.29	24.5
1550.	Micromonospora parathelypteridis DSM 103125	Micromonospora fluminis A38	85.76	80.35	24.5
1551.	Micromonospora parathelypteridis DSM 103125	Micromonospora palythoicola S2-005	85.55	80.03	24.5
1552.	Micromonospora parathelypteridis DSM 103125	Micromonospora phaseoli CGMCC 4.7038	85.58	80.19	24.5
1553.	Micromonospora parathelypteridis DSM 103125	Micromonospora qiuiae NBRC 106684	85.63	79.56	24.5
1554.	Micromonospora profundi DSM 45981	Micromonospora qiuiae NBRC 106684	85.46	79.71	24.5
1555.	Micromonospora purpurea DSM 43036	Micromonospora salmantinae PSH03	85.83	79.94	24.5

1556.	Micromonospora purpurea DSM 43036	Micromonospora ureilytica DSM 101692	85.77	79.88	24.5
1557.	Micromonospora qiuiae NBRC 106684	Micromonospora alfalfae MED01	85.48	79.89	24.5
1558.	Micromonospora qiuiae NBRC 106684	Micromonospora hortensis NIE111	85.50	79.68	24.5
1559.	Micromonospora qiuiae NBRC 106684	Micromonospora marina PCU 269	85.80	79.68	24.5
1560.	Micromonospora qiuiae NBRC 106684	Micromonospora trifolii NIE79	85.45	79.64	24.5
1561.	Micromonospora robiginosa 28ISP2-46	Micromonospora chokoriensis DSM 45160	85.90	80.28	24.5
1562.	Micromonospora saelicesensis DSM 44871	Micromonospora tulbaghiae DSM 45142	85.76	80.97	24.5
1563.	Micromonospora salmantinae PSH03	Micromonospora marina PCU 269	85.75	80.82	24.5
1564.	Micromonospora salmantinae PSH03	Micromonospora palythoicola S2-005	85.58	80.37	24.5
1565.	Micromonospora salmantinae PSH03	Micromonospora tulbaghiae DSM 45142	85.77	80.04	24.5
1566.	Micromonospora sediminimaris NBRC 107745	Micromonospora rifamycinica DSM 44983	85.70	79.86	24.5
1567.	Micromonospora sediminimaris NBRC 107745	Micromonospora vulcania CGMCC 4.7144	85.63	80.02	24.5
1568.	Micromonospora sonchi CGMCC 4.7312	Micromonospora marina PCU 269	85.82	80.05	24.5
1569.	Micromonospora taraxaci DSM 45885	Micromonospora humi DSM 45647	85.76	79.98	24.5
1570.	Micromonospora taraxaci DSM 45885	Micromonospora phaseoli CGMCC 4.7038	85.52	80.39	24.5
1571.	Micromonospora taraxaci DSM 45885	Micromonospora qiuiae NBRC 106684	85.54	79.67	24.5
1572.	Micromonospora taraxaci DSM 45885	Micromonospora schwarzwaldensis DSM 45708	85.88	80.10	24.5
1573.	Micromonospora ureilytica DSM 101692	Micromonospora fiedleri MG-37	85.58	79.73	24.5
1574.	Micromonospora vinacea DSM 101695	Micromonospora fiedleri MG-37	85.66	80.02	24.5
1575.	Micromonospora vinacea DSM 101695	Micromonospora marina PCU 269	85.74	80.84	24.5
1576.	Micromonospora vinacea DSM 101695	Micromonospora tulbaghiae DSM 45142	85.76	80.68	24.5
1577.	Micromonospora violae DSM 45888	Micromonospora fiedleri MG-37	85.63	79.86	24.5
1578.	Micromonospora violae DSM 45888	Micromonospora marina PCU 269	85.80	80.70	24.5
1579.	Micromonospora zamorensis DSM 45600	Micromonospora tulbaghiae DSM 45142	85.82	80.83	24.5
1580.	Micromonospora zingiberis PLAI 1-1	Micromonospora chokoriensis DSM 45160	85.67	80.14	24.5

1581.	Micromonospora zingiberis PLAI 1-1	Micromonospora hortensis NIE111	85.63	80.12	24.5
1582.	Micromonospora zingiberis PLAI 1-1	Micromonospora robiginosa 28ISP2-46	85.90	80.19	24.5
1583.	Micromonospora zingiberis PLAI 1-1	Micromonospora schwarzwaldensis DSM 45708	85.91	80.18	24.5
1584.	Micromonospora zingiberis PLAI 1-1	Micromonospora trifolii NIE79	85.60	80.12	24.5
1585.	Micromonospora alfalfae MED01	Micromonospora marina PCU 269	85.68	80.71	24.4
1586.	Micromonospora alfalfae MED01	Micromonospora tulbaghiae DSM 45142	85.65	80.65	24.4
1587.	Micromonospora andamanensis NBRC 109075	Micromonospora antibiotica MMS20-R2-23	85.64	79.64	24.4
1588.	Micromonospora arida LB32	Micromonospora sediminimaris NBRC 107745	85.43	80.10	24.4
1589.	Micromonospora aurantiaca ATCC 27029	Micromonospora cabrerizensis LAH09	85.84	80.01	24.4
1590.	Micromonospora aurantiaca ATCC 27029	Micromonospora trifolii NIE79	85.75	79.95	24.4
1591.	Micromonospora cabrerizensis LAH09	Micromonospora marina PCU 269	85.88	80.69	24.4
1592.	Micromonospora cabrerizensis LAH09	Micromonospora palythoicola S2-005	85.53	80.35	24.4
1593.	Micromonospora chalcea DSM 43026	Micromonospora fiedleri MG-37	85.96	79.61	24.4
1594.	Micromonospora chalcea DSM 43026	Micromonospora parathelypteridis DSM 103125	85.76	79.80	24.4
1595.	Micromonospora chalcea DSM 43026	Micromonospora zamorensis DSM 45600	85.85	80.08	24.4
1596.	Micromonospora chalcea DSM 43026	Micromonospora zingiberis PLAI 1-1	85.91	80.03	24.4
1597.	Micromonospora endophytica DSM 45430	Micromonospora marina PCU 269	85.93	80.11	24.4
1598.	Micromonospora fiedleri MG-37	Micromonospora alfalfae MED01	85.57	79.88	24.4
1599.	Micromonospora fiedleri MG-37	Micromonospora humi DSM 45647	85.86	79.66	24.4
1600.	Micromonospora fiedleri MG-37	Micromonospora trifolii NIE79	85.58	79.78	24.4
1601.	Micromonospora hortensis NIE111	Micromonospora marina PCU 269	85.70	80.52	24.4
1602.	Micromonospora hortensis NIE111	Micromonospora tulbaghiae DSM 45142	85.73	79.93	24.4
1603.	Micromonospora humi DSM 45647	Micromonospora phaseoli CGMCC 4.7038	85.75	80.08	24.4
1604.	Micromonospora jinlongensis DSM 45876	Micromonospora fiedleri MG-37	85.64	79.50	24.4
1605.	Micromonospora jinlongensis DSM 45876	Micromonospora tulbaghiae DSM 45142	85.80	80.16	24.4

1606.	Micromonospora lutea NBRC 106530	Micromonospora rifamycinica DSM 44983	85.66	79.70	24.4
1607.	Micromonospora noduli GUI43	Micromonospora palythoicola S2-005	85.46	80.14	24.4
1608.	Micromonospora palythoicola S2-005	Micromonospora chokoriensis DSM 45160	85.58	80.33	24.4
1609.	Micromonospora palythoicola S2-005	Micromonospora fluminis A38	85.75	80.01	24.4
1610.	Micromonospora parathelypteridis DSM 103125	Micromonospora fiedleri MG-37	85.61	79.77	24.4
1611.	Micromonospora parathelypteridis DSM 103125	Micromonospora tulbaghiae DSM 45142	85.79	79.77	24.4
1612.	Micromonospora phaseoli CGMCC 4.7038	Micromonospora fluminis A38	85.72	79.94	24.4
1613.	Micromonospora profundi DSM 45981	Micromonospora fiedleri MG-37	85.57	79.67	24.4
1614.	Micromonospora profundi DSM 45981	Micromonospora sonchi CGMCC 4.7312	85.54	79.95	24.4
1615.	Micromonospora purpurea DSM 43036	Micromonospora alfalfae MED01	85.73	80.36	24.4
1616.	Micromonospora purpurea DSM 43036	Micromonospora cabrerizensis LAH09	85.84	80.07	24.4
1617.	Micromonospora purpurea DSM 43036	Micromonospora hortensis NIE111	85.79	79.91	24.4
1618.	Micromonospora purpurea DSM 43036	Micromonospora saelicesensis DSM 44871	85.80	80.21	24.4
1619.	Micromonospora purpurea DSM 43036	Micromonospora trifolii NIE79	85.77	79.90	24.4
1620.	Micromonospora robiginosa 28ISP2-46	Micromonospora fiedleri MG-37	85.90	79.69	24.4
1621.	Micromonospora robiginosa 28ISP2-46	Micromonospora phaseoli CGMCC 4.7038	85.74	80.10	24.4
1622.	Micromonospora sediminimaris NBRC 107745	Micromonospora antibiotica MMS20-R2-23	85.58	79.84	24.4
1623.	Micromonospora sediminimaris NBRC 107745	Micromonospora lupini JCM 16031	85.55	79.97	24.4
1624.	Micromonospora sediminimaris NBRC 107745	Micromonospora sediminicola DSM 45794	85.80	79.87	24.4
1625.	Micromonospora taraxaci DSM 45885	Micromonospora robiginosa 28ISP2-46	85.81	79.95	24.4
1626.	Micromonospora taraxaci DSM 45885	Micromonospora tulbaghiae DSM 45142	85.73	80.58	24.4
1627.	Micromonospora trifolii NIE79	Micromonospora palythoicola S2-005	85.46	80.22	24.4
1628.	Micromonospora trifolii NIE79	Micromonospora tulbaghiae DSM 45142	85.73	80.70	24.4
1629.	Micromonospora ureilytica DSM 101692	Micromonospora marina PCU 269	85.74	80.36	24.4
1630.	Micromonospora ureilytica DSM 101692	Micromonospora tulbaghiae DSM 45142	85.75	80.45	24.4

1631.	Micromonospora vinacea DSM 101695	Micromonospora palythoicola S2-005	85.60	80.37	24.4
1632.	Micromonospora violae DSM 45888	Micromonospora palythoicola S2-005	85.56	80.18	24.4
1633.	Micromonospora zamorensis DSM 45600	Micromonospora fluminis A38	85.83	80.61	24.4
1634.	Micromonospora zingiberis PLAI 1-1	Micromonospora marina PCU 269	85.80	79.95	24.4
1635.	Micromonospora zingiberis PLAI 1-1	Micromonospora taraxaci DSM 45885	85.64	80.11	24.4
1636.	Micromonospora alfalfae MED01	Micromonospora palythoicola S2-005	85.38	80.15	24.3
1637.	Micromonospora andamanensis NBRC 109075	Micromonospora cabrerizensis LAH09	85.48	79.69	24.3
1638.	Micromonospora andamanensis NBRC 109075	Micromonospora fluminis A38	85.63	79.81	24.3
1639.	Micromonospora andamanensis NBRC 109075	Micromonospora hortensis NIE111	85.37	79.59	24.3
1640.	Micromonospora andamanensis NBRC 109075	Micromonospora lupini JCM 16031	85.55	79.46	24.3
1641.	Micromonospora aurantiaca ATCC 27029	Micromonospora chokoriensis DSM 45160	85.88	80.04	24.3
1642.	Micromonospora aurantiaca ATCC 27029	Micromonospora fiedleri MG-37	85.87	79.53	24.3
1643.	Micromonospora aurantiaca ATCC 27029	Micromonospora zamorensis DSM 45600	85.85	80.07	24.3
1644.	Micromonospora chalcea DSM 43026	Micromonospora chokoriensis DSM 45160	85.88	80.09	24.3
1645.	Micromonospora chalcea DSM 43026	Micromonospora jinlongensis DSM 45876	85.81	80.09	24.3
1646.	Micromonospora chalcea DSM 43026	Micromonospora palythoicola S2-005	85.74	79.87	24.3
1647.	Micromonospora fiedleri MG-37	Micromonospora hortensis NIE111	85.58	79.69	24.3
1648.	Micromonospora hortensis NIE111	Micromonospora palythoicola S2-005	85.46	80.17	24.3
1649.	Micromonospora jinlongensis DSM 45876	Micromonospora fluminis A38	85.85	80.13	24.3
1650.	Micromonospora jinlongensis DSM 45876	Micromonospora palythoicola S2-005	85.53	80.28	24.3
1651.	Micromonospora lutea NBRC 106530	Micromonospora antibiotica MMS20-R2-23	85.55	79.54	24.3
1652.	Micromonospora lutea NBRC 106530	Micromonospora mirobrigensis DSM 44830	85.76	79.61	24.3
1653.	Micromonospora lutea NBRC 106530	Micromonospora vulcania CGMCC 4.7144	85.51	79.73	24.3
1654.	Micromonospora noduli GUI43	Micromonospora andamanensis NBRC 109075	85.38	79.49	24.3
1655.	Micromonospora palythoicola S2-005	Micromonospora saelicesensis DSM 44871	85.51	80.41	24.3

1656.	Micromonospora palythoicola S2-005	Micromonospora schwarzwaldensis DSM 45708	85.78	80.10	24.3
1657.	Micromonospora palythoicola S2-005	Micromonospora zamorensis DSM 45600	85.56	80.12	24.3
1658.	Micromonospora parathelypteridis DSM 103125	Micromonospora marina PCU 269	85.69	80.37	24.3
1659.	Micromonospora parathelypteridis DSM 103125	Micromonospora purpurea DSM 43036	85.75	79.71	24.3
1660.	Micromonospora profundi DSM 45981	Micromonospora phaseoli CGMCC 4.7038	85.38	80.00	24.3
1661.	Micromonospora purpurea DSM 43036	Micromonospora chokoriensis DSM 45160	85.84	79.96	24.3
1662.	Micromonospora purpurea DSM 43036	Micromonospora fiedleri MG-37	85.94	79.56	24.3
1663.	Micromonospora sediminimaris NBRC 107745	Micromonospora humi DSM 45647	85.71	79.80	24.3
1664.	Micromonospora taraxaci DSM 45885	Micromonospora fiedleri MG-37	85.61	79.84	24.3
1665.	Micromonospora taraxaci DSM 45885	Micromonospora fluminis A38	85.79	80.39	24.3
1666.	Micromonospora trifolii NIE79	Micromonospora marina PCU 269	85.69	80.53	24.3
1667.	Micromonospora vinacea DSM 101695	Micromonospora andamanensis NBRC 109075	85.47	79.86	24.3
1668.	Micromonospora violae DSM 45888	Micromonospora sediminimaris NBRC 107745	85.45	80.13	24.3
1669.	Micromonospora zingiberis PLAI 1-1	Micromonospora profundi DSM 45981	85.51	79.80	24.3
1670.	Micromonospora zingiberis PLAI 1-1	Micromonospora purpurea DSM 43036	85.90	79.88	24.3
1671.	Micromonospora zingiberis PLAI 1-1	Micromonospora tulbaghiae DSM 45142	85.89	79.96	24.3
1672.	Micromonospora andamanensis NBRC 109075	Micromonospora alfalfae MED01	85.36	79.67	24.2
1673.	Micromonospora andamanensis NBRC 109075	Micromonospora chokoriensis DSM 45160	85.38	79.71	24.2
1674.	Micromonospora andamanensis NBRC 109075	Micromonospora salmantinae PSH03	85.44	79.59	24.2
1675.	Micromonospora andamanensis NBRC 109075	Micromonospora schwarzwaldensis DSM 45708	85.68	79.51	24.2
1676.	Micromonospora andamanensis NBRC 109075	Micromonospora zamorensis DSM 45600	85.44	79.51	24.2
1677.	Micromonospora arida LB32	Micromonospora andamanensis NBRC 109075	85.36	79.61	24.2
1678.	Micromonospora aurantiaca ATCC 27029	Micromonospora jinlongensis DSM 45876	85.88	79.94	24.2
1679.	Micromonospora aurantiaca ATCC 27029	Micromonospora palythoicola S2-005	85.69	79.82	24.2
1680.	Micromonospora aurantiaca ATCC 27029	Micromonospora taraxaci DSM 45885	85.79	79.79	24.2

1681.	Micromonospora aurantiaca ATCC 27029	Micromonospora zingiberis PLAI 1-1	85.87	79.94	24.2
1682.	Micromonospora chalcea DSM 43026	Micromonospora phaseoli CGMCC 4.7038	85.71	79.85	24.2
1683.	Micromonospora chalcea DSM 43026	Micromonospora taraxaci DSM 45885	85.79	79.80	24.2
1684.	Micromonospora fiedleri MG-37	Micromonospora marina PCU 269	85.81	79.67	24.2
1685.	Micromonospora fiedleri MG-37	Micromonospora tulbaghiae DSM 45142	85.80	79.49	24.2
1686.	Micromonospora jinlongensis DSM 45876	Micromonospora andamanensis NBRC 109075	85.44	79.70	24.2
1687.	Micromonospora jinlongensis DSM 45876	Micromonospora purpurea DSM 43036	85.85	79.98	24.2
1688.	Micromonospora jinlongensis DSM 45876	Micromonospora sediminimaris NBRC 107745	85.46	80.08	24.2
1689.	Micromonospora noduli GUI43	Micromonospora sediminimaris NBRC 107745	85.43	80.10	24.2
1690.	Micromonospora orduensis S2509	Micromonospora lutea NBRC 106530	85.46	79.82	24.2
1691.	Micromonospora palythoicola S2-005	Micromonospora marina PCU 269	85.71	79.79	24.2
1692.	Micromonospora parathelypteridis DSM 103125	Micromonospora sediminimaris NBRC 107745	85.41	79.92	24.2
1693.	Micromonospora purpurea DSM 43036	Micromonospora palythoicola S2-005	85.66	79.80	24.2
1694.	Micromonospora purpurea DSM 43036	Micromonospora phaseoli CGMCC 4.7038	85.66	79.78	24.2
1695.	Micromonospora purpurea DSM 43036	Micromonospora zamorensis DSM 45600	85.79	80.10	24.2
1696.	Micromonospora robiginosa 28ISP2-46	Micromonospora sediminimaris NBRC 107745	85.73	79.73	24.2
1697.	Micromonospora sediminimaris NBRC 107745	Micromonospora alfalfae MED01	85.31	79.98	24.2
1698.	Micromonospora sediminimaris NBRC 107745	Micromonospora cabrerizensis LAH09	85.47	80.12	24.2
1699.	Micromonospora sediminimaris NBRC 107745	Micromonospora fluminis A38	85.71	79.52	24.2
1700.	Micromonospora sediminimaris NBRC 107745	Micromonospora marina PCU 269	85.67	79.85	24.2
1701.	Micromonospora sediminimaris NBRC 107745	Micromonospora trifolii NIE79	85.46	80.11	24.2
1702.	Micromonospora sediminimaris NBRC 107745	Micromonospora zamorensis DSM 45600	85.40	79.94	24.2
1703.	Micromonospora taraxaci DSM 45885	Micromonospora palythoicola S2-005	85.47	80.17	24.2
1704.	Micromonospora taraxaci DSM 45885	Micromonospora purpurea DSM 43036	85.73	79.78	24.2
1705.	Micromonospora ureilytica DSM 101692	Micromonospora palythoicola S2-005	85.43	80.18	24.2

1706.	Micromonospora vinacea DSM 101695	Micromonospora sediminimaris NBRC 107745	85.44	80.21	24.2
1707.	Micromonospora violae DSM 45888	Micromonospora andamanensis NBRC 109075	85.43	79.79	24.2
1708.	Micromonospora andamanensis NBRC 109075	Micromonospora humi DSM 45647	85.66	79.41	24.1
1709.	Micromonospora andamanensis NBRC 109075	Micromonospora saelicesensis DSM 44871	85.38	79.66	24.1
1710.	Micromonospora andamanensis NBRC 109075	Micromonospora sediminicola DSM 45794	85.75	79.62	24.1
1711.	Micromonospora andamanensis NBRC 109075	Micromonospora trifolii NIE79	85.36	79.64	24.1
1712.	Micromonospora aurantiaca ATCC 27029	Micromonospora phaseoli CGMCC 4.7038	85.65	79.84	24.1
1713.	Micromonospora chalcea DSM 43026	Micromonospora andamanensis NBRC 109075	85.61	79.24	24.1
1714.	Micromonospora chalcea DSM 43026	Micromonospora sediminimaris NBRC 107745	85.74	79.46	24.1
1715.	Micromonospora lutea NBRC 106530	Micromonospora cabrerizensis LAH09	85.39	79.73	24.1
1716.	Micromonospora lutea NBRC 106530	Micromonospora lupini JCM 16031	85.40	79.65	24.1
1717.	Micromonospora lutea NBRC 106530	Micromonospora marina PCU 269	85.71	79.55	24.1
1718.	Micromonospora marina PCU 269	Micromonospora chokoriensis DSM 45160	85.77	80.50	24.1
1719.	Micromonospora marina PCU 269	Micromonospora zamorensis DSM 45600	85.71	80.60	24.1
1720.	Micromonospora palythoicola S2-005	Micromonospora tulbaghiae DSM 45142	85.69	79.83	24.1
1721.	Micromonospora parathelypteridis DSM 103125	Micromonospora andamanensis NBRC 109075	85.45	79.37	24.1
1722.	Micromonospora profundi DSM 45981	Micromonospora palythoicola S2-005	85.28	80.08	24.1
1723.	Micromonospora robiginosa 28ISP2-46	Micromonospora andamanensis NBRC 109075	85.64	79.40	24.1
1724.	Micromonospora sediminimaris NBRC 107745	Micromonospora chokoriensis DSM 45160	85.42	80.04	24.1
1725.	Micromonospora sediminimaris NBRC 107745	Micromonospora hortensis NIE111	85.44	80.00	24.1
1726.	Micromonospora sediminimaris NBRC 107745	Micromonospora salmantinae PSH03	85.49	80.17	24.1
1727.	Micromonospora sediminimaris NBRC 107745	Micromonospora schwarzwaldensis DSM 45708	85.69	79.74	24.1
1728.	Micromonospora ureilytica DSM 101692	Micromonospora andamanensis NBRC 109075	85.39	79.52	24.1
1729.	Micromonospora ureilytica DSM 101692	Micromonospora sediminimaris NBRC 107745	85.38	80.03	24.1
1730.	Micromonospora andamanensis NBRC 109075	Micromonospora marina PCU 269	85.56	79.86	24

1731.	Micromonospora arida LB32	Micromonospora lutea NBRC 106530	85.39	79.80	24
1732.	Micromonospora aurantiaca ATCC 27029	Micromonospora sediminimaris NBRC 107745	85.66	79.45	24
1733.	Micromonospora jinlongensis DSM 45876	Micromonospora marina PCU 269	85.71	80.55	24
1734.	Micromonospora lutea NBRC 106530	Micromonospora chokoriensis DSM 45160	85.42	79.78	24
1735.	Micromonospora lutea NBRC 106530	Micromonospora fluminis A38	85.63	79.43	24
1736.	Micromonospora lutea NBRC 106530	Micromonospora humi DSM 45647	85.57	79.54	24
1737.	Micromonospora lutea NBRC 106530	Micromonospora saelicesensis DSM 44871	85.38	79.93	24
1738.	Micromonospora lutea NBRC 106530	Micromonospora salmantinae PSH03	85.45	79.83	24
1739.	Micromonospora lutea NBRC 106530	Micromonospora sediminicola DSM 45794	85.68	79.82	24
1740.	Micromonospora lutea NBRC 106530	Micromonospora zamorensis DSM 45600	85.38	79.70	24
1741.	Micromonospora marina PCU 269	Micromonospora phaseoli CGMCC 4.7038	85.58	80.09	24
1742.	Micromonospora noduli GUI43	Micromonospora lutea NBRC 106530	85.35	79.69	24
1743.	Micromonospora parathelypteridis DSM 103125	Micromonospora lutea NBRC 106530	85.39	79.53	24
1744.	Micromonospora profundi DSM 45981	Micromonospora andamanensis NBRC 109075	85.31	79.60	24
1745.	Micromonospora purpurea DSM 43036	Micromonospora andamanensis NBRC 109075	85.59	79.21	24
1746.	Micromonospora purpurea DSM 43036	Micromonospora sediminimaris NBRC 107745	85.65	79.48	24
1747.	Micromonospora sediminimaris NBRC 107745	Micromonospora saelicesensis DSM 44871	85.44	80.14	24
1748.	Micromonospora sediminimaris NBRC 107745	Micromonospora tulbaghiae DSM 45142	85.60	79.38	24
1749.	Micromonospora taraxaci DSM 45885	Micromonospora andamanensis NBRC 109075	85.38	79.55	24
1750.	Micromonospora taraxaci DSM 45885	Micromonospora marina PCU 269	85.70	80.37	24
1751.	Micromonospora taraxaci DSM 45885	Micromonospora sediminimaris NBRC 107745	85.36	80.01	24
1752.	Micromonospora tulbaghiae DSM 45142	Micromonospora phaseoli CGMCC 4.7038	85.66	79.83	24
1753.	Micromonospora vinacea DSM 101695	Micromonospora lutea NBRC 106530	85.39	79.71	24
1754.	Micromonospora violae DSM 45888	Micromonospora lutea NBRC 106530	85.39	79.87	24
1755.	Micromonospora andamanensis NBRC 109075	Micromonospora tulbaghiae DSM 45142	85.56	79.77	23.9

1756.	Micromonospora aurantiaca ATCC 27029	Micromonospora andamanensis NBRC 109075	85.58	79.27	23.9
1757.	Micromonospora jinlongensis DSM 45876	Micromonospora lutea NBRC 106530	85.39	79.76	23.9
1758.	Micromonospora lutea NBRC 106530	Micromonospora hortensis NIE111	85.32	79.58	23.9
1759.	Micromonospora lutea NBRC 106530	Micromonospora schwarzwaldensis DSM 45708	85.65	79.61	23.9
1760.	Micromonospora lutea NBRC 106530	Micromonospora trifolii NIE79	85.29	79.60	23.9
1761.	Micromonospora purpurea DSM 43036	Micromonospora lutea NBRC 106530	85.57	79.17	23.9
1762.	Micromonospora robiginosa 28ISP2-46	Micromonospora lutea NBRC 106530	85.57	79.54	23.9
1763.	Micromonospora ureilytica DSM 101692	Micromonospora lutea NBRC 106530	85.30	79.78	23.9
1764.	Micromonospora aurantiaca ATCC 27029	Micromonospora lutea NBRC 106530	85.57	79.34	23.8
1765.	Micromonospora chalcea DSM 43026	Micromonospora lutea NBRC 106530	85.62	79.35	23.8
1766.	Micromonospora lutea NBRC 106530	Micromonospora alfalfae MED01	85.24	79.75	23.8
1767.	Micromonospora profundi DSM 45981	Micromonospora sediminimaris NBRC 107745	85.24	79.65	23.8
1768.	Micromonospora taraxaci DSM 45885	Micromonospora lutea NBRC 106530	85.30	79.72	23.7
1769.	Micromonospora lutea NBRC 106530	Micromonospora tulbaghiae DSM 45142	85.49	79.40	23.6
1770.	Micromonospora profundi DSM 45981	Micromonospora lutea NBRC 106530	85.25	79.49	23.6

Table S2 ANI and dDDH values of 1770 pairs of type *Micromonospora* strains
